# Extracellular Vesicles Derived From Antral Follicles Significantly Change the Transcriptional Profile of Cumulus Cells and Oocytes During Pre‐In Vitro Maturation in Cattle

**DOI:** 10.1002/mrd.70068

**Published:** 2025-11-24

**Authors:** Gisele Zoccal Mingoti, Giovana Barros Nunes, Mirela Brochado Souza‐Cáceres, Cintia Rodrigues da Silva, Ricardo Perecin Nociti, Flávia Florêncio de Athayde, Henry David Mogollón‐García, Natália Marins Bastos, Paola Maria Silva Rosa, Lindomar de Oliveira Alves, Sérgio Antonio Garcia Pereira‐Júnior, Fernanda Fagali Franchi, João Carlos Pinheiro Ferreira, Juliano Coelho da Silveira, Marcos Roberto Chiaratti

**Affiliations:** ^1^ School of Veterinary Medicine, Laboratory of Reproductive Physiology Universidade Estadual Paulista Araçatuba Sao Paulo Brazil; ^2^ Graduate Program in Veterinary Medicine, School of Agrarian and Veterinary Sciences Universidade Estadual Paulista Jaboticabal Sao Paulo Brazil; ^3^ Departament of Genetics and Evolution, Center for Biological and Health Sciences Federal University of Sao Carlos Sao Carlos Sao Paulo Brazil; ^4^ Department of Veterinary Medicine University of Sao Paulo, Pirassununga Sao Paulo Brazil; ^5^ Department of Veterinary Surgery and Animal Reproduction, School of Veterinary Medicine and Animal Science Universidade Estadual Paulista Botucatu Sao Paulo Brazil; ^6^ Graduate Program in Pharmacology and Biotechnology, Institute of Biosciences, Universidade Estadual Paulista Botucatu Sao Paulo Brazil

**Keywords:** bovine, extracellular vesicles, follicle development, mitochondria, oocyte competence, pre‐IVM, RNA‐Seq

## Abstract

Cumulus‐oocyte complexes (COCs) used for in vitro production (IVP) of bovine embryos originate from antral follicles of different sizes, leading to variations in developmental competence. To address this, pre‐in vitro maturation (pre‐IVM) allows oocytes with additional time to acquire developmental competence. Given the role of follicular fluid‐derived extracellular vesicles (EVs) in ovarian follicle communication, which has been shown to vary in content and function across folliculogenesis, we investigated whether EVs from early versus late antral follicles influence COCs during pre‐IVM. EV supplementation significantly altered gene expression in cumulus cells and oocytes. In cumulus cells, affected pathways included MAPK signaling, Gap junctions, Cytokine‐cytokine receptor interaction, Axon guidance, cAMP, and Cushing syndrome. In oocytes, fewer genes were altered, with effects on Inositol phosphate metabolism, p53 signaling and Cholesterol metabolism. Despite these changes, no significant effects of the EV treatment were noted on oocyte chromatin configuration and developmental competence, except for a significant increase of mitochondrial membrane potential (Δψm) in blastocysts. In conclusion, EV supplementation during pre‐IVM significantly altered the transcriptional profile of COCs, with EVs from early follicles modulating the expression of genes regulating cumulus cell proliferation and gap junctions, while EVs from late follicles impacted pathways associated with meiotic resumption, cumulus cell expansion, and apoptosis. Along with improved Δψm in blastocysts, these results support a positive effect of EVs on bovine COCs, but further research is needed to better characterize the functional consequences, mainly in terms of the effects of early versus late follicle‐derived EVs on oocyte developmental potential.

## 1 Introduction

Previous studies have been essential in implementing improvements in the in vitro production (IVP) protocols of bovine embryos. However, despite these advances, embryonic developmental rates remain lower than those observed in vivo (Ferré et al. 2020; Lonergan and Fair 2016). These limitations are often attributed to the heterogeneity of the oocyte population used in IVP procedures as cumulus‐oocyte complexes (COCs) are retrieved from follicles at different stages of the growth wave. Consequently, oocytes within these follicles may be at varying stages of development regarding the acquisition of competence for meiosis resumption, fertilization, and subsequent development (Lonergan and Fair 2016; Zhang et al. 2017). In theory, oocyte competence in cattle is higher when the follicle is larger, ranging from 3 mm up to a limit of 10 mm in diameter, according to Blondin et al. (2012) and Labrecque et al. (2016). This is because communication between follicular cells and the oocyte is crucial for oocyte development, providing a favorable environment for competence acquisition (van den Hurk and Zhao 2005).

The follicular fluid, composed of hormones, antiapoptotic factors, proteins, peptides, and nucleotides, plays an essential role in maintaining oocyte quality and competence acquisition (Basuino and Silveira 2016). Composition of the follicular fluid varies according to the stage of follicular growth and is indicative of oocyte quality (Revelli et al. 2009). Extracellular vesicles (EVs) are also present in the follicular fluid, where they modulate communication between follicular cells and the oocyte. Studies have supported the successful isolation of EVs from the follicular fluid of cows, highlighting their involvement in key events of oogenesis, folliculogenesis, oocyte maturation, and embryonic development (Boyer et al. 2010; da Silveira et al. 2017; Hung et al. 2015; Morales Dalanezi et al. 2019). EVs carry a variety of substances and signaling molecules, including lipids, proteins, messenger RNA (mRNA), microRNA (miRNA), and DNA, and which have been characterized in animal reproduction due to their ability to modulate functions in target cells (Keller et al. 2006; Ludwig and Giebel 2012). For instance, miRNAs, such as those exclusive from granulosa cells described by da Silveira et al. (2012), play a fundamental role in oocytes where the absence of certain miRNAs or even the absence of Dicer, an enzyme responsible for miRNA synthesis, leads to modifications in metaphase I (MI) and II (MII) plates, highlighting the importance of miRNAs for meiotic resumption (da Silveira et al. 2018).

To improve competence of oocytes used in the IVP protocols, pre‐in vitro maturation (pre‐IVM) of COCs was previously established as a step before in vitro maturation (IVM) itself (Albuz et al. 2010). During pre‐IVM, pharmacological substances are employed to delay the breakdown of the germinal vesicle (GV) and the spontaneous resumption of meiosis in oocytes cultured in vitro. The aim is to provide additional time for oocytes to acquire developmental competence, potentially through enhanced cytoplasmic and molecular maturation. However, although it is known that EVs play an important role in modulating target cells (Keller et al. 2006; Ludwig and Giebel 2012), including improvement of developmental rates when added to IVM and in vitro culture (IVC) media (da Silveira et al. 2017), it has not yet been evaluated whether EVs can impact oocyte competence during pre‐IVM.

Recently, studies have shown that EVs recovered from follicles of different sizes modulate granulosa cell proliferation and cumulus cell expansion and gene expression (Hung et al. 2015, 2017). Indeed, the biological components of EVs from the follicular fluid were shown to differ in concentration and composition across the follicular wave (Hung et al. 2015; Navakanitworakul et al. 2016), but little is known about the effects of EVs on COCs given the few outputs investigated so far. Therefore, our hypothesis is that EVs recovered from the follicular fluid at different stages of folliculogenesis differentially modulate the competence of bovine oocytes. To tackle this hypothesis, we performed pre‐IVM in the presence of EVs derived from antral follicles at two stages of development: early versus late follicles. We aimed with this to determine whether treatment with these sources of EVs differently modulated the transcriptome of COCs and oocyte developmental competence. To that aim, we assessed the transcriptional profile, meiotic maturation and pre‐implantation development following the treatment during pre‐IVM with EVs derived from the fluid of early versus late follicles; the effect of EV supplementation was compared to a control in which pre‐IVM was performed in the absence of EVs (Figure S1). In brief, our results support a massive effect of EVs on the transcriptional profile of COCs, mainly in cumulus cells where EVs from early follicles modulated genes regulating cumulus cell proliferation and gap junctions, while EVs from late follicles affected pathways such as meiotic resumption, cumulus cell expansion and apoptosis. Although the treatment with EVs had no evident effect on oocyte chromatin configuration and developmental competence, it significantly increased mitochondrial membrane potential (Δψm) in blastocysts, supporting a positive effect of EVs on COCs.

## 2 Material and Methods

### 2.1 Experimental Design

In the present experiment, COCs were subjected to pre‐IVM in the absence (control) or presence of EVs from the follicular fluid of either early (7.0–8.5 mm) or late (≥ 12.0 mm) follicles. Afterwards, COCs were used for (i) analysis of chromatin remodeling and meiotic progression; (ii) IVP encompassing IVM, IVF and IVC; (iii) isolation of cumulus cells and oocytes at the end of pre‐IVM for analysis of the transcriptional profile by RNA sequencing (RNA‐Seq). For chromatin remodeling and meiotic progression, an additional control group represented by immature oocytes processed immediately after isolation from antral follicles was considered. Embryos obtained after IVP were assessed as for developmental rates, total number of cells, apoptosis and Δψm. The present experiment was approved by the Ethics Committee for the Use of Animals in Research of FMVZ – UNESP (authorization number: CEUA‐86/2013). All chemicals and reagents used were purchased from Sigma‐Aldrich Chemical Co. (St. Louis, MO), unless otherwise stated.

### 2.2 Isolation of Extracellular Vesicles

EVs were recovered from the follicular fluid of bovine females, as previously reported by (Ferrazza et al. 2017). Briefly, thirteen non‐lactating, healthy, and cycling Holstein cows (3 to 8 years old) were subjected to ovulation synchronization using the Ovsynch protocol (Pursley et al. 1995), with the use of an intravaginal progesterone device (Sincrogest, Ourofino, Cravinhos, Brazil). From the day of ovulation, ultrasound evaluations of the ovaries (MyLab30 Vet Gold with a 7.5 MHz linear matrix transducer, Esaote, Genoa, Italy) were performed every 12 h to monitor the emergence and development of a new follicular wave. The diameter of each follicle was calculated during ultrasound measurements, and the respective fluid was aspirated at two specific stages of wave development according to antral follicle size: early [follicular diameter between 7.0 and 8.5 mm, characterizing the pre‐deviation phase (Ginther 2016; Kulick et al. 2001)] and late [diameter ≥ 12.0 mm, characterizing the post‐deviation phase (Sartori et al. 2001; Siqueira et al. 2013)]. EVs were isolated from 1 mL of follicular fluid, which was subjected to ultracentrifugation according to the protocol described by (Morales Dalanezi et al. 2019). The follicular fluid was centrifuged successively at 300xg for 10 min, 2000x*g* for 10 min and 16,500x*g* 30 min at 4°C. The supernatant was then placed in ultracentrifuge tubes (Sorvall MTX 150 ‐ Micro‐ultracentrifuge, Thermo Scientific, Waltham, Massachusetts, USA) and ultracentrifuged at 120,000x*g* for 70 min at 4°C. The pellet was washed in PBS (pH = 7.4) and ultracentrifuged again at 120,000x*g* for 70 min at 4°C. The pellet was resuspended with 100 μL of PBS and stored at −80°C (Morales Dalanezi et al. 2019).

### 2.3 Characterization of Extracellular Vesicles

Isolated EVs were characterized based on the presence of specific proteins by western blot analysis, in relation to size and concentration based on nanoparticle tracking analysis (NTA) and by transmission electron microscopy.

Western blot analysis was performed according to the protocol described by de Ávila et al. (2020), with modifications. Each EV sample (total volume of 15 μL) was supplemented with 15 μL of lysis buffer containing protease inhibitor (RIPA, radioimmunoprecipitation assay) and placed on ice with constant agitation for total protein extraction. Subsequently, samples were applied to a 10% SDS‐PAGE polyacrylamide gel, run at 100 V for 2 h, followed by wet transfer of proteins to a nitrocellulose membrane. After blocking in 5% of nonfat dry milk in tris‐buffered saline with Tween‐20 (TBST), membranes were incubated with primary antibodies overnight at 4°C. Specific membrane proteins of EVs evaluated were ALIX (ALG‐2 interacting protein X) (rabbit, sab4200476, Sigma‐Aldrich, St. Louis, MO, USA) and CD9 (mouse, sc‐13118, Santa Cruz, CA, USA). For the negative control, the mitochondrial protein CY‐CS (Cytochrome‐c) (goat, sc‐8385, Santa Cruz, CA, USA) was used. After incubation with primary antibodies and three washes with TBST, membranes were incubated for 1 h with secondary antibodies (conjugates in horseradish peroxidase) anti‐rabbit (A0545; Sigma–Aldrich Chemical Company, St. Louis, MO), anti‐mouse (#7076S; Cell Signaling Technology, Danvers, Massachusetts, USA) and anti‐goat (SC‐2020, Santa Cruz, CA, USA). Afterwards, membranes were washed and revealed with a detection solution (170‐560, Clarity Western ECL) using the ChemiDoc MP Image System (Bio‐Rad, Hercules, CA, USA).

For analysis of particle size and concentration by NTA, 5 µL of the enriched EV samples were diluted into 495 µL of calcium and magnesium‐free PBS and analyzed using the Nanosight (NS300; NTA 3.4 Build 3.1.45; Malvern, UK). Five videos of 30 s each were captured at camera level 13 and temperature control at 37°C. The data were evaluated with a threshold of 5.

For visual inspection of EVs by transmission electron microscopy, EV samples were diluted in 50 μL of a fixative solution consisting of 0.1 M cacodylate, 2.5% glutaraldehyde, and 4% paraformaldehyde (pH 7.2–7.4) for 2 h at room temperature. Subsequently, samples were diluted with 2 mL of milli‐Q water and ultracentrifuged at 119,700 g for 70 min at 4°C. Pellets were diluted in 20 μL of milli‐Q water and placed on a pioloform‐coated copper grid for 30 min at room temperature to dry. A drop of 2% uranyl acetate was then added to the grid, and the excess was removed with filter paper. Grids were immediately assessed on a transmission electron microscope (FEI Tecnai 20; LAB6 emission; 200 kV) to obtain images.

### 2.4 Harvesting of Cumulus‐Oocyte Complexes

Ovaries of cows slaughtered in the nearby of Araçatuba, SP, Brazil, were transported to the laboratory at 30°C–35°C. COCs were obtained by aspirating antral follicles measuring 3 to 8 mm using a 10 mL syringe attached to an 18 G needle. COCs were selected in centrifuged follicular fluid supplemented with antibiotic (75 μg/mL of penicillin/streptomycin) to prevent the immediate resumption of meiosis. Only COCs that exhibited at least four layers of compact cumulus cells and cytoplasm with homogeneous granulation were selected for culture.

### 2.5 Pre‐In Vitro Maturation

Selected COCs (25 per drop) were subjected to pre‐IVM in drops of 100 µL of medium composed of TCM199 supplemented with 0.2 mM pyruvate, 25 mM sodium bicarbonate, 50 µg/mL amikacin, 0.3% fatty acid‐free BSA, 1 × 10^−4^ IU/mL recombinant human follicle‐stimulating hormone (r‐hFSH; Gonal F, Serono S.p.A.), and 100 nM natriuretic peptide type C (NPPC ‐ meiosis blocker), under mineral oil. EVs added to pre‐IVM were first isolated from 1 mL of pooled follicular fluid collected from either early (7.0–8.5 mm) or late (≥ 12.0 mm) follicles using the ultracentrifugation‐based protocol. The pellet of EVs was resuspended in 100 µL of PBS and 10 µL of this suspension was added into the pre‐IVM medium. COCs were kept in pre‐IVM at 38.5°C, with 5% CO_2_ in air and maximum humidity for 8 h, which is the maximal period that NPPC has been shown to sustain gap junction‐mediated communication between oocyte and adjacent cumulus cells (Franciosi et al. 2014). Oocyte selection and pre‐IVM were performed in serum‐free media.

### 2.6 Assessment of Meiotic Progression

Following pre‐IVM, oocytes were denuded of cumulus cells by gentle pipetting with the aid of 0.25% trypsin and a 10‐µL pipettor, washed in PBS with 0.1% polyvinyl‐pyrrolidone (PVP) and fixed in 4% paraformaldehyde (pH 7.2–7.4) for 15 min at room temperature. As a control, immature oocytes not subjected to pre‐IVM were similarly processed. Oocytes (*n* = 348) were then stained with 1 µg/mL Hoechst 33342 for 15 min, washed in PBS with 0.1% PVP and mounted onto slides with coverslips using Vectashield. A confocal microscope (Olympus), with LAS AF software, was used for analysis of chromatin configuration using a 40× objective with 1.5× zoom and excitation/emission adjusted to 420 and 480 nm, respectively. Oocytes that exhibited GV, without germinal vesicle breakdown (GVBD), were considered immature, being classified as GV0 (no evident chromatin condensation that diffuses around the nucleolus), GV1 (beginning of chromatin condensation into clumps around the nucleolus), GV2 (enhanced chromatin condensation forming a ring‐like structure around the nucleolus) or GV3 (highly‐condensed chromatin, forming a single mass, usually displaced from the nucleolus), as previously reported (Lodde et al. 2007). Oocytes with a metaphase plate, but without the first polar body were classified as MI, while those exhibiting both the metaphase plate and the first polar body were classified as MII. Oocytes not ascertained to any of these stages were regarded as degenerated.

### 2.7 In Vitro Embryo Production

For IVM, COCs (*n* = 254) were washed five times in pre‐IVM medium without NPPC and without EVs, and transferred to drops of 100 µL of IVM medium consisting of TCM199 supplemented with 0.2 mM pyruvate, 25 mM sodium bicarbonate, 50 µg/mL amikacin, 1 × 10^−^¹ IU/mL r‐hFSH, 100 IU/mL human chorionic gonadotropin (Vetecor; Hertape Calier, Juatuba, MG, Brazil), and 10% fetal bovine serum (FCS; Gibco BRL, Grand Island, NY, USA), under mineral oil. IVM was performed in an incubator for 22 h at 38.5°C, with an atmosphere of 5% CO_2_ in air and 100% humidity.

For IVF, semen straws were thawed and viable sperms separated from the diluents and cryoprotectants by centrifugation through a discontinuous Percoll density gradient (GE Healthcare, Bio‐Science, Uppsala, Sweden). Sperm concentration was adjusted to 2.5 × 10^7^ sperm/mL using fertilization medium (TALP‐FIV) supplemented with 4 µL/mL of PHE solution (2 mM penicillamine, 1 mM hypotaurine, and 250 µM epinephrine) and 10 µg/mL of heparin. Approximately, 100 × 10^3^ sperm were added to each drop of 100 µL of fertilization medium under mineral oil, where they were co‐incubated with COCs (25 per drop) in an incubator for 18 h at 38.5°C, with an atmosphere of 5% CO_2_ in air and 100% humidity.

For IVC, 90 µL drops of modified Synthetic Oviduct Fluid (mSOF) were supplemented with 0.2 mM l‐glutamine, 0.34 mM sodium citrate, 2.8 mM myo‐inositol, 2% BME essential amino acids, 1% MEM non‐essential amino acids, 0.5% BSA, and 2.5% FBS, under mineral oil. IVC was carried out in an incubator for 168 h at 38.5°C, with an atmosphere of 5% CO_2_ in air and 100% humidity. Cleavage rate was assessed 72 h post‐insemination (D3) and blastocyst rate was evaluated at 168 h (D7). Blastocysts were classified as early, full and expanded, as previously reported (Stringfellow et al. 1999).

### 2.8 Assessment of Mitochondrial Membrane Potential

For analysis of Δψm, D7 blastocysts (*n* = 98) were stained with the fluorescent probe MitoTracker Red CMXRos (Molecular Probes, Invitrogen, Oregon, USA), according to the manufacturer's instructions. Briefly, blastocysts were incubated in the dark for 30 min in mSOF medium supplemented with 500 nM MitoTracker Red CMXROS at 38.5°C. After incubation, blastocysts were washed three times in PBS with 0.1% PVP and immediately assessed under an inverted microscope equipped with epifluorescence (Olympus, IX51), with excitation at 579 nm and emission at 599 nm. Images were captured and analyzed using Q‐Capture Pro Image software (Media Cybernetics Inc., Version 5.0.1.26) for quantification of the emitted fluorescence intensity. Results were normalized by the control group and expressed as arbitrary fluorescence units (AFU).

### 2.9 Assessment of Apoptosis

For analysis of apoptosis, D7 blastocysts (*n* = 98) were subjected to the *terminal deoxynucleotyl transferase uracil nick and labeling* (TUNEL) reaction (In Situ Cell Death Detection Kit with Fluorescein, Roche Applied Science, IN, USA), which enables labeling of DNA fragmentation. Briefly, embryos were fixed in 4% paraformaldehyde for 40–60 min and permeabilized in PBS with 0.5% (v/v) Triton X‐100 and 0.1% sodium citrate for 30 min at room temperature. Subsequently, embryos were incubated in 15 μL of enzyme and buffer mixture (1:9) for TUNEL staining in a humid chamber, in the dark at 38.5°C for 1 h. Following this, embryos were stained with Hoechst 33,342 (1 μg/mL) for 30 min, washed in PBS‐PVP, and mounted on slides with coverslips. Samples were immediately evaluated under an inverted microscope equipped with epifluorescence (Olympus, IX51; excitation 510–550 nm and emission 590 nm) and the number of cells depicting DNA fragmentation was assessed in relation to the total number of cells.

### 2.10 RNA Sequencing

At the end of pre‐IVM, groups of 20 COCs had oocytes and cumulus cells dissociated by gentle pipetting. The entire pool of cumulus cells from 20 COCs was sampled into a single tube while groups of five oocytes were sampled per tube. Four biological replicates were considered for the control group, and eight replicates for each group (early and late) treated with EVs. Bulk RNA‐Seq was performed as previously described (Alcantara da Silva et al. 2024). Briefly, cDNA synthesis and amplification were carried out using the SMART‐Seq HT kit (Takara Bio, Kusatsu, Japan), while libraries were prepared using the Nextera XT DNA Library Prep (Illumina, San Diego, CA, USA). Sequencing was performed on a NextSeq. 550 (Illumina) considering a minimum of 10 million reads (1 × 75 bp) per sample.

Read quality was assessed using the FastQC software (http://www.bioinformatics.babraham.ac.uk/projects/fastqc/) and reads with adapters and phread score below 33 were removed using the Tringalore software (https://zenodo.org/records/7598955), which is a wrapper around Cutadapt (Martin 2011), and FastQC to consistently apply adapter and quality trimming to FastQ files. Reads were mapped on the bovine reference genome ARS‐UCD1.2 (Ensembl and NCBI) using the Star software (Dobin et al. 2013). Gene identification and quantification were performed using the feature Counts tool implemented in the R subread package (Liao et al. 2014, 2019). Reads with ambiguous mapping were discarded. Only genes with higher than 10 read counts in all samples of a specific group were evaluated for statistical significance. Once the genes were identified, differentially expressed genes (DEGs) were determined using the DESeq. 2 package (Love et al. 2014), considering adjusted *p*‐value < 0.05 and absolute |log2 fold‐change | > 0.6. Additionally, using the filter By Expr function from the edge R package (Robinson et al. 2010), genes were also considered expressed if identified as exclusively expressed, meaning expressed only in one group (expressed in all samples of a group) and not expressed in the other group considered in the contrast (count equal to zero in all samples of that group). The raw FastQ data and normalized read counts were deposited at Gene Expression Omnibus (GEO) under accession number GSE308522. DEGs are presented in Tables S1–6.

For functional annotation, we considered both DEGs and exclusively expressed genes. The list of genes used in this analysis was obtained by considering all contrasts: early EVs versus control, late EVs versus control, and early versus late EVs. Functional annotation was done based on the KEGG database (https://www.kegg.jp), using the bovine organism as background and all genes considered expressed in our samples and annotated at the org.Bt.eg.db package, with the help of the cluster Profiler package (Yu et al. 2012). Pathways were explored using the Pathview package (Luo and Brouwer 2013). All data were analyzed using the R studio version 4.9, as follows: first, classification and intensity were analyzed, and then differential expression between the groups was assessed.

### 2.11 Statistical Analysis

Each experiment was carried out considering a minimum of four replicates. In each replicate, a drop containing 25 COCs or presumptive zygotes was taken as an experimental unit for analysis of meiotic stage and embryonic developmental rates. Mean comparisons were performed using analysis of variance (ANOVA), followed by Tukey's post‐hoc test. For samples with non‐parametric distribution, the Kruskal‐Wallis test was used. All analyses were performed in the R Studio version 4.9, considering *p* < 0.05 as significant. All values were presented as mean ± standard error of the mean (SEM).

## 3 Results

### 3.1 Characterization of EVs Recovered From Follicular Fluid

EVs isolated from the follicular fluid were characterized to ascertain the efficacy of the isolation protocol. Using western blot analysis, the presence of ALIX (an endosomal pathway marker) and CD9 (a membrane protein belonging to the tetraspanin family) was detected in both EVs and follicular cells (Figure S2A), as expected. CYCS (a negative control protein for EVs), which integrates the mitochondrial respiratory complex, was only present in follicular cells (Figure S2A), confirming the absence of cellular contaminants in EVs. Based on NTA (Figure S2B–D), there was also no difference (*p* > 0.05) between groups in terms of EV size (Early: 141.2 ± 8.5 nm; Late: 156.7 ± 6.35 nm) and particle concentration (Early: 4.62 × 10¹² ± 3.17 × 10¹²/mL; Late: 4.26 × 10¹² ± 4.63 × 10¹¹/mL). Finally, EV morphology and size were evaluated through transmission electron microscopy. Images indicated morphology of cup‐shaped particles and a diameter of about 50–150 nm (Figure S2E), typical of EVs (Godakumara et al. 2021; Skog et al. 2008; Taylor and Gercel‐Taylor 2023).

### 3.2 Evaluation of Oocyte Meiotic Progression

**Figure 1:**
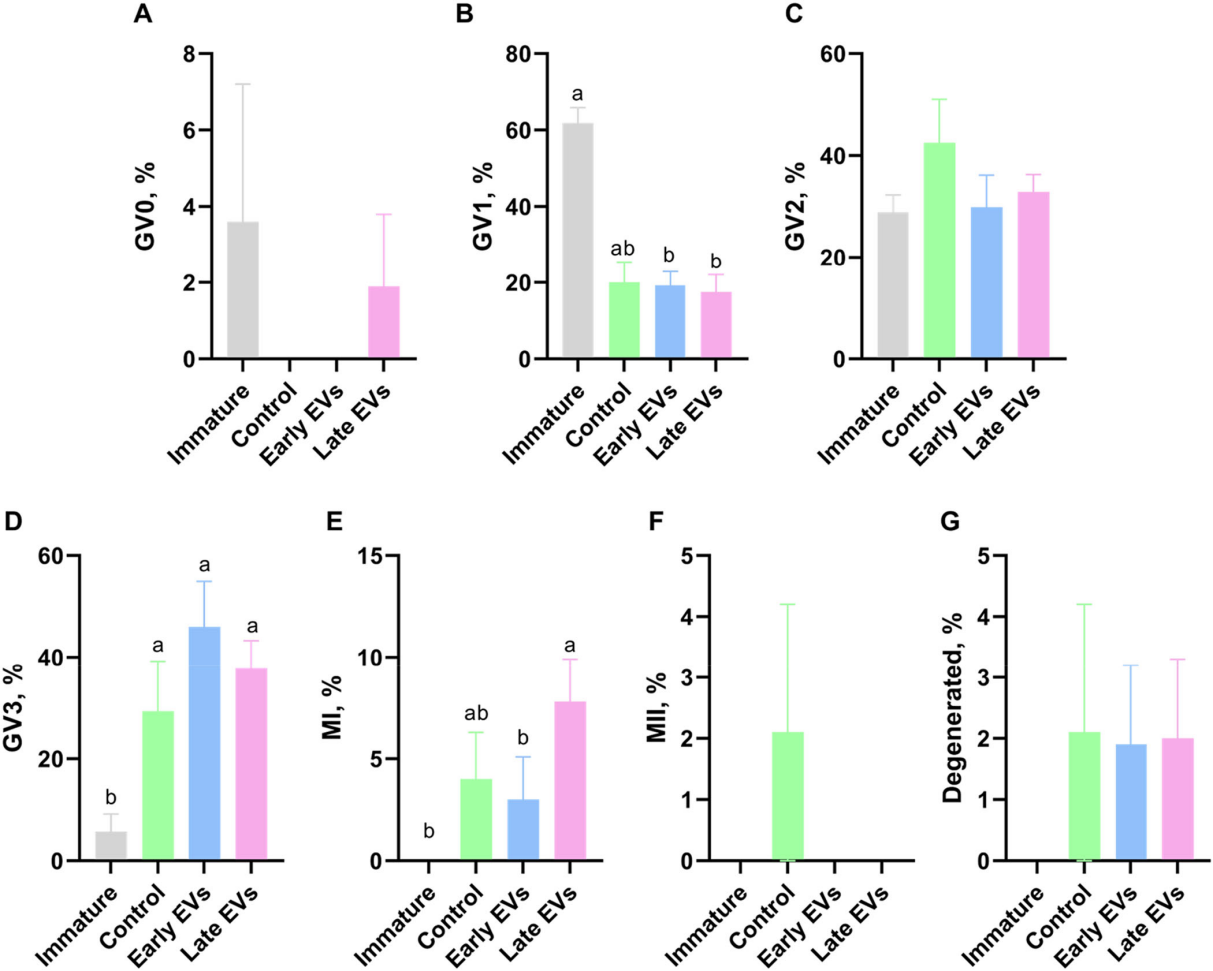
Effect of extracellular vesicle (EV) supplementation during pre‐IVM on oocyte meiotic progression. Pre‐IVM was performed in the absence (control; *n* = 51) or presence of EVs obtained from the follicular fluid of either early (*n* = 92) or late (*n* = 103) antral follicles. The immature group was not subjected to pre‐IVM (*n* = 52). The following P values were found for each meiotic stage analyzed: (A) Germinal vesicle 0 (GV0), P = 0,0069; (B) GV1, *p* = 0.0206; (C) GV2, *p* = 0.4765; (D) GV3, *p* = 0.0018; (E) Metaphase I (MI), *p* = 0.0559; (F) MII, *p* = 0.1718; (G) Degenerated oocytes, *p* = 0.7438. Different letters indicate statistical difference among groups within the stage, according to Tukey's post‐hoc test (*p* < 0.05).

To evaluate the effect of EV supplementation during pre‐IVM, oocyte meiotic maturation was assessed at the end of the pre‐IVM culture (Figure 1). As expected, most immature oocytes, which were not subjected to pre‐IVM and were assessed immediately after harvesting, showed meiosis configuration at GV1 (61.53%; Figure 1B), differing from the remaining groups (*p* < 0.05). In turn, most oocytes subjected to pre‐IVM were at GV2 and GV3, indicative of a successful meiotic block to prevent GVBD while also enabling progression of the chromatin compaction status compared to immature oocytes and oocytes subjected to IVM for 8 h (Figures 1C,D and S3). Despite this, the percentage of oocytes at GV1, GV2, GV3 and MI was not affected by the treatment with EVs, regardless of using EVs from early or late antral follicles, when compared with the control in which pre‐IVM was performed in the absence of EVs (Figure 1B–E). On the other hand, supplementation with EVs completely prevented conclusion of the first meiotic division, compared to a rate of 2.1% of MII oocytes in the control group (Figure 1F). The treatment with early versus late EVs only slightly differed regarding the rate of MI oocytes (Figure 1E). Together, these results indicate a minimal effect of EVs on meiotic maturation following pre‐IVM.

### 3.3 Evaluation of Embryonic Developmental Rates, Cell Number, Apoptosis and Δψm

**Figure 2:**
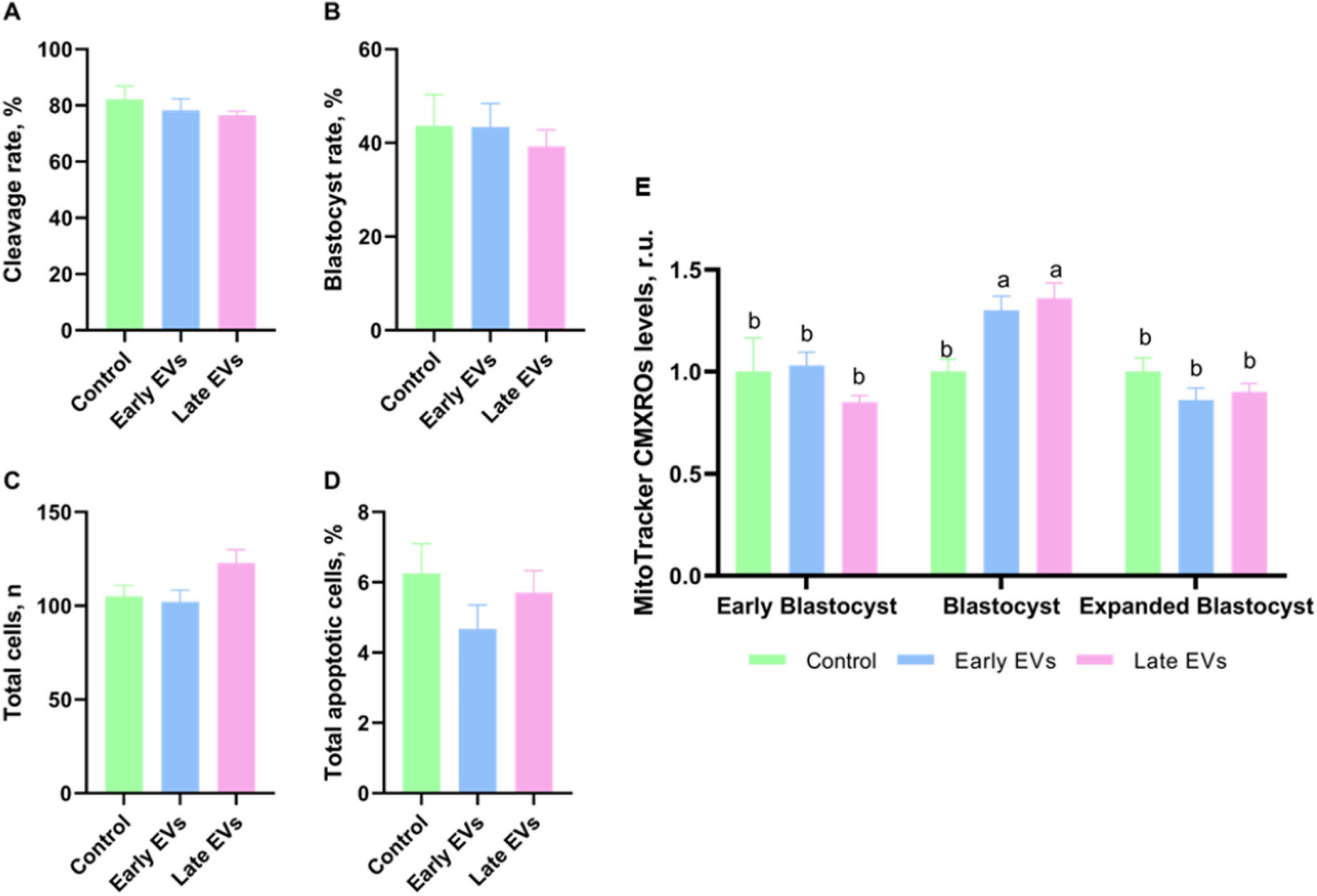
Effect of extracellular vesicle (EV) supplementation during pre‐IVM on embryo production. Pre‐IVM was performed in the absence (control; *n* = 50) or presence of EVs obtained from the follicular fluid of either early (*n* = 102) or late (*n* = 102) antral follicles. Following pre‐IVM, COCs were subjected to IVP for analysis of cleavage (A) on day (D) 3 and blastocyst rate (B). C–E) Total cell number (C), apoptosis (D) and mitochondrial membrane potential (Δψm; E) on D7 of IVC. In (A–D), no statistical difference was found among groups. In (E), the following P values were found: *p* = 0.6963 (experimental group); *p* < 0.001 (blastocyst stage); *p* = 0.0041 (experimental group x blastocyst stage). Different letters among groups indicate significant statistical difference (*p* < 0.05) within blastocyst stage, according to Tukey's post‐hoc test.

Following pre‐IVM supplemented with EVs, COCs were subjected to IVP to assess a potential effect on embryo development. However, no effect of EV supplementation, regardless of using EVs from either early or late antral follicles, was seen when considering cleavage and blastocyst rates (Figures 2A,B and S4). Analysis of total cell number and apoptosis rate in blastocysts also did not reveal any effect of EVs when compared to a control in which pre‐IVM was performed in the absence of EVs (Figure 2C,D). It is worth noting that the absence of effect on total cell number and apoptosis in blastocysts was confirmed even when considering the effect of blastocyst kinetics (Figure S5).

Blastocysts were also stained with MitoTracker CMXRos for analysis of Δψm (Figures 2E and S6). A significant interaction between experimental group and blastocyst kinetics was found in this case, requiring analysis of Δψm for each blastocyst stage separately. As a result, no statistical difference was found among experimental groups for early and expanded blastocysts. However, EV supplementation, regardless of considering EVs from early or late follicles, significantly increased Δψm in blastocysts, compared to the control group (Figure 2E).

In summary, EV supplementation during pre‐IVM had no effect on embryo production, except for increasing Δψm in blastocysts.

### 3.4 Transcriptomic Analysis of Oocytes and Cumulus Cells

**Figure 3:**
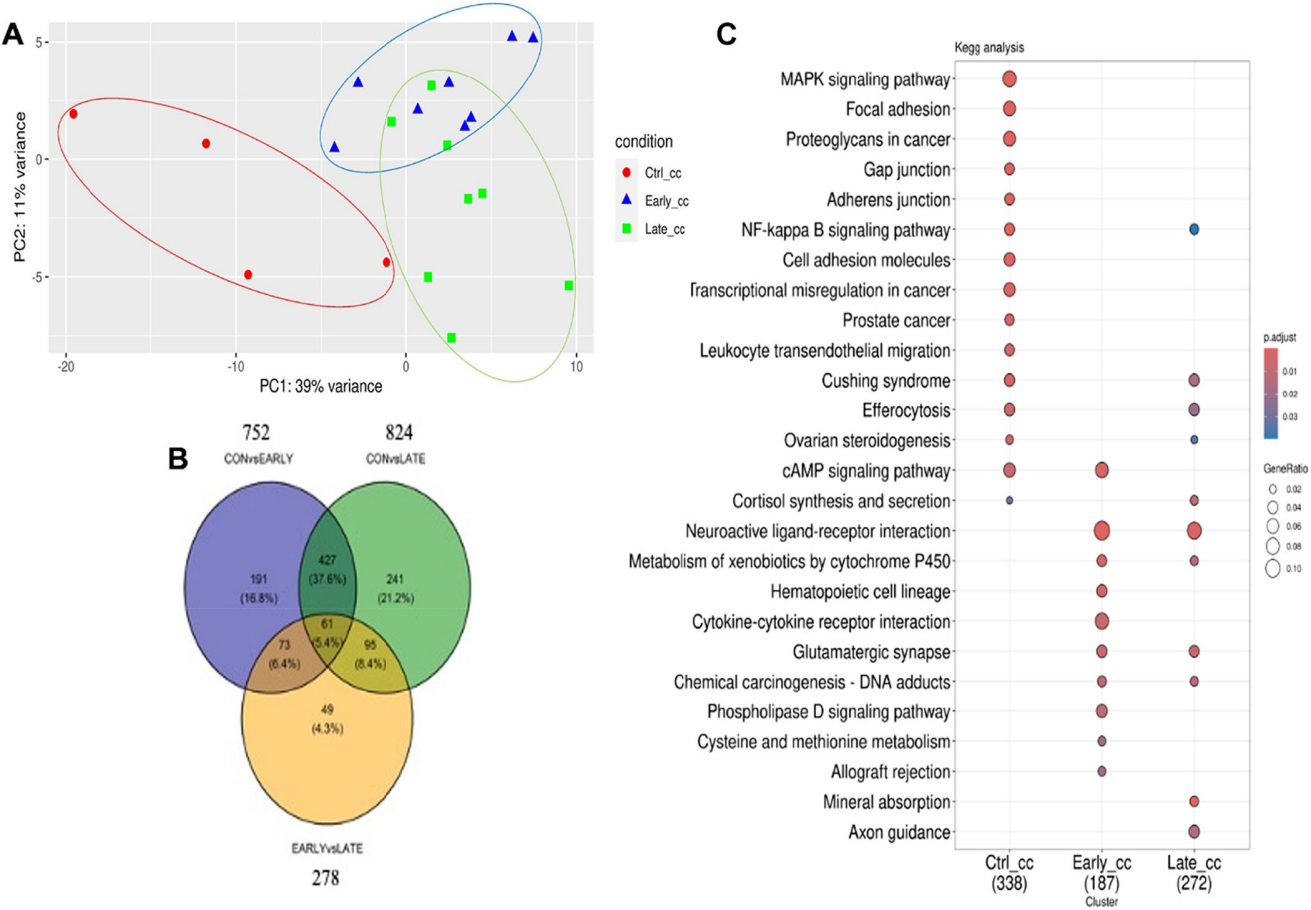
Effect of extracellular vesicle (EV) supplementation during pre‐IVM on the transcriptional profile of cumulus cells. Pre‐IVM was performed in the absence (control) or presence of EVs obtained from the follicular fluid of either early or late antral follicles. Following pre‐IVM, cumulus cells were isolated from COCs and subjected to bulk RNA sequencing (RNA‐Seq). (A) Principal Component Analysis (PCA) of the RNA‐Seq data. Cumulus cells of the control group are depicted in red while cumulus cells treated with EVs derived from early and late antral follicles are depicted in blue and green, respectively. Ellipses show a 95% confidence interval of each clustering. (B) Venn diagram showing the number of differentially expressed genes (DEGs) and exclusively expressed genes for the contrasts of interest: control x early, control x late and early x late. (C) Functional enrichment of DEGs and exclusively expressed genes based on the KEGG database. Dots indicate pathways that are enriched in each group based on the list of DEGs and exclusively expressed genes between all contrasts: early EVs versus control, late EVs versus control, early versus late EVs.

Given the minor effect of EVs on oocyte nuclear maturation and embryo production, we investigated further their effect on COCs. To that aim, COCs treated with EVs had cumulus cells and oocytes harvested at the end of pre‐IVM for analysis of the transcriptional profile by bulk RNA‐Seq. Among other effects, EVs have been shown to significantly change the transcriptome of target cells, including COCs, when used during IVM (Benedetti et al. 2024; Gabryś et al. 2024). In agreement with this, a Principal Component Analysis (PCA) based on the RNA‐Seq data of cumulus cells indicated a clear effect of EVs compared to the control group in which pre‐IVM was performed in the absence of EVs (Figure 3A). In comparison, the transcriptional profile of cumulus cells treated with early versus late EVs was less divergent (Figure 3A). Accordingly, 752 and 824 DEGs and exclusively expressed genes were found when comparing early and late EVs against the control, respectively, while early and late EVs differed by 278 DEGs and exclusively expressed genes (Figure 3B and Tables S1–3). Indeed, 427 DEGs and exclusively expressed genes were shared by the treatments with early and late EVs, when compared to the control group, while 191 and 241 DEGs and exclusively expressed genes were exclusive of each treatment, respectively (Figure 3B).

To better understand the transcriptional change induced in cumulus cells by EVs, DEGs and exclusively expressed genes from each contrast were subjected to function enrichment analysis based on the KEGG database. As a result, we highlight an enrichment for the following pathways: MAPK signaling, Gap junction, Cytokine‐cytokine receptor interaction, Axon guidance, Cushing syndrome, and cAMP signaling (Figure 3C). While the MAPK and Gap junction pathways were significantly enriched only in the control group, the Cytokine‐cytokine receptor interaction and Axon guidance pathways were exclusively enriched in cumulus cells treated with early and late EVs, respectively. In addition, the Cushing syndrome pathway was not enriched only in cumulus cells treated with early EVs, while the cAMP pathway was not enriched only in cumulus cells treated with late EVs.

**Figure 4:**
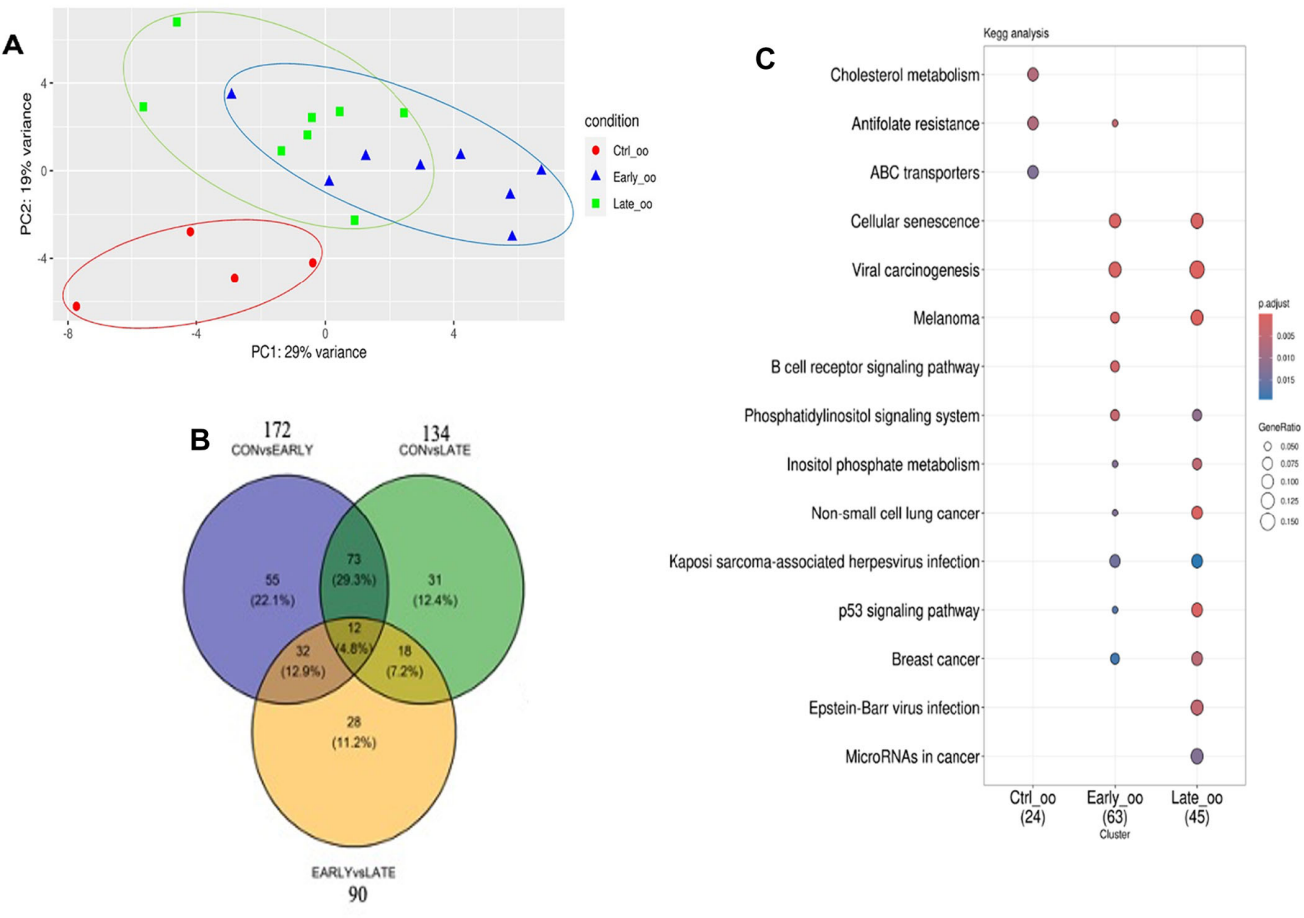
Effect of extracellular vesicle (EV) supplementation during pre‐IVM on the transcriptional profile of oocytes. Pre‐IVM was performed in the absence (control) or presence of EVs obtained from the follicular fluid of either early or late antral follicles. Following pre‐IVM, oocytes were denuded from cumulus cells and subjected to bulk RNA sequencing (RNA‐Seq). (A) Principal Component Analysis (PCA) of the RNA‐Seq data. Oocytes of the control group are depicted in red while oocytes treated with EVs derived from early and late antral follicles are depicted in blue and green, respectively. Ellipses show a 95% confidence interval of each clustering. (B) Venn diagram showing the number of differentially expressed genes (DEGs) and exclusively expressed genes for the contrasts of interest: control x early, control x late and early x late. (C) Functional enrichment of DEGs and exclusively expressed genes based on the KEGG database. Dots indicate pathways that are enriched in each group based on the list of DEGs and exclusively expressed genes between all contrasts: early EVs versus control, late EVs versus control, early versus late EVs.

Similarly to cumulus cells, PCA analysis indicated that EV supplementation changed the transcriptional profile of oocytes, compared to the control group (Figure 4A). Again, this effect was independent of using EVs derived from early or late antral follicles. On the other hand, the number DEGs and exclusively expressed genes in oocytes was smaller compared to cumulus cells as 172 and 134 genes, respectively, were altered in oocytes treated with early and late EVs, compared to the control group; only 90 DEGs and exclusively expressed genes were found when comparing oocytes treated with EVs from early versus late antral follicles (Figure 4B). Analysis of DEGs and exclusively expressed genes on the KEGG database indicated several enriched pathways shared by the two EV treatments, compared to the control group, such as the Inositol phosphate metabolism and p53 signaling (Figure 4C). However, it is also worth noting that the Cholesterol metabolism was enriched in the control group but not in oocytes treated with EVs (Figure 4C).

To sum up, the use during pre‐IVM of EVs derived from antral follicles had a minor effect on oocyte nuclear configuration and embryo production but changed the transcriptional profile of cumulus cells and oocytes, resulting in blastocysts with increased Δψm.

## 4 Discussion

In the present study, we aimed to evaluate the effects during pre‐IVM of EVs recovered from the follicular fluid of early versus late antral follicles on meiotic and developmental competence as well as on the transcriptome of bovine COCs.

Oocytes harvested from antral follicles vary as for the pattern of chromatin condensation, which is intrinsically linked to the transcriptional activity (Lodde et al. 2007) and can affect oocyte developmental competence due to an effect on mRNA and protein stockpiling. Oocytes with diffuse chromatin configuration (GV0) exhibit intense transcriptional activity; however, they still lack meiotic and developmental competence. On the other hand, oocytes at GV1 already acquired developmental competence but have reduced transcriptional activity due to the presence of chromatin condensation foci. With increased condensation (GV2 and GV3, respectively), oocyte transcriptional activity is completely halted while developmental competence is increased (Lodde et al. 2007, 2008).

In this sense, pre‐IVM is an interesting approach to provide oocytes harvested at early GV stages with additional time to progress to GV2/GV3, potentially enhancing oocyte competence (Dieleman et al. 2002; Sánchez and Smitz 2012). As expected, our present results demonstrate that pre‐IVM for 8 h was effective in preventing meiotic resumption as only 6.1% of the control oocytes progressed to MI/MII stages, compared to 37.3% when considering oocytes subjected to IVM for equivalent time. On the other hand, most oocytes subjected to pre‐IVM progressed to GV2 (42.5%) and GV3 (16.7%), compared to only 28.9% and 5.7%, respectively, at the time of harvesting. Thus, pre‐IVM was effective in enabling oocyte progression to later GV stages while preventing meiotic maturation.

Given that follicular fluid‐derived EVs from different sized follicles differentially stimulated granulosa cell proliferation, expansion and gene expression (Hung et al. 2015, 2017), EVs from early versus late antral follicles were used herein during pre‐IVM to investigate the effect on the oocyte. As a result, none of the oocytes treated with EVs reached the MII stage at the end of pre‐IVM, compared to 2.1% in the control group. It was previously shown that follicular fluid‐derived EVs contain c‐type natriuretic peptide (CNP or NPPC) that binds the natriuretic peptide receptor subtype 2 (NPR2) on granulosa cells leading to increased levels of cGMP and inhibition of phosphodiesterase 3 A in oocytes (Pioltine et al. 2020). Therefore, this result suggests that EVs contributed to promoting a more effective meiotic block during pre‐IVM. However, no other effect of EV supplementation on oocyte chromatin configuration was noted. In comparison, the RNA‐Seq data revealed a pronounced effect of EVs on the transcriptional profile of cumulus cells and oocytes. In both cases, the effect of EV supplementation was larger than the effect of EV source, compared to the control, but the number of DEGs and exclusively expressed genes was higher in cumulus cells than oocytes.

Functional enrichment analysis based on DEGs and exclusively expressed genes in cumulus cells pointed to a major effect of EVs on COCs. For example, transcripts regulating the Gap junction and the MAPK signaling pathways were enriched in cumulus cells of the control group, but not in those treated with EVs. Given that transcripts enriched in these pathways were all increased in EV‐treated cumulus cells, it suggests that EV supplementation stimulated these processes, agreeing with an earlier report that EVs modulate granulosa cell proliferation through miRNAs targeting transcripts belonging to the MAPK signaling (de Ávila et al. 2020; Hung et al. 2017). Gap junctions connecting mural granulosa cells, cumulus cells and the oocyte allow the transfer of small molecules (e.g., amino acids, metabolites and ions) necessary for oocyte development and meiotic block (Martinez et al. 2023). Treatment with early EVs increased the expression of *MET*, *TUBA1C*, *TUBB4A*, *TUBB2A* and *LOC112443216*, while late EVs only increased the expression of *TJP1* and *LOC112443216*, suggesting a stronger effect of early than late EVs on gap junctions. In turn, the effect of early and late EVs on MAPK signaling was more balanced, with each treatment upregulating eight genes in this pathway. Early EVs, for instance, increased the expression in cumulus cells of *KITLG*, an effect that is predicted to be positive to COCs given the role of KITLG in determining granulosa cell proliferation and regulating apoptosis (Cho et al. 2008; Panwar et al. 2023). On the other hand, *EREG*, which is expressed by granulosa cells in pre‐ovulatory follicles, stimulating oocyte maturation, cumulus cells expansion and ovulation (Dos Santos et al. 2018; Procházka et al. 2011), was increased in cumulus cells treated with late EVs, but not with early EVs. Other genes in the MAPK signaling upregulated in cumulus cells by both sources of EVs were *CACNA1I*, *RASA2*, *PTPRR* and *BDNF*, while *MET*, *RPS6KA1* and *MAPKAPK3* were upregulated only by early EVs and *GDNF*, *NFKB2* and *NFKB1* were upregulated only by late EVs. Among these, it is worth mentioning that BDNF stimulates proliferation and progesterone synthesis in granulosa cells, while GDNF promotes cell‐cell interaction and enhances oocyte competence (Chen et al. 2019; Dole et al. 2008; Kawamura et al. 2008; Valleh et al. 2016; Zheng et al. 2023). Also, there is evidence of miRNAs targeting mRNAs of the NFKB signaling pathway to regulate granulosa cell apoptosis (Sirotkin et al. 2015; Tan et al. 2022; S. Yang et al. 2011).

In comparison to the above pathways, the enrichment analysis also pointed to a more uneven effect of early versus late EVs on pathways such as the Cytokine‐cytokine receptor interaction and the Axon guidance. Cytokines transcripts are upregulated during follicular development (Mogollón García et al. 2024) and play significant roles in cell‐cell signaling between follicle somatic cells and the oocyte, with implications to follicle survival and apoptosis (Field et al. 2014). Moreover, oocyte development is affected by cytokines secreted by macrophages in the ovary (Ingman and Jones 2008). In humans, altered cytokines have been shown to contribute to infertility linked to polycystic ovary syndrome (Omidvar‐Mehrabadi et al. 2024). Here, *CXCR6*, *BMP4* and *LOC529196*, belonging to the Cytokine‐cytokine receptor interaction pathway, were decreased by early EVs in cumulus cells, while *GRO1* was increased by late EVs and *IL2RB*, *TGFB3*, *CSF2RA* and *IL3RA* were decreased by both early and late EVs. Among these, *BMP4* has been shown to increase apoptosis and decrease proliferation when downregulated in cumulus cells (Tian et al. 2022). Also, *TGFB3* has been shown to stimulate NPPC secretion (Yang et al. 2019). In turn, late EVs decreased in cumulus cells *NTNG1*, *WNT5A* and *SEMA6C* belonging to the Axon guidance. Although little is known about this pathway in female reproduction, early reports have indicated it can regulate follicular function and vascular development. This is the case of NTN1, for instance, which can inhibit granulosa cell viability and estradiol 17β levels, while stimulating progesterone synthesis (Baioni et al. 2010; Basini et al. 2011). Moreover, WNT5A has an important function in cumulus cell expansion (Niu et al. 2022).

The enrichment analysis also indicated that the Cushing syndrome pathway was enriched in cumulus cells of the control and late EV groups, while the cAMP pathway was enriched in the control and early EV groups. The Cushing syndrome pathway points to a possible stress response driven by EVs. In agreement with this, *AHR*, known as an environmental sensor, was upregulated by both sources of EVs, with potential consequences to follicle growth and oocyte maturation (Benedict et al. 2003; Pocar et al. 2020). Similarly, both treatments also upregulated *CDKN1A*, referred to regulate cumulus cell proliferation and DNA damage (Jiang et al. 2015; Liu et al. 2023; Wang et al. 2015), and downregulated *PDE11A*, which, along with other phosphodiesterases, hydrolyzes cyclic nucleotides to promote meiotic resumption in oocytes (Gupta et al. 2017). Also, late EVs downregulated *WNT5A*, required for normal follicle development (Abedini et al. 2016), while early EVs upregulated *CYP17A1*, the rate‐limiting enzyme for the formation of androgens in the follicles (Chen et al. 2022). With respect to the cAMP pathway, it is worth noting that both EV sources downregulated *FSHR* in cumulus cells, suggesting that the responsiveness of cumulus cells to FSH is modulated by EVs, with potential consequences to follicular growth and oocyte competence (Anazawa et al. 2025; Caixeta et al. 2009; Khan et al. 2015; Khurchabilig et al. 2020). Also, *PDE4B* was upregulated, indicative of an effect of both EV sources on cyclic nucleotide levels and meiotic resumption in oocytes, mainly when late EVs were used.

With respect to oocytes, the RNA‐Seq analysis indicated a more modest effect of EVs on the transcriptome, which was expected given that oocytes were enwrapped by a zona pellucida and several layers of cumulus cells. In spite of this, dozens of genes were altered in oocytes, mainly when regarding the use of early EVs. Among altered pathways, both treatments with EVs were enriched for the Inositol phosphate metabolism and p53 signaling. *CDK6* and *CCND2*, for instance, which regulate early phases of G1 (Malumbres and Barbacid 2005), were downregulated by both early and late EVs, while *GADD45B*, which regulates a response to stress (Palomer et al. 2024), was decreased by early EVs, compared to the control. Also, *CDKN2A*, which encodes tumor suppressor proteins regulating G1/S transition (Kreuger et al. 2023), was upregulated by early EVs. In turn, late EVs downregulated *SIVA1* that is a proapoptotic gene activated by p53 (Vachtenheim, Jr et al. 2018). Another pathway enriched in control, but not in EV‐treated, oocytes was the Cholesterol metabolism due to upregulation of *APOH* by early and *LRP1* by late EVs. APOH is an apolipoprotein that can bind free fatty acids and regulates the activity of proteins involved in cholesterol metabolism (Deng et al. 2022). LRP1 is a low‐density lipoprotein receptor with prominent functions in endocytosis, lipid metabolism, energy homeostasis and signal transduction (Lin et al. 2017).

Taken together, the results of the present study revealed an evident effect of EVs on the transcriptional profile of COCs, despite not clearing affecting oocyte chromatin condensation. This finding is supported by reports in which follicular fluid‐derived EVs were added during bovine IVM (Pioltine et al. 2020) and might be explained by the uptake of EVs by cumulus cells (Hung et al. 2017). In addition, the effect of EVs on the transcriptional profile differed according to follicle source, with early follicles modulating genes regulating cumulus cell proliferation and gap junctions (e.g., *KITLG*, *MAPKAPK3*, *TUBA1C*, *TUBB4A*, *TUBB2A*, *BMP4* and *CYP17A1*), while genes altered by late follicles mainly affected processes related to late stages of folliculogenesis such meiotic resumption, cumulus cell expansion and apoptosis (e.g., *EREG*, *GDNF*, *NFKB1*, *NFKB2*, *NTNG1*, *WNT5A*, *PDE4B* and *SIVA1*). Indeed, these results agree with a report by Navakanitworakul et al. (2016) showing that EVs from small follicles are enriched with miRNAs associated with cell proliferation pathways, while EVs from large follicles are mainly associated with inflammatory response pathways. In particular, we found that EVs from early follicles upregulated the expression of tubulin genes involved with gap junctions, suggesting these EVs promoted a greater cytoplasmic connection between cumulus cells (Martinez et al. 2023). This finding aligns with previous reports showing that EVs modulate granulosa cell proliferation and cumulus cell expansion (Hung et al. 2015, 2017), potentially promoting a positive effect on oocyte developmental competence.

In keeping with a potential effect of EVs on oocyte competence, blastocysts obtained from EV‐treated COCs depicted increased Δψm, indicative of an effect of EVs on mitochondrial activity that persisted until the blastocyst stage. During the preimplantation development, there is a significant increase in oxygen uptake and oxidative phosphorylation to support blastocoel formation and cell proliferation (Hashimoto et al. 2017; Trimarchi et al. 2000). This supports a positive effect of EVs on blastocysts, even though the ‘quiet embryo’ hypothesis postulates that over‐activation of mitochondrial function might have detrimental consequences to embryos (Leese et al. 2007). Accordingly, the Inositol phosphate metabolism, which is involved in energetic homeostasis, cell growth, nutrient uptake and mitochondrial metabolism (Tu‐Sekine and Kim 2022), was differentially modulated in oocytes treated with EVs. Similarly, the Cholesterol metabolism, which is closely linked to mitochondria (Goicoechea et al. 2023), was enriched in untreated oocytes, providing an additional clue on how the transcriptional changes induced by EVs in COCs translated to an effect on Δψm in blastocysts. However, one cannot exclude that the effect on blastocysts resulted from an effect on oocyte competence rather than a direct effect on mitochondria. In keeping with this, pathways that are determinant to oocyte competence such as Gap junction and the MAPK signaling were similarly modulated by EVs in cumulus cells by both treatments. Thus, despite the differences in the transcriptome profile of cumulus cells and oocytes, similar embryo rates and increased Δψm are supportive of a similar effect of early versus late EVs which, on overall, can be taken as positive to the oocyte. Further studies, including analysis of embryo markers of developmental capacity and pregnancy rates, are, however, needed to confirm this assumption.

Limitations of the study. IVM was conducted in the present study based on a system with FCS, which might have affected some of our conclusions given the presence of EVs in FCS. However, to minimize this effect a same batch of FCS was used in all groups throughout the study and pre‐IVM was conducted without FCS. Furthermore, COCs were collected for the RNA‐Seq analysis before IVM, not being therefore affected by a possible effect of FCS. Another limitation is the use of ultracentrifugation for EV isolation, which might affect downstream functional applications of EVs due to co‐precipitation of protein aggregates and other contaminants. However, ultracentrifugation enables large‐scale reproducible isolation of EVs, as needed for the present study. Additionally, this is the most common method of choice and standardized protocol for EV isolation, including studies in which EVs were isolated from the follicular fluid to modulate oocyte's competence. Finally, new studies are required to further investigate potential consequences of EV supplementation on the oocyte's developmental competence, specially evaluating EV's contents to understand the potential of the EVs according to different follicular origins. The RNA‐Seq study revealed that the EV treatment triggered an important transcriptomic response, but this did not reflect on changes on oocyte chromatin configuration or embryonic developmental rates. Therefore, additional analysis involving, for instance, characterization of the embryonic transcriptome and pregnancy rates are needed to better test the effect of EV supplementation on the oocyte.

In conclusion, supplementation of pre‐IVM with follicular fluid‐derived EVs had a pronounced effect on the transcriptional profile of COCs, with EVs from early follicles modulating genes associated to cumulus cell proliferation and gap junctions, while EVs from late follicles mainly affected pathways such as meiotic resumption, cumulus cell expansion and apoptosis. Although no evident effect was seen on oocyte chromatin configuration and competence, the EV treatment significantly increased Δψm in blastocysts. Altogether, the results from this study support a positive effect of EV supplementation on the COC, but further research is needed to better characterize functional consequences, mainly in terms of the effect of early versus late follicle‐derived EVs on oocyte developmental potential.

## Supporting information


**Figure S1:** Representative scheme of the experimental design. **Figure S2:** Characterization of extracellular vesicles (EVs) isolated from the follicular fluid of either early and late antral follicles. **Figure S3:** Effectiveness of pre‐IVM in enabling meiotic progression of oocytes to later stages of prophase. **Figure S4:** Effect of extracellular vesicles (EV) supplementation during pre‐IVM on blastocyst kinetics. **Figure S5:** Effect of extracellular vesicle (EV) supplementation during pre‐IVM on blastocyst cell number and apoptosis. **Figure S6:** Effect of extracellular vesicle (EV) supplementation during pre‐IVM on mitochondrial membrane potential (Δψm).

**Table S1:** Differentially expressed genes in cumulus cells of Control vs. Early EVs.

**Table S2:** Differentially expressed genes in cumulus cells of Control vs. Late EVs.

**Table S3:** Differentially expressed genes in cumulus cells of Early EVs vs. Late EVs.

**Table S4:** Differentially expressed genes in oocytes of Control vs. Early EVs.

**Table S5:** Differentially expressed genes in oocytes of Control vs. Late EVs.

**Table S6:** Differentially expressed genes in oocytes of Early EVs vs. Late EVs.
