# Supplementary material for: Extracellular Vesicles Derived From Antral Follicles Significantly Change the Transcriptional Profile of Cumulus Cells and Oocytes During Pre‐In Vitro Maturation in Cattle
Source: Mol Reprod Dev. 2025 Nov 24;92(11):e70068. doi: 10.1002/mrd.70068 (PMC12645189; doi:10.1002/mrd.70068)
Supplement: Supplementary file 1 — Figure S1: Representative scheme of the experimental design. Figure S2: Characterization of extracellular vesicles (EVs) isolated from the follicular fluid of either early and late antral follicles. Figure S3: Effectiveness of pre‐IVM in enabling meiotic progression of oocytes to later stages of prophase. Figure S4: Effect of extracellular vesicles (EV) supplementation during pre‐IVM on blastocyst kinetics. Figure S5: Effect of extracellular vesicle (EV) supplementation during pre‐IVM on blastocyst cell number and apoptosis. Figure S6: Effect of extracellular vesicle (EV) supplementation during pre‐IVM on mitochondrial membrane potential (Δψm). [file MRD-92-e70068-s001.docx]

**Extracellular vesicles derived from antral follicles significantly change the transcriptional profile of cumulus cells and oocytes during pre-in vitro maturation in cattle**

1. **Supplementary material**

**
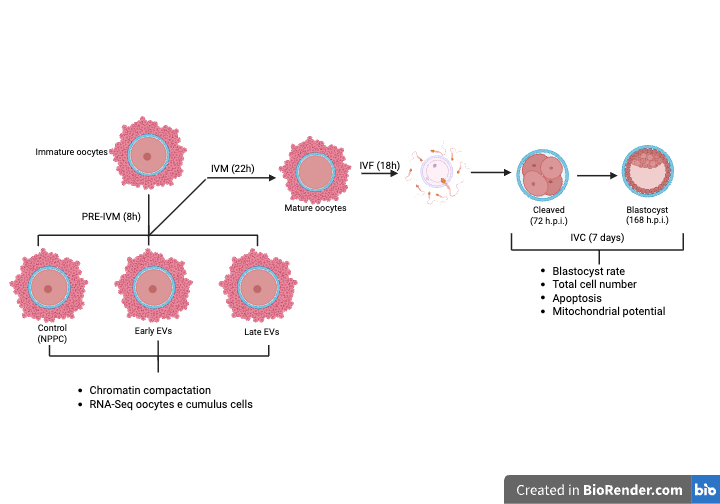
**

- **Blastocyst rate**
- **Total cell number**
- **Apoptosis**
- **Mitochondrial membrane potential**

**Figure S1**. Representative scheme of the experimental design.


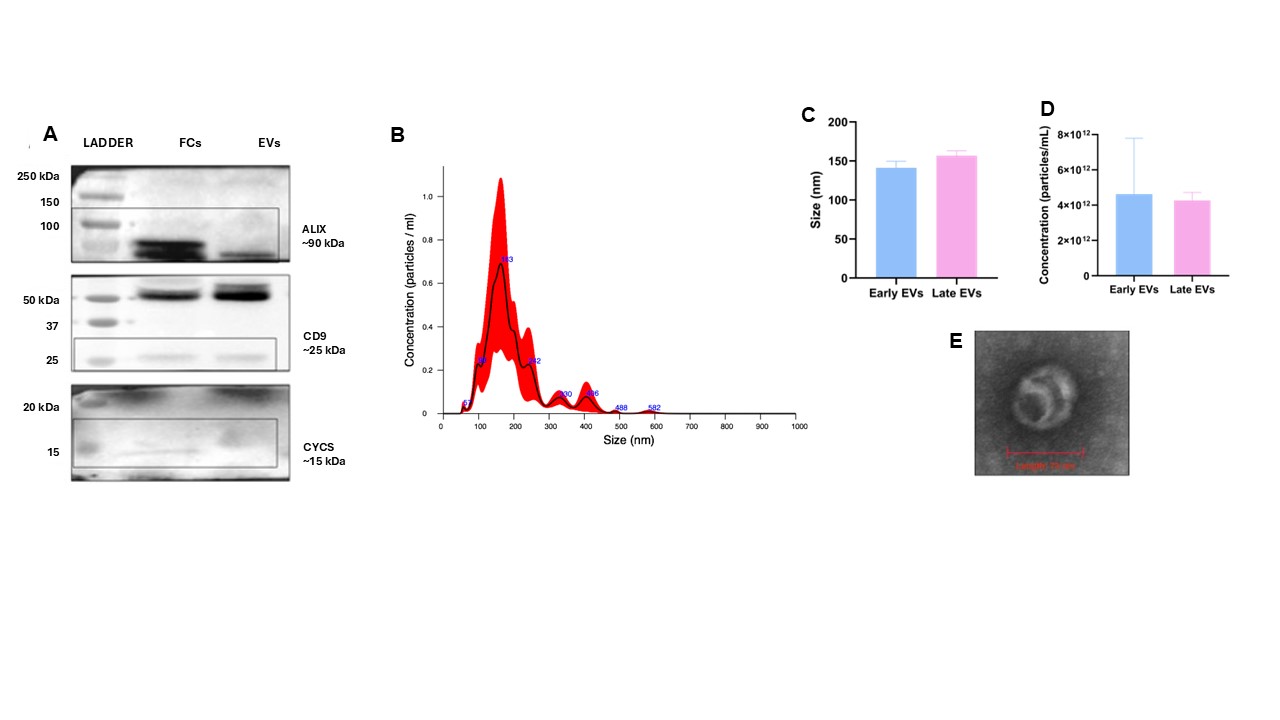


**Figure S2.** Characterization of extracellular vesicles (EVs) isolated from the follicular fluid of either early and late antral follicles. A) Western blotting of proteins used as positive (ALIX and CD9) and negative (CYCS) control in isolated EVs and follicular cells (FCs). B-D) Nanoparticle Tracking Analysis (NTA): B) NTA representative image of particles distribution based on concentration and size; C) Size (nm) and concentration (D; particles/mL) of EVs derived from the follicular fluid (P > 0.05). E) Transmission electron microscopy micrograph showing an EV with its characteristic cup-shaped appearance (bar = 73 nm).


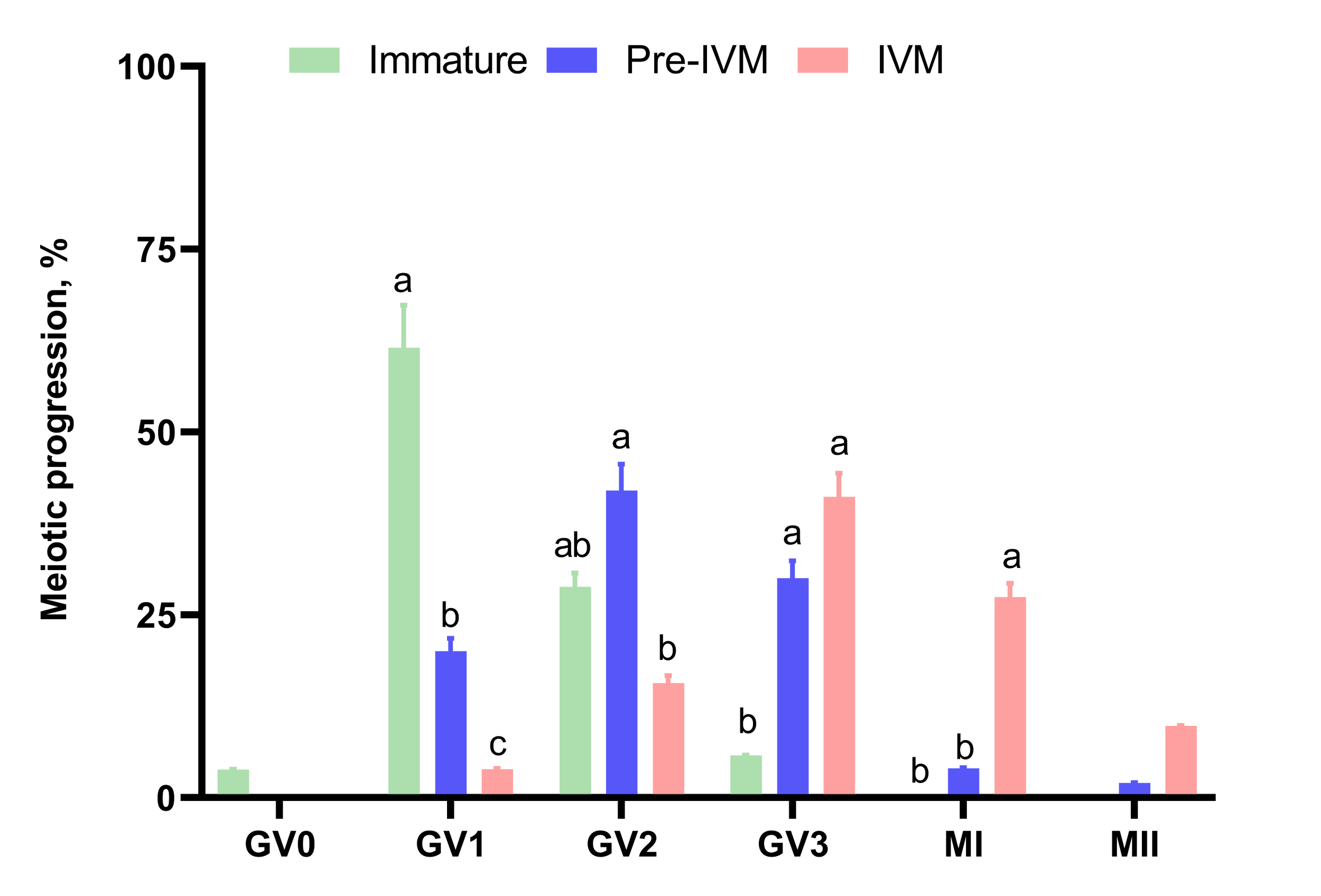


**Figure S3.** **Effectiveness of pre-IVM in enabling meiotic progression of oocytes to later stages of prophase.** COCs were either assessed immediately after harvesting (immature; n = 52), after pre-IVM (n = 51), or after IVM (n = 51). Pre-IVM and IVM were performed for 8 h without supplementation with extracellular vesicles (EVs). GV0, P = 0.3679; GV1, P < 0.0001; GV2, P = 0.0456; GV3, P = 0.0161; MI, P < 0.0001; MII, P = 0.3119. Different letters indicate statistical difference among groups within stage, according to Tukey’s posthoc test (P < 0.05).


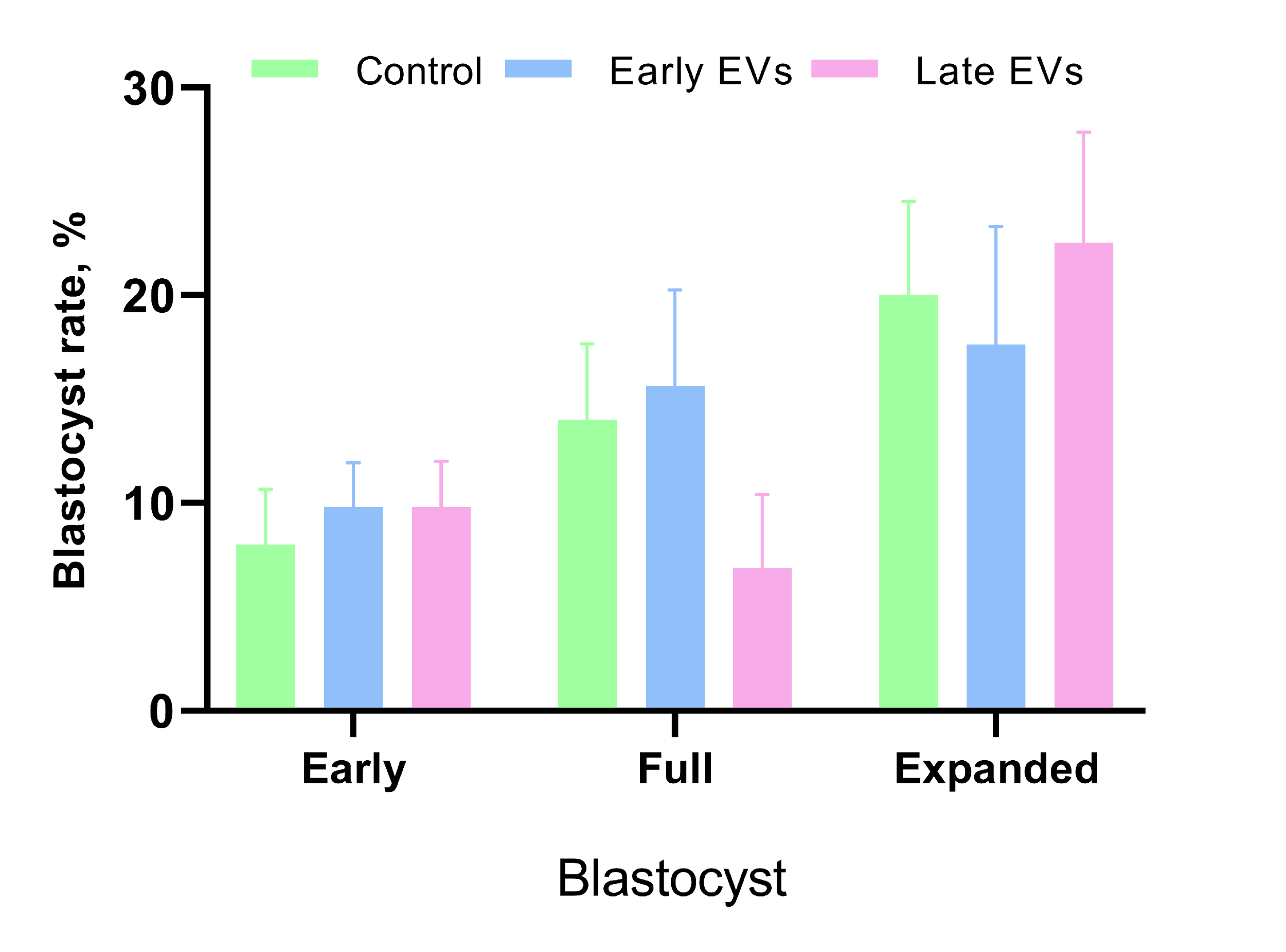


**Figure S4. Effect of extracellular vesicles (EV) supplementation during pre-IVM on blastocyst kinetics.** Pre-IVM was performed in the absence (control; n = 50) or presence of EVs obtained from the follicular fluid of either early (n = 102) or late (n = 102) antral follicles. Blastocysts on day 7 of IVC were classified as early, full and expanded. No statistical difference was found among groups within blastocyst stage. Early blastocyst, P = 0.8573; Blastocyst, P = 0.1924; Expanded Blastocyst, P = 0.7386.


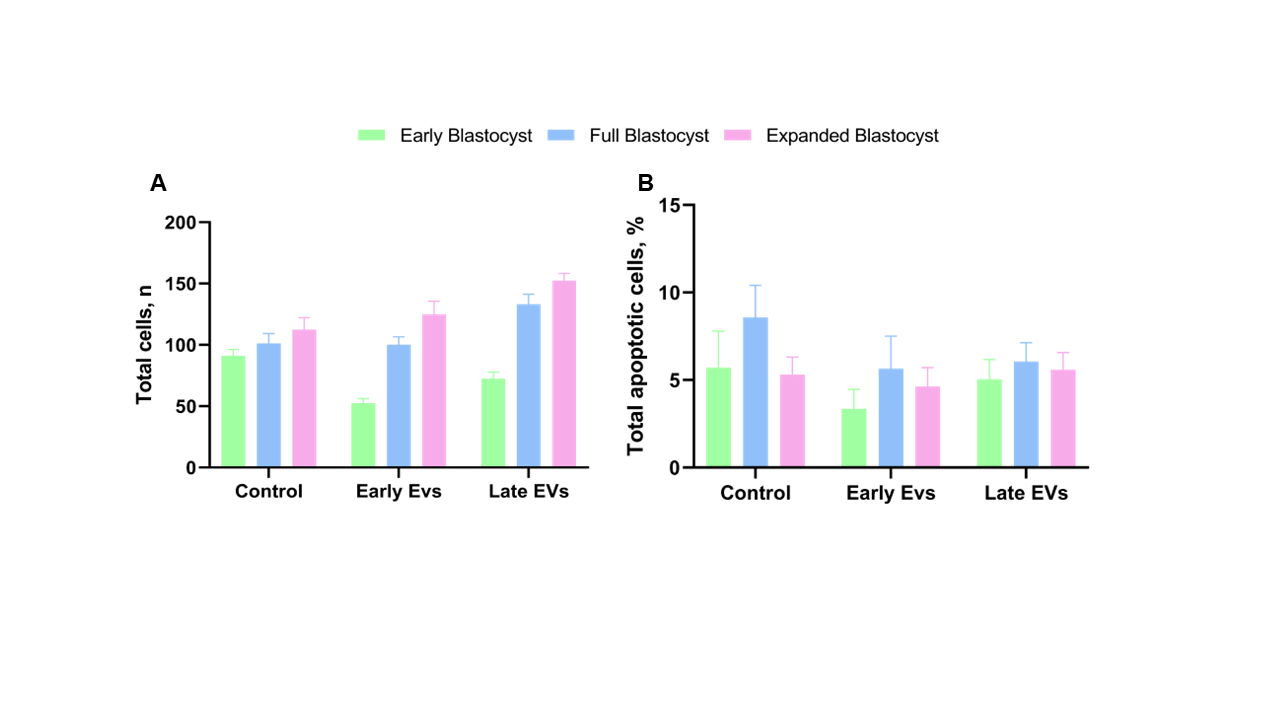


**Figure S5.** **Effect of extracellular vesicle (EV) supplementation during pre-IVM on blastocyst cell number and apoptosis.** Pre-IVM was performed in the absence (control; n = 50) or presence of EVs obtained from the follicular fluid of either early (n = 102) or late (n = 102) antral follicles. A-B) Total cell number (A) and apoptosis rate (B) are depicted on day 7 of IVC according to embryo kinetics, with blastocyst being classified as early, full and expanded. In (A), the following P values were found: P = 0.1424 (experimental group); P < 0.001 (blastocyst stage); P = 0.1275 (experimental group x blastocyst stage). In (B), the following P values were found: P = 0.1713 (experimental group); P = 0.5722 (blastocyst stage); P= 0.6368 (experimental group x blastocyst stage).


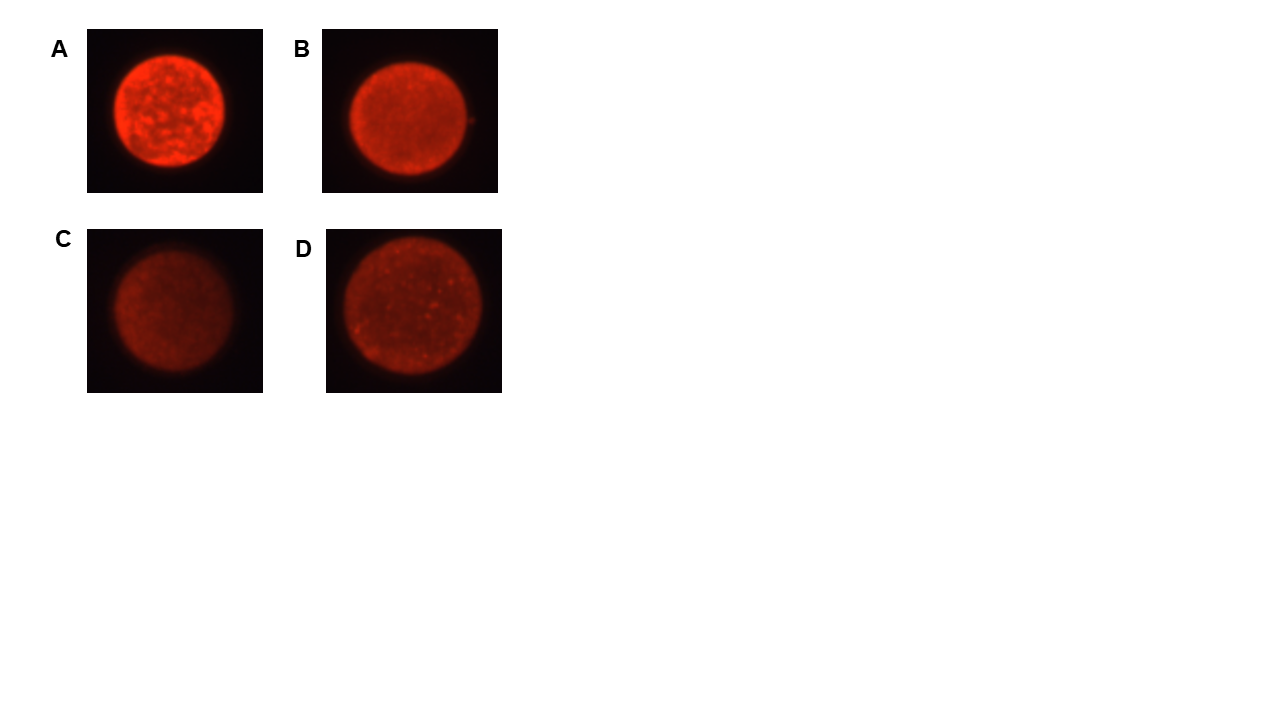


**Figure S6. Effect of extracellular vesicle (EV) supplementation during pre-IVM on mitochondrial membrane potential (Δψm).** Pre-IVM was performed in the absence (control; n = 20) or presence of EVs obtained from the follicular fluid of either early (n = 41) or late (n = 37) antral follicles. Full blastocyst treated with Early EVs (A) and Late EVs (B), showing a higher Δψm, and full blastocyst from the control group showing a lower Δψm (C-D).
