# Supplementary material for: Extracellular Vesicles Derived From Antral Follicles Significantly Change the Transcriptional Profile of Cumulus Cells and Oocytes During Pre‐In Vitro Maturation in Cattle
Source: Mol Reprod Dev. 2025 Nov 24;92(11):e70068. doi: 10.1002/mrd.70068 (PMC12645189; doi:10.1002/mrd.70068)
Supplement: Supplementary file 2 — Table S1: Differentially expressed genes in cumulus cells of Control vs. Early EVs. [file MRD-92-e70068-s002.pdf]

Table S1. Differentially expressed genes in cumulus cells of Control vs. Early EVs.

Gene	baseMean	log2FoldChange	lfcSE	padj
TRABD2B	79.55422854	0.701987769	0.464069	0.00000000000000000000
FAM199X	577.1863086	0.822107965	0.211731	0.00000000000000000000
MVB12B	412.9890914	-1.159312661	0.226347	0.00000000000000000000
F2RL1	330.3815263	0.969139032	0.284885	0.00000000000000000000
GCKR	42.31458774	0.962141157	0.359914	0.00000000000000000000
SLC20A1	162.7658469	0.809615532	0.468337	0.00000000000000000000
LIPE	263.673424	-1.01419696	0.185824	0.00000000000000000000
HOXD8	45.70169953	-0.830117535	0.336287	0.00000000000000000000
NHEDC1	57.67633366	0.970721646	0.352232	0.00000000000000000000
ETFRF1	87.1171121	0.703764046	0.394927	0.00000000000000000000
LOC529196	122.4153082	-1.087040838	0.311865	0.00000000000000000000
TCIM	160.2787394	-1.158719254	0.329205	0.00000000000000000000
PCBD1	3444.196283	0.708232477	0.161456	0.00000000000000000000
ARHGAP33	101.9407052	-0.908915535	0.231963	0.00000000000000000000
BAALC	39.46528312	-0.809928717	0.309418	0.00000000000000000000
TNFAIP8L3	262.5984156	0.940562594	0.280401	0.00000000000000000000
ALPK2	34.74019256	1.232419179	0.390355	0.00000000000000000000
YWHAZ	133.8750558	-0.730268917	0.24667	0.00000000000000000000
PRSS50	4008.645047	0.932160668	0.215531	0.00000000000000000000
BHMT2	351.9992698	0.803419896	0.192584	0.00000000000000000000
IDI1	971.9561395	0.778750635	0.257424	0.00000000000000000000
ANKRD34B	216.9084349	1.088476986	0.349994	0.00000000000000000000
GABBR2	934.8298007	1.057347763	0.267055	0.00000000000000000001
SLC2A6	1485.194714	0.788353632	0.26545	0.00000000000000000001
PRKAG3	98.51438094	-1.579024729	0.326027	0.00000000000000000006
TGFB3	582.4200684	-0.979265149	0.200437	0.00000000000000000017
LMCD1	233.0314034	-0.93653929	0.200843	0.00000000000000000054
RGS22	216.9258833	-0.981986778	0.208973	0.00000000000000000085
C17H4orf33	27.87301407	-1.494673198	0.388305	0.00000000000000000139
ZC3H6	753.6443451	0.704439987	0.177572	0.00000000000000000260
DENND3	91.31352846	0.826625229	0.312356	0.00000000000000001170
P2RX7	95.01156671	-1.212647548	0.306904	0.00000000000000001258

ARHGAP36	7035.134454	0.967500555	0.292442	0.00000000000000001305
ADGRG1	68.27308082	-0.811354304	0.246782	0.00000000000000001531
FAIM2	398.7307477	1.089608357	0.313263	0.00000000000000001774
E2F2	282.6913444	0.902735888	0.265019	0.00000000000000006944
WWC1	94.74649045	-0.740866995	0.248915	0.00000000000000007344
PCDHB11	1326.949261	0.963469526	0.201239	0.00000000000000028227
GRID2IP	288.8066656	0.946844418	0.194829	0.00000000000000065101
FAM84A	48.00561834	0.846473269	0.372839	0.00000000000000094905
TGM2	19.58285069	1.274535629	0.470076	0.00000000000000146062
LOC112446026	278.3650176	1.047627618	0.302624	0.00000000000000164702
KLF9	96.7571363	-0.780888679	0.29364	0.00000000000001289153
LOC107133265	78.05676519	-0.913678732	0.291641	0.00000000000001389228
HIST3H2A	83.22765002	-0.771610795	0.298322	0.00000000000002442057
EVA1A	63.00350477	1.515192873	0.370143	0.00000000000002508749
GPRIN3	148.6471372	-0.779941554	0.213848	0.00000000000003861504
NSUN7	150.7851736	-0.907640298	0.339509	0.00000000000004667730
CES4A	229.8840319	1.203206669	0.287429	0.00000000000007938351
CD9	66.63803531	1.088329588	0.388562	0.00000000000009004208
FZD10	39.76585366	-1.039173884	0.373985	0.00000000000051450300
LPAR1	188.6711097	-0.716961648	0.158703	0.00000000000052826565
MAP2K3	56.81477209	-0.897515318	0.252367	0.00000000000086508002
RBFOX3	42.7256741	-1.226691272	0.435389	0.00000000000093095383
SESTD1	313.9089104	1.490328879	0.454169	0.00000000000116674649
MEI4	5.94715472	1.653767513	1.006721	0.00000000000173348578
CDR2L	78.8721577	-0.844903376	0.367606	0.00000000000174499182
LRRC8C	89.9726903	-0.982903913	0.223767	0.00000000000192623402
RGS4	44.38181765	-0.967734515	0.320678	0.00000000000216163374
NEURL1	17.06189353	-0.99883469	0.510559	0.00000000000218295313
FAM122B	18.03882372	-1.040465092	0.591081	0.00000000000258148199
DCC	45.77786414	1.163747296	0.344545	0.00000000000264865970
LOC104969758	552.0373337	-0.705467856	0.216353	0.00000000000276291201
CA5A	1167.32678	0.784053347	0.207773	0.00000000000399833426
PABPC5	1990.601042	0.8841052	0.259649	0.00000000000771406301
FTCDNL1	14.18199769	1.002091995	0.475336	0.00000000000915762711

LOC505099	42.39101503	-1.607356507	0.39769	0.00000000000984950875
TRO	245.1875914	1.245643287	0.224145	0.00000000001017470443
SPTSSB	137.865277	0.965412665	0.31239	0.00000000001049519512
MAMLD1	498.3179904	-0.797289088	0.232019	0.00000000001103503648
CD200	1677.218896	0.845574418	0.271453	0.00000000001117202960
LOC786352	346.8221652	0.746532085	0.222518	0.00000000001590667459
PIK3AP1	336.2914946	1.126071898	0.289978	0.00000000001821154178
LOC104969173	98.48892086	-0.833166391	0.361478	0.00000000002069694349
RNF133	27.98300211	-1.821760094	0.420591	0.00000000002369367716
TEX2	82.55592546	0.766595287	0.300239	0.00000000003566326004
LOC112449269	249.8969657	1.165281411	0.277308	0.00000000004315921479
TMEM154	179.8204586	0.879488452	0.288777	0.00000000004692874929
PDGFB	142.0170209	0.703905374	0.355584	0.00000000004785172911
EPHA1	271.3294953	-0.943922052	0.45441	0.00000000004930005865
BARD1	31.00435025	-1.42505061	0.430231	0.00000000005436143485
LOC104969384	1194.340279	1.350231465	0.270572	0.00000000010061800618
BOLA-DQA2	671.6541296	0.721341214	0.232427	0.00000000011081602764
OSCP1	170.1729605	1.104064225	0.519812	0.00000000012943799058
LPIN3	81.97860854	-0.778963156	0.28982	0.00000000014279482214
LRRC15	707.2444628	0.842952983	0.224347	0.00000000015570255831
DHRS3	955.8872933	0.757333284	0.290992	0.00000000016062570128
LOC104969934	200.9548399	-0.706487417	0.269735	0.00000000016160350125
MARC2	56.74763854	-0.914167305	0.297834	0.00000000023555161455
ART4	2043.458583	-0.735778843	0.207233	0.00000000028882356901
EPHX1	18.64452927	-0.754314586	0.481681	0.00000000032049854483
BCO2	65.14866882	0.778708989	0.368833	0.00000000033357952908
TLR2	57.64083601	-1.026155498	0.28463	0.00000000037049770104
BDH1	38.46608854	-1.021505678	0.329922	0.00000000040254217685
FMNL1	81.03714683	1.219950298	0.635071	0.00000000043115919321
ST14	36.05397541	0.76878528	0.345965	0.00000000048253815940
MAP1LC3C	13.81443348	-1.600895535	0.531885	0.00000000054353746147
CFAP46	246.8601372	0.838598284	0.324563	0.00000000055427491952
LSMEM1	379.7734692	0.892696541	0.222214	0.00000000059841125069
OMP	26.73518662	0.738247367	0.34539	0.00000000071131786327

PTHLH	19.36394087	0.864732206	0.449398	0.00000000072128295006
ZSWIM3	4.86021278	0.97777293	0.9833	0.00000000073025772753
GLRX5	60.75403139	-1.456378469	0.459416	0.00000000078228642061
LOC112445660	1162.916436	0.83379156	0.287398	0.00000000079471148512
ABCA1	191.2718619	0.798697104	0.340532	0.00000000083909673516
SS18L1	293.8537044	-0.769305069	0.240269	0.00000000095017705182
LOC531747	45.6141069	-0.710511937	0.364882	0.00000000106787011509
PPARGC1A	381.4835381	0.760788794	0.229178	0.00000000145587307595
WLS	60.02937213	-1.081812693	0.320872	0.00000000154103874960
KCNH8	45.63628761	-1.579219667	0.373161	0.00000000154791264801
TRIB1	58.53737581	1.271420564	0.421787	0.00000000159368119306
LOC101904698	18.50687449	-0.907689378	0.590146	0.00000000196705068677
FRK	1599.127712	0.763410593	0.239935	0.00000000250204877068
C19H17orf100	74.19196941	1.056623812	0.283699	0.00000000393282902630
ZNF169	91.83524429	-0.860676464	0.38139	0.00000000495563938791
LOC112441682	323.7601731	0.780467177	0.311785	0.00000000657228880518
PTX3	463.9173398	0.807727617	0.216514	0.00000000847338467252
NET1	3683.4699	0.891822725	0.29117	0.00000000856768310051
LOC101905905	17.01167685	-0.736310171	0.481638	0.00000000939060322926
DAPP1	353.5044515	0.74052389	0.238227	0.00000000959576616806
LOC104973105	2128.743308	0.973149683	0.224292	0.00000001103250829824
IYD	98.65934312	0.744399263	0.334961	0.00000001439576869398
PCNT	22.15605515	-0.870235314	0.475822	0.00000001610408819686
RPS28	7.211433571	1.049562906	0.882932	0.00000001718355391837
CD24	89.65168136	-0.974224001	0.307745	0.00000001810046229253
OAS1Z	1575.297639	0.730535395	0.200461	0.00000002479173770513
PHF24	6.915497181	-1.684159389	0.884744	0.00000002727722217655
MAB21L2	70.78460417	0.728472645	0.255998	0.00000002845619200552
ANK1	67.56589665	-0.997061589	0.32197	0.00000003776096492506
LOC101908577	104.8890159	1.522489819	0.402043	0.00000004075028991112
PSD4	42.15736818	1.349379936	0.46724	0.00000004136856736688
AMIGO2	6.682725532	1.012003567	0.792119	0.00000004936974404240
DQX1	34.18940963	-0.99575063	0.388139	0.00000005008727294563
ABCC9	235.6977716	-0.911272983	0.489362	0.00000005077936691572

S100A4	61.22957745	1.438012326	0.451812	0.00000005173524048866
CLEC14A	45.05174766	1.000261689	0.349223	0.00000005512975414080
LOC781624	42.78954987	-0.735287614	0.363291	0.00000009054663922659
LOC112445823	288.9744665	-1.003745552	0.272616	0.00000009777630990651
LOC515333	10.2642341	0.828459382	0.684677	0.00000010714659077648
TUBB2A	42.67075432	0.765679986	0.261841	0.00000012315664721438
NEDD4L	55.7793738	-0.77000547	0.299054	0.00000012613176467658
PPP1R1A	29.16833948	-1.193758919	0.501689	0.00000012724086508184
KCNJ8	1080.244632	0.85968002	0.294345	0.00000013139253171332
PPP4R1	918.7905194	-1.066406278	0.221182	0.00000013970591625102
TMTC2	7.77160684	-1.053184688	0.65217	0.00000014415006146539
SDCBP	7.524291248	-2.394478006	0.68607	0.00000015792408335052
ABCA4	111.2310988	1.101011126	0.29223	0.00000016464763725239
SEMA5B	13.66220084	0.821268924	0.44386	0.00000016734410162388
TM4SF1	71.04628237	-0.712922936	0.254655	0.00000016843749456997
INPP5F	214.0044252	-1.065218355	0.310817	0.00000019501163772067
CYR61	164.894552	-0.85916855	0.183222	0.00000020074357157442
RIPK2	90.01960642	1.010270206	0.37252	0.00000021839689716353
TNFRSF18	26.17789595	-0.799286493	0.445623	0.00000023196504212366
TCFL5	9.671040363	-1.390576255	0.52818	0.00000023711704602123
RIMBP2	11.77432229	-1.533295041	0.625302	0.00000024545829978312
ADAMTS2	91.27366451	1.018211459	0.375265	0.00000024896049225982
ZNF541	132.3487194	1.273165868	0.359693	0.00000025142187353070
C2CD4B	246.5488551	0.745353558	0.219187	0.00000026412432229484
LCA5	71.59506305	0.727267938	0.289753	0.00000026429811336536
CTH	9.357292411	1.187706627	0.505289	0.00000026514134670685
RILPL1	54.42385267	1.245487516	0.462553	0.00000027546957177048
HMGCR	26.06652385	-0.949272362	0.419723	0.00000028128181403766
CYP17A1	38.26573631	-0.742606937	0.293177	0.00000031933631734151
GLIS1	7.228372315	1.481074696	0.502702	0.00000034570449895488
LOC112441810	4.669135861	1.034534718	0.81998	0.00000038166968708656
LOC112444744	61.46035241	-1.37558728	0.271467	0.00000038373377088375
IL3RA	17.25725003	-1.539950669	0.592596	0.00000048126763523188
IARS	10.31438824	1.17506897	0.62868	0.00000048439108802876

TAB3	43.63418297	0.847554555	0.326959	0.00000048946777895904
TGM5	18.25566156	0.756482008	0.422523	0.00000051399360975825
LOC511498	25.81054064	-0.750721384	0.461502	0.00000053561989083787
ASTE1	61.16252653	-0.844644677	0.316318	0.00000053677041738120
SYTL5	2247.033965	0.715678361	0.247104	0.00000055647402564459
CNTNAP4	38.32496514	0.765848521	0.250117	0.00000057038973135583
PDE11A	58.84955073	0.941845824	0.34012	0.00000057583544234652
OR11H6	51.70724723	0.895906959	0.38858	0.00000059007679642063
ELAVL2	197.193067	0.868738479	0.228	0.00000078248937239832
ALPL	324.2042727	-1.045901612	0.28513	0.00000078773886894945
LGR5	45.88348659	-0.789853067	0.362069	0.00000081333875955334
WARS	61.24299254	0.734161059	0.459845	0.00000081490660410805
FUT6	18.59830802	-0.856856027	0.52409	0.00000082757232845301
RNF144B	53.62620128	0.898292409	0.321538	0.00000096090858861134
FAT2	9.933168915	-1.136870532	0.586773	0.00000104481032280641
TMEM144	4.98737255	-4.276972578	1.190605	0.00000104481032280641
SOGA1	81.9736366	-0.824562229	0.294748	0.00000108018725450206
ZFP36	9.469249265	-0.814828236	0.499765	0.00000108018725450206
LOC101904722	26.91585496	-0.701026589	0.395223	0.00000108168308946156
ADAM19	36.71452652	-0.87121682	0.332786	0.00000125672558500792
IKBKE	20.09509569	-1.14303341	0.538675	0.00000131371897724301
INTS2	335.2179602	0.798406221	0.290459	0.00000132879041221074
SLC38A5	25.74391791	-1.005917021	0.341487	0.00000136552245992213
TMEM92	134.2954528	0.776545047	0.340482	0.00000142606990630780
CD274	18.53524874	-0.830234839	0.435285	0.00000200697541611456
TMEM200A	7.335401859	-1.768296464	0.699638	0.00000202921062450779
NFIB	40.78281032	0.702813498	0.270915	0.00000208597767120961
SRGAP3	191.423611	-0.862097089	0.313098	0.00000214641048844297
ZMYND12	14.70140753	0.989143325	0.509766	0.00000225016323262513
LOC787102	6.936053888	-2.913237921	0.900834	0.00000225705133393741
APOBEC1	31.62673109	1.097100222	0.402059	0.00000254890720226678
MARCH3	319.7429786	-0.717293029	0.243159	0.00000258567825895079
TSHZ3	48.26186429	-1.009761588	0.400483	0.00000262538545211217
LOC101903056	22.16039933	-1.132686951	0.500312	0.00000275844624174323

CEP112	1861.603333	0.83262831	0.20682	0.00000309978542218494
ANKRD9	72.55013271	-0.785755439	0.324136	0.00000315918635110422
PLPPR1	71.64159149	1.063984166	0.320372	0.00000454360557787261
LOC524391	258.7385552	-0.704691662	0.217195	0.00000495956824664179
METRNL	32.8112529	-1.061175567	0.406978	0.00000511538242546358
LOC112444626	188.8741672	0.749362849	0.259666	0.00000519185323763826
BEND6	79.04214033	0.776503073	0.263266	0.00000533390893332170
RAB38	7.391006023	-1.055732711	0.814443	0.00000539094255746429
LOC101905571	8.405523468	-1.806019407	0.730725	0.00000546576759790986
LOC112444960	539.9168527	0.798120698	0.22635	0.00000575047905198503
AMPH	388.3030165	1.018035318	0.397865	0.00000603963680024067
LOC104971236	578.0764573	0.808479619	0.282807	0.00000616266660984715
RUSC2	228.783424	1.315078568	0.347282	0.00000619793159511721
TMEM232	1352.580423	0.873568517	0.2678	0.00000630180117405094
GPR135	352.2744947	-1.052113852	0.267966	0.00000638867829078496
LOC101907189	62.49227628	0.986402035	0.396208	0.00000685022286385223
CNOT3	15.55335587	-1.070366307	0.57411	0.00000686646043689243
CLMN	164.9088448	-0.72491183	0.330193	0.00000695147601852372
BOLA-DQA5	9.611750934	0.964623563	0.661635	0.00000708657679136317
LOC101906330	6.326412473	-1.422648133	0.808066	0.00000757366750827222
LOC112449540	308.7364499	0.783694089	0.281778	0.00000757366750827222
FAM53B	19.75718115	-0.939595338	0.477744	0.00000791455004953347
LOC112441664	376.0019643	-0.975370066	0.250574	0.00000798095597236613
LOC101903639	26.28421166	1.227763733	0.405665	0.00000829902914385111
LOC101903600	109.2489389	-0.775904395	0.205833	0.00000888857042477394
TRGC4	308.9882567	0.836265706	0.304516	0.00000917615974254085
FAM131C	85.38292962	0.768903227	0.297273	0.00000988073086574364
KRT6A	30.72882083	1.868839204	0.453787	0.00001042831886429000
VWA7	36.35535343	-0.866750926	0.322481	0.00001059691409934770
NCF1	14.32894785	-1.050620548	0.463567	0.00001091587115687710
GP9	3792.454331	0.718155262	0.256368	0.00001125794825536460
RPS21	38085.85167	0.976049821	0.225384	0.00001193831164457700
IFIT2	15.57265846	-0.726034451	0.448046	0.00001281226988976880
ACTC1	91.66753814	0.95227742	0.36246	0.00001309993936096190

CKB	697.1972792	0.70869434	0.228626	0.00001319966635010210
CADM2	1765.772306	0.752418499	0.222942	0.00001350751365285520
RPS6KA1	38.57604874	-0.875710149	0.367326	0.00001379594461154340
LOC104968656	1810.13935	0.81747426	0.253299	0.00001459456080155800
TRPC5	18.83888971	-1.649298996	0.654667	0.00001572073028273790
POLN	157.4135728	-0.944063309	0.304732	0.00001762028970862440
PTP4A3	29.35330762	0.798911497	0.362254	0.00001820201071667920
FAM19A3	2667.105287	0.704426477	0.266589	0.00001904553574602100
GRIN2D	4.313652406	-0.848110954	0.882698	0.00001993761734633920
SGIP1	77.54560395	-0.751278717	0.426367	0.00002027291267324920
ANKRD37	42.25502234	-0.90293243	0.315343	0.00002047947428305520
STON2	53.13280487	-0.841044088	0.270293	0.00002191751355410180
TRIB3	25.71568722	0.893145291	0.376403	0.00002194046303148290
MAP6	5.921456936	0.92167344	0.653045	0.00002258889267335400
MIS18BP1	6.427470623	-3.205116925	1.065025	0.00002486772506039880
SPOCK1	20.2484236	0.954678055	0.403479	0.00002497198002672710
LOC112449267	61.91212529	1.462884606	0.42538	0.00002600912830877530
SH3YL1	90.93849715	0.882729523	0.308752	0.00002702289323843840
PDCD1	15.21277008	-0.994044471	0.487609	0.00002721934143563670
MAPKAPK3	12.70113712	-1.477788727	0.67158	0.00002911398887977930
TLR5	10.55679297	-1.032731544	0.536816	0.00003187577534027660
LY6E	15.24885782	0.923603643	0.557219	0.00003214857244669410
LOC101909003	3695.014812	-0.836987911	0.186494	0.00003651122688171500
KCNE5	924.7509306	0.704485348	0.273645	0.00003655355179969380
HSD3B1	243.736946	-0.825098789	0.326712	0.00003904991371241450
RAB11A	72.90225453	0.887642817	0.336242	0.00004098625382116600
MSN	67.23364765	-0.938718291	0.350312	0.00004211314560508810
COL3A1	4073.842175	-0.775736319	0.158738	0.00004457555012305320
IL7R	15.52414079	-0.739009746	0.424107	0.00004458171591038550
LOC104973829	12.1806795	0.908525176	0.519379	0.00004629051034754560
KRT76	460.7299415	-0.958520786	0.261521	0.00004651897990959480
LOC104969334	42.91222117	1.132576891	0.429673	0.00004803236649417370
SYT2	1713.429454	0.8940646	0.254328	0.00004864274355560970
CSGALNACT1	11.54711313	-1.134619619	0.526311	0.00005003648126477240

TM4SF20	356.8546044	1.019525847	0.285592	0.00005185650571383710
ITPKA	17.84867047	-0.852805196	0.545743	0.00005190579964778680
LOC508879	28.81334056	0.886955171	0.458823	0.00005199806582788860
RASA2	15.81196677	-1.107877736	0.48454	0.00005232132857047760
LOC101905992	18.54674122	-1.477407775	0.405299	0.00005245148451452600
PKIB	5.113240811	-1.734651834	0.855531	0.00005352488422640720
ALCAM	32.90724405	-1.111955529	0.574307	0.00005355605472942950
RASGEF1A	8.695476516	-0.856639015	0.649217	0.00005381926530348920
LOC112449308	9.440460304	0.826306808	0.627595	0.00005490339170032870
ULK2	19.58836144	-1.050494502	0.531477	0.00005771450638436340
LOC112443897	26.01276889	-1.357280987	0.507387	0.00005837083939653990
ALOX12E	171.2754428	1.185429158	0.268881	0.00006259921666678660
LDLR	68.1937414	-0.713712922	0.336715	0.00006601440334940150
TIGD3	115.3002552	-0.802592908	0.221775	0.00007080141662888090
REG4	19.1421495	-0.872662468	0.43098	0.00007170609870617950
CBR1	517.9590904	-0.906796934	0.253918	0.00007255204491688270
LOC112449624	86.43354757	1.089006093	0.390193	0.00008064551639046740
IL6ST	62.70152435	-0.943588485	0.34941	0.00008094341333153320
CCDC168	59.66862813	1.240089999	0.360213	0.00008308550036565510
CHI3L2	291.7776393	0.890293295	0.290997	0.00008628856573342920
ZACN	9.926758486	1.336239282	0.444807	0.00008979772303760620
SMTN	11.29836228	-1.790657656	0.588514	0.00009016775073448540
CCDC184	9.246030178	-0.807532626	0.526064	0.00009534679628764450
SELP	7.168781175	0.833255784	0.538775	0.00009623997349861100
LLGL2	14.91338349	-0.828551158	0.493813	0.00009967068212671940
RN7SL1	40.6449052	-0.706470832	0.355323	0.00010523746560642200
DOT1L	12.90719049	0.725850319	0.40109	0.00010542440228806700
HOXD9	49.97362249	0.812734911	0.341598	0.00010771401089947100
LOC101907707	17.35975746	-1.095516023	0.451518	0.00011032408689049200
SMIM17	8.93024265	-1.66531748	0.809518	0.00011039492077510100
LOC104968522	15.07241776	-0.806932581	0.457633	0.00011462683484890200
LURAP1L	23.4729964	0.890001993	0.337746	0.00011462983261756500
HRCT1	1008.982658	0.792916739	0.732878	0.00011487615505112100
AFMID	809.966678	0.869126636	0.299137	0.00011511630417934000

TMEM54	15.97398088	-0.819589787	0.449549	0.00011579589010407900
LOC112449612	9.280225974	-3.929166962	0.931529	0.00011592083938912200
GUCY2C	14.42371338	1.735068207	0.592254	0.00011916609016961100
GALNT15	9.072155718	-0.915165797	0.645249	0.00013014876090128500
PCED1B	17.28650727	0.999258948	0.48739	0.00013106686551736600
DNAJA4	45.79745711	-0.916550441	0.356259	0.00013835432671628800
KIAA0754	7.880848964	-0.939743682	0.767534	0.00014443587292028700
NETO1	49.15123049	1.449188491	0.380819	0.00015278638885056200
GPR156	130.9682231	1.547028515	0.352373	0.00015386293813434200
EXO1	1741.44557	0.9299059	0.283362	0.00015987315253636300
PRKCB	5.66795169	1.092690447	0.796319	0.00016511954800186800
FABP5	52.97248384	-1.068864149	0.361	0.00016751517338337700
GRIN1	7.747495822	1.416358618	0.60991	0.00017245149801333800
LRRIQ1	6.216032077	-0.87666104	0.795886	0.00017538567288718700
LOC104973098	5.321992175	-1.576074899	1.030585	0.00017811346116556100
NUP205	6.612056032	-1.757545982	0.771012	0.00017852683621833400
TNFRSF8	31.78546644	-0.761904261	0.446419	0.00017929990910133300
THEM6	20.3627129	-0.965943763	0.427022	0.00018067012391077800
TUBB2B	34.96737813	-1.283475816	0.378586	0.00018418534575612800
LOC101905687	14.94536237	-1.013223904	0.468048	0.00018526006659008900
ZKSCAN1	13.14398032	-0.815214102	0.664637	0.00018890001345866900
PVR	58.24967943	1.005517757	0.357818	0.00018942818612660000
LOC112444279	57.6601838	1.357562952	0.566892	0.00019275793613655600
FUT8	146.5257184	1.110464584	0.341945	0.00019615022584818100
CFTR	4.882997424	1.06377642	0.882599	0.00021820637659358000
DSC2	47.65364804	0.807920385	0.360726	0.00021961109046051000
GJB3	21.78826811	-1.492653098	0.465293	0.00022568256170857800
CPA3	95.49803256	0.904177137	0.273921	0.00023623009746537200
PLEK	6.024840153	2.445622877	0.919409	0.00023675172872515100
LOC784052	2218.855929	-0.985012862	0.216595	0.00023717706371093600
LOC101907782	115.5216102	0.80743896	0.34195	0.00023758892877417700
SMAD6	325.2234933	0.777765208	0.210126	0.00023938038837134700
TMEM18	15.2398747	-0.705153625	0.548922	0.00024343820594651800
ETFBKMT	14.09400131	-1.044222879	0.623427	0.00027958360410623200

UBE3D	15.35578739	-1.563827661	0.503509	0.00028099698198438500
NGF	25.42732998	-1.120357838	0.533848	0.00029744127288262200
YEATS4	5.266309744	-2.56960992	1.091203	0.00029819444047757800
LOC101904680	59.96866059	-0.976995648	0.339757	0.00029928678696673900
GJA5	39.5882744	-0.871855584	0.377931	0.00029964566866809000
SLC24A3	12.59130463	-1.152146319	0.533532	0.00030776360488869100
LOC112446345	94.83509687	-1.259600585	0.338848	0.00031606848524812300
TEX9	63.87033103	1.02416442	0.3683	0.00031804339675725400
LOC104970453	140.4773171	1.321756929	0.371252	0.00033488795081573200
BSN	29.27353372	0.804688288	0.379862	0.00033937812689618500
ARL13A	415.2026908	0.897096454	0.240172	0.00037179910198937400
CTGF	151.159941	1.345274126	0.321377	0.00037565828278101700
SSLP1	79.40231511	-0.813371219	0.309555	0.00037939478328030100
LOC112449175	841.536716	0.976378812	0.298606	0.00038816788670035900
LOC104972797	482.3912593	1.267916012	0.290851	0.00038871696351784600
TGFB2	113.6141377	-0.831186387	0.343934	0.00039158544844796300
MICAL2	76.96584454	1.279763986	0.424603	0.00039438808713022600
PROCA1	247.2626183	0.737453193	0.213928	0.00039876747853720400
IDNK	123.6513885	-1.272577806	0.603523	0.00039932489559959300
AZGP1	172.0997095	0.72351322	0.279639	0.00042075632995250400
LOC104969457	78.58493233	-0.713233519	0.234903	0.00042904294746322700
RASL11A	369.9120559	-0.819280755	0.21621	0.00043679874810363100
PDE7B	29.15735749	-0.803288939	0.407397	0.00044279748115922000
NTSR2	31.66451724	1.696400034	0.512837	0.00047461247647417300
FERMT1	7.67278718	0.758417064	0.732037	0.00048015871259411600
KCNT2	30.64720664	0.72846383	0.448544	0.00048093861512329800
KCNJ16	45.88110348	-1.20147343	0.443912	0.00049626583754916700
ACSM3	54.2637424	-0.930250321	0.32495	0.00052077501244740000
NDNF	29.91593857	1.830861517	0.456003	0.00053096924480653500
BACH1	8.408217341	-0.790124732	0.63125	0.00055481271318330300
EFNB2	88.68119397	0.794780956	0.289667	0.00056171819782873200
TMEM229B	17.46074183	-0.935532156	0.422387	0.00056459817928823200
LOC107133137	6.177811099	-1.235605378	0.770767	0.00057442728806995100
COL5A2	6.574460546	-0.933152349	0.733278	0.00059681961873117900

STK17A	124.5598763	1.291771314	0.354234	0.00070785965419593800
LOC101907322	41.41367037	-0.737735478	0.353257	0.00072081573339121000
LOC782682	6.935735832	-1.591511859	0.728915	0.00072195956596875800
PPP1R42	9.256904282	0.960910702	0.593276	0.00078459276067907900
GPR18	58.37607587	-0.730085652	0.36477	0.00078599143627292700
PHACTR1	163.4968554	-1.292283613	0.277326	0.00078735162346045200
GPC5	11.42536674	1.945672595	0.56317	0.00079160573743731500
CDKL2	22.94005857	0.750157707	0.475892	0.00081331778165602200
RETREG1	22.24888408	-1.126418891	0.517156	0.00085178359804895500
MX2	190.0407865	0.811491844	0.332436	0.00085912152607012900
DNAJB11	71.96583639	-0.864113794	0.427757	0.00087649438855794500
TJP2	3444.936751	0.82253063	0.244545	0.00088503131351136600
PLEKHF1	8.615046544	-0.813271601	0.686397	0.00089204806462236400
GREM1	88.22771437	-1.693064472	0.533303	0.00089969738278281900
HMGCS1	183.5057046	-0.705713089	0.213586	0.00090436143077154400
SDC1	307.8354934	0.745993615	0.201486	0.00091190622009211500
TMEM95	91.01281629	-0.786678479	0.285839	0.00096445910076476900
LOC104969611	5.577854999	1.298734237	0.82086	0.00097897965237760300
FAM189A2	18.86849155	-1.374753512	0.508675	0.00102681557684440000
PASD1	5.244871578	-1.579725571	0.968752	0.00102685017478824000
MATN3	5.42811195	1.027607407	0.810257	0.00106977376700748000
ST8SIA4	3.348813705	1.372022718	1.165517	0.00111232573198718000
STK10	29.86919368	-1.589843764	0.496181	0.00111404763362996000
ZNF157	1754.679833	0.911536069	0.26748	0.00113524124461404000
DOK6	6.719145013	-1.235070631	0.748993	0.00115285027684054000
PTPRB	17.24499996	-0.771516949	0.549329	0.00117327614121963000
RHPN1	6.976150541	-1.521402998	0.7815	0.00119754924345463000
C25H16orf45	41.29856106	-0.923277729	0.447051	0.00119999319581152000
CNTF	100.9920474	-0.866540186	0.378602	0.00123347678706581000
LOC104973860	671.7617474	0.750457483	0.283785	0.00124279491234707000
NDST3	146.1844336	0.800383577	0.256594	0.00125445502377511000
PRR11	24.50213115	-1.104014557	0.395309	0.00125531993280427000
TMEM110	10.13715687	-1.445162146	0.70371	0.00128591018777889000
UTP20	10.39564492	1.049569549	0.5338	0.00130920307899709000

LOC112444614	327.5313861	0.834958551	0.457834	0.00132421866712268000
SLC7A11	3939.569371	0.761369599	0.242418	0.00136449842818964000
LOC104969435	9.998927199	1.517623737	0.459821	0.00136508049262451000
NFIA	8.174423045	-0.777487787	0.72199	0.00144507398706350000
RND3	106.0059111	0.811944734	0.255533	0.00146739830104284000
YDJC	10.69523247	-1.803211993	0.663182	0.00149718622538319000
LOC512005	65.14811279	0.782037265	0.25701	0.00150669013023283000
IQUB	11.57534299	1.65701065	0.69687	0.00155844009997201000
GRASP	3.436084031	-2.942606984	1.16557	0.00161949323528868000
LAP3	7.377986466	-1.176267357	0.680466	0.00166756668470072000
SPAG16	58.86598352	-1.06927853	0.342621	0.00167342613357907000
CACNG4	1479.9827	0.71058403	0.208828	0.00170331211677549000
DCAF16	7.524960376	0.883941129	0.758913	0.00170894405998747000
RBM46	13.0758738	1.266711341	0.620194	0.00177239282334785000
ATXN2L	10.36540328	-0.966955643	0.572709	0.00186471187227561000
JUND	6.64141707	-1.921094439	0.718783	0.00190287354576838000
GSN	51.36311882	1.17832118	0.615069	0.00191006286449613000
YJEFN3	47.58825611	-0.871616311	0.267679	0.00193932059305740000
NECTIN4	32.51745078	-0.74600007	0.390341	0.00193942901078563000
NEIL1	32.99651683	-0.765992317	0.339318	0.00193950895250848000
INSIG1	16.48701874	-0.86347704	0.453272	0.00209977259830402000
TNFAIP3	671.8189423	-0.703987395	0.260996	0.00209977259830402000
UBASH3B	64.19474953	-1.042276686	0.259514	0.00210903608394367000
AHR	38.92585521	1.381168289	0.667466	0.00211116085596779000
NTN1	32.36900418	1.128493267	0.451959	0.00212939189262327000
ZBED9	43.849873	1.11184379	0.708811	0.00213027362601720000
PDGFRA	356.1727489	0.92115764	0.243218	0.00216905516402111000
COL6A5	2005.938733	0.820840499	0.306568	0.00226773304587295000
FNDC10	4.418705723	-1.713987917	0.975845	0.00235974056332351000
LOC112449271	55.21647292	-0.851719136	0.326941	0.00238266667303352000
BDNF	7.280192203	-0.905356582	0.674782	0.00242757411551272000
LOC101906850	42.08410813	-1.225753051	0.348716	0.00243672264585760000
GNAI1	10.59953173	1.481216719	0.661388	0.00246055659417909000
CCDC150	8.196740311	-1.346725443	0.663422	0.00247082968544720000

HYDIN	32.12208344	0.934283064	0.330589	0.00256657851424989000
OGDHL	8.161065151	1.21635716	0.524701	0.00264097598546016000
CXCR6	6.876178328	-1.536889025	0.816748	0.00264539894506111000
ZNF581	32.15464496	-1.201004616	0.384483	0.00267978123139994000
CILP	18.82317291	0.748264032	0.319884	0.00273039070993360000
PIK3IP1	11.08248286	0.921187444	0.686143	0.00281223983726337000
KRT6B	3.819131267	-2.074029463	0.922954	0.00286751619532928000
TMEM130	158.6560474	-1.25538206	0.505512	0.00287033085918308000
HAS2	5.474123178	1.215189873	0.775797	0.00287411138491591000
LOC618212	26.17660741	-0.840644903	0.333558	0.00288347832894520000
IER2	9.125680991	-0.726240192	0.602228	0.00298215526979477000
LOC101903857	1008.254652	0.729042558	0.184422	0.00301636689505714000
KCNMB4	52.80870989	0.750100423	0.400381	0.00302421005219873000
ZBTB8A	21.46241378	-1.166184144	0.368036	0.00303386752202636000
BMP4	10.41495083	-0.861705998	0.638052	0.00303630546777630000
FJX1	13.48316827	1.391021766	1.864701	0.00307038605898572000
COL14A1	3.422613854	1.220978375	0.948279	0.00317867078609928000
CSF2RA	7.597173528	1.12607312	0.571834	0.00318854202770319000
IL2RB	6.817500743	-1.346273021	0.824272	0.00319984947780876000
ATP13A3	514.3264402	0.979193046	0.269843	0.00321823826916335000
DAB1	16.82814768	-0.732409833	0.423292	0.00328103642735050000
LOC101909455	23.20051119	-0.99286203	0.399532	0.00328403542287983000
CBS	20.74160349	-0.733586536	1.203703	0.00329804808534630000
LOC104971069	17.04743905	-0.772784506	0.554748	0.00333519881372002000
SEZ6L	9.742321738	0.900031161	0.418955	0.00338417946299546000
ISG12(B)	6.119160317	2.095541465	0.769519	0.00341071179569641000
RDM1	50.78825375	0.764579596	0.378982	0.00343383448077625000
CNIH3	9.199027232	1.097767804	0.590634	0.00355391915696433000
NMUR2	19.76941379	2.290440505	0.610113	0.00358262057611926000
PDP1	808.3840153	-0.797680668	0.244325	0.00359350081357559000
GRM5	249.1133678	1.070394615	0.338951	0.00360338825721563000
DAB2	6.151298227	-1.104363046	0.755569	0.00361363762038818000
NSDHL	36.31755677	-0.866130828	0.340997	0.00363077901492818000
SLC6A13	30.73380703	-1.257948505	0.806236	0.00363391354190189000

FBLIM1	186.2247115	-0.868693259	0.219866	0.00369356950292379000
GABRA3	42.54379616	-0.825168586	0.372791	0.00370977038115464000
TFPI2	70.14591265	-0.931860774	0.281282	0.00379580383690086000
ANXA1	17.14209823	-1.023941165	0.452608	0.00380596547738552000
FSHR	6.175931555	-1.76105517	0.85297	0.00383768095698757000
TRH	76.70535802	-0.951777591	0.277853	0.00384393408230180000
SLC30A2	15.67068348	0.724333695	0.486242	0.00384754629230940000
BCR	42.68177416	-0.744703685	0.286828	0.00391984740783144000
IQCH	13.87056277	-1.364443709	0.534478	0.00396501077258316000
HS3ST1	5.740701881	-0.955012128	0.683512	0.00402657452394777000
GAB3	8.864886458	-1.898106769	0.609841	0.00403410840231673000
GRIK1	32.30857692	0.800920396	0.50749	0.00405470379983569000
AEN	7.664249407	0.97804334	0.916277	0.00409184105993109000
KLHL17	26.76079775	-1.513379347	0.593328	0.00411138388703453000
KIF26A	139.9638784	1.865511118	0.599933	0.00421839951211983000
LOC112449607	6.081613587	1.131539655	0.777689	0.00427528423477457000
FBXO33	8.873433943	-0.901114071	0.678154	0.00433356626865288000
PECAM1	9.420420236	1.469215907	0.45523	0.00440729614365303000
SHISA9	10.74440448	1.810955686	0.57917	0.00442222653775458000
TST	97.83248221	0.72065764	0.205427	0.00445553342850356000
LOC112445938	416.8512529	0.861447837	0.417224	0.00460101477561766000
LOC101905498	10.37531337	1.180870689	0.406842	0.00474323692824487000
SLC1A3	36.94780971	0.71511817	0.46121	0.00482540163532503000
LOC112449260	12.71651395	-0.986356611	0.610575	0.00484479894522071000
ZNF365	9.213674053	-1.112784887	0.611947	0.00486299821208188000
ETS2	9.831713917	-1.665538505	0.675645	0.00494098924451982000
PER2	10.51120771	-0.916547782	0.597989	0.00494098924451982000
LOC112441470	19.4778268	0.8886532	0.376817	0.00495795644975231000
LOC101906008	167.6878646	1.014374627	0.301414	0.00502229414365865000
GRK6	40.07691363	-0.853758568	0.357382	0.00512576780462676000
LOC783497	17.89801911	1.496625989	0.560792	0.00525989375608328000
GRB7	11.16333407	-1.36204997	0.538984	0.00534393868448227000
GIMAP8	21.73432295	0.920986063	0.508887	0.00539420181632530000
PTGDR2	85.92974542	-0.941479759	0.311543	0.00539769570087298000

RNASEL	33.04864121	0.91625701	0.374896	0.00540493720860125000
LOC112444729	4.868765694	1.092259365	0.761948	0.00556331045288066000
RPS27	25.20382365	0.805634604	0.584464	0.00563699065822301000
BASP1	9.119397979	-0.738285878	0.543989	0.00576988650738601000
CCDC8	129.6423288	0.724370878	0.26816	0.00579345220925755000
IGFBP3	21.53320509	-0.744188677	0.339485	0.00580254977635629000
B3GALT4	7.403725546	0.780159676	0.544522	0.00581714352778120000
MED23	10.15841505	-1.166692335	0.684621	0.00585080635852417000
COL27A1	9.036172017	-1.268016446	0.550019	0.00590081527539526000
PMVK	3.037093378	-1.520822333	1.255083	0.00612879610260932000
PNPO	4.453234298	-1.451429098	1.036773	0.00632754000974370000
LOC112444967	9.047527671	0.965637082	0.520806	0.00644172095085030000
KCTD12	15.19848381	1.170377819	0.403835	0.00644182782657811000
ARAP2	19.20976769	-1.396323694	0.597632	0.00664873714042968000
LOC112444864	111.8219442	0.858254966	0.252961	0.00668719414746384000
ULBP21	23.54577893	1.021506301	0.615887	0.00670240979956991000
ARC	6116.199361	-0.741432704	0.222983	0.00671306037826200000
LOC101906363	14.57329424	-1.245833816	0.459943	0.00696529348007006000
TTC8	45.53910646	1.177109176	0.433116	0.00715559399777835000
CNR2	18.99707656	0.710548219	0.512042	0.00725270661679199000
CFAP58	18.80675316	1.553365644	0.587685	0.00727160731426593000
PSPH	50.94126544	-0.861123203	0.285439	0.00731731089659454000
LOC530653	56.86582286	-0.995096688	0.674001	0.00750351690686603000
FILIP1	24.7837868	1.038266117	0.368213	0.00776928695980678000
MYOM2	4.634446491	1.311983704	0.728767	0.00785556970365273000
CCRL2	21.86034965	-0.725726399	0.38431	0.00785989026888210000
FAM78A	134.9929568	-0.751020587	0.295697	0.00788657878957365000
MMP9	8.400531455	0.774431854	0.559414	0.00811888784085855000
HES1	71.66145301	-0.964277959	0.269609	0.00827257076543011000
GAL3ST2	6.035505176	-2.2753311	0.862082	0.00852675509711807000
LOC514658	29.30070021	-0.701335904	0.410377	0.00858070864428687000
SHISA2	11.98038983	0.701513141	0.50138	0.00862616958314041000
XXYLT1	55.26563221	0.840253839	0.312458	0.00870600331681614000
LOC101907142	9.605035927	0.841196659	0.617214	0.00873097084754001000

CALB2	179.348745	0.80517123	0.23312	0.00878147933114790000
LOC112443796	21.30795437	-1.250698529	0.646799	0.00881238696182083000
BAIAP2	20.03092044	-0.822016581	0.42168	0.00884057072730975000
SYT3	20.85618862	-0.791343471	0.518189	0.00897439235842265000
SGMS2	28.76782047	-0.821697457	0.427455	0.00901319651146771000
CYP2C19	31.9269878	-0.730610409	0.31405	0.00905241387652633000
LOC112445246	15.73839374	-0.889540284	0.410659	0.00914696780305945000
FAM43B	7.194788476	1.15462104	0.501227	0.00921801258888582000
LRRFIP1	21.40232612	-1.084991478	0.386211	0.00950848052058062000
RIMS3	17.36109668	-0.806270141	0.40649	0.00954322228367479000
TMEM182	51.00485376	0.821598726	0.362878	0.00954322228367479000
ATP1A2	5.616046782	1.216379709	0.721789	0.00961480959713919000
SLC16A3	25.62944201	-0.728733892	0.303188	0.00966231751813726000
HOMER2	13.50474794	-1.219823802	0.557364	0.00973727016041164000
KCNB2	4.718367953	0.804754666	0.773298	0.00975658273701064000
FRMD3	40.09252806	-0.880341468	0.30334	0.00980334847622142000
PDC	11.24995413	-1.294300732	0.547355	0.00981687508246426000
GFRAL	55.41772987	1.120555891	0.418524	0.00991513162501619000
SSC4D	15.22877954	-1.308741095	0.468538	0.01004323663513300000
LOC112445042	34.36559039	-2.234955	0.778207	0.01009215985898430000
EML1	9.642491388	-0.94166146	0.507504	0.01011993994475430000
CAVIN2	7.264236928	-2.044788643	0.715823	0.01017922284011410000
FAP	9.613966845	-1.444977375	0.791178	0.01028782390217100000
COL12A1	12.50720009	1.848495788	0.620713	0.01032013274893030000
NPTX2	9.24639962	-1.798536403	0.722308	0.01032085411985430000
UBXN10	12.89299189	-0.905399606	0.52442	0.01039900431620060000
SCARA5	37.07990728	-1.188489562	0.334209	0.01050171298909560000
LOC783362	838.7811911	0.82349405	0.278449	0.01058251070712630000
CYP51A1	8.019618041	-0.701182335	0.541638	0.01066717928020280000
PPYR1	57.07301748	1.702488012	0.651661	0.01073937442884580000
MFSD6L	5.648569716	-1.426148346	0.771611	0.01077413393265080000
MEGF10	32.8348979	-0.787720132	0.328265	0.01109112056163700000
ANKRD50	27.43486139	0.908382615	0.378688	0.01120064319118270000
LRRK1	11.44674855	-0.918151042	0.546303	0.01132650892077930000

LOC789148	5.899447521	0.737768698	0.795881	0.01151961996514690000
TNFSF18	4.762382574	0.920337452	0.888642	0.01156326361069850000
SLC1A4	37.54369496	1.480517679	0.53668	0.01187663746004640000
LOC101906006	11.4825932	-1.244871792	0.459737	0.01194753855337960000
SEMA3C	6.620681896	-0.772449729	0.586615	0.01201154072542860000
FRS3	18.89579734	-0.985869831	0.504041	0.01217677333699120000
PGLYRP2	867.5847858	0.738122864	0.229865	0.01220975201894760000
MAP10	56.66719669	0.712618854	0.419593	0.01227557675113500000
HSPH1	74.55822465	0.805140351	0.328429	0.01230192333002870000
SLCO4C1	23.79679043	-0.796332136	0.332711	0.01234795040983930000
ABCB1	5.38776002	0.840203158	0.58968	0.01240982011689180000
DSG2	14.07574113	0.721508101	0.497206	0.01289799251284730000
NWD2	50.72223575	0.843827316	0.318385	0.01290893884553240000
LOC104974017	12.65924147	0.757297168	0.540047	0.01305145295862850000
MET	126.3562728	-0.914066641	0.291171	0.01313035474788730000
GATM	5.455273758	0.784044809	0.811069	0.01324961537045790000
TLCD2	35.96227139	-0.985705797	0.321191	0.01335500425316440000
CNNM2	5.548622051	1.834234015	0.920741	0.01345287124954840000
GAT	5.00805545	-1.513995306	0.926216	0.01368478389708140000
CLIC6	32.4553515	1.392285184	0.541878	0.01395626781248230000
ITGA7	7.075152694	-1.473460755	0.593991	0.01403644236508570000
SSFA2	16.14330267	0.880490003	0.478732	0.01434120528541110000
CCDC87	46.46746511	-0.891448102	0.403089	0.01434562430538540000
S1PR5	80.79297839	-0.754970758	0.272082	0.01449567726356270000
SMPD3	26.37180471	-0.815558548	0.488314	0.01449567726356270000
LOC112444493	18.17854151	-1.041480741	0.475274	0.01471766735652480000
NAIP	18.0531693	0.711719661	0.499884	0.01480870390247110000
STIP1	4.085539716	0.96482233	0.746751	0.01513556794211540000
HCN1	7.287711819	-0.873501041	0.740469	0.01549528606355930000
HUNK	28.14563433	0.766646712	0.455832	0.01575247600963390000
ZNF862	31.46452941	-1.045517496	0.414083	0.01605118630450860000
CNTN4	4.178086392	-1.312198289	1.074236	0.01621812184335710000
BRINP2	89.34244101	0.793584661	0.327771	0.01662540375415060000
CDC42SE1	253.5993159	-0.761711203	0.433559	0.01671295014326040000

SARDH	46.22939085	-1.100385852	0.385799	0.01684771484690730000
RINL	9.0775482	1.438631024	0.631895	0.01686502070234170000
TRPV6	18.87072944	-1.894948421	0.655452	0.01690944922840310000
LOC112444511	7.853623503	0.842219967	1.47608	0.01694898852747770000
YOD1	11.38275046	-0.94524026	0.507765	0.01700674239722160000
SSTR1	4.214331103	-3.010989316	1.334687	0.01711432567400360000
LOC112441472	12.55271145	-1.155526854	0.505556	0.01718578501391020000
DUOX2	60.46749041	-0.707181483	0.573158	0.01725295794614180000
DTX1	43.69551013	-0.724248069	0.295715	0.01728040516379330000
RHOB	9.504372836	1.531608476	0.500241	0.01744595458669660000
ARHGEF2	5.385994693	-1.076000646	0.690512	0.01756516211099410000
SERPINB2	9.018105917	-1.133083764	0.531639	0.01777040369185260000
SLC38A3	61.5215676	1.441186796	0.580028	0.01781254542461680000
CPQ	33.18242092	0.714034415	0.485308	0.01783511219532500000
PRR5	6.439499219	0.837201572	0.689207	0.01829198404822350000
PRIMA1	15.42098853	0.828425844	0.378665	0.01839055813273470000
SAP30	14.19758879	-0.737565153	0.397346	0.01861352437182180000
RGL3	29.50027366	0.98228128	0.348831	0.01865080618425270000
CELF4	8.568760787	1.282737948	0.915122	0.01868356946727590000
LOC112449243	10.35688324	-0.805598511	0.583514	0.01876985665252180000
LOC509911	9.901656441	-0.946329001	0.502145	0.01916408771856670000
OVOL3	3.705365752	-0.796428667	1.184946	0.01925270758940680000
PTER	36.39600916	-0.70449071	0.399333	0.01958705796827340000
ASTN1	28.90273449	-1.05216178	0.377294	0.01965609872541480000
LOC507550	15.27117808	0.777081396	0.545779	0.01977314331494700000
TMEM132B	11.1072289	-0.732341423	0.685118	0.01998782773896700000
NRXN1	17.97440031	1.6765459	0.573811	0.02002741797385760000
DUSP5	6.28219879	-1.21388891	0.98898	0.02005582153876900000
STAC2	15.32672217	-1.399738367	0.576739	0.02009919921949250000
BTBD3	5.565946553	-0.955919438	0.758026	0.02026731827697080000
RAB39B	14.17114555	-0.709790667	0.498317	0.02034607198887880000
ANKRD61	20.78140087	-1.729562886	0.503133	0.02063364053933490000
ID4	12.89699819	-1.447028503	1.176601	0.02065633523525120000
SLC28A2	39.74820614	-1.006332945	0.661642	0.02132729467483520000

FRMD4B	7.909359291	-1.409480963	0.698006	0.02155669457076050000
LOC101903988	34.33174617	-0.972943409	0.470744	0.02155684074735810000
SLC5A9	22.97594148	-0.717599156	0.419215	0.02162532500599440000
LOC112444959	10.93108463	-1.525376873	0.705173	0.02186153256516790000
FHL3	93.80045744	0.745625158	0.244857	0.02234651664953970000
LOC101908014	5.034707986	1.919750245	0.766033	0.02236976892255120000
LOC101906240	7.88615951	-0.890551158	0.594383	0.02316887315925130000
SLC19A2	5.263166007	-0.872062823	0.711357	0.02356212626341810000
RBP4	5.312100855	-1.923717316	0.840378	0.02373704360909740000
STXBP5L	5.396465944	-1.109927907	0.779823	0.02393788125241330000
GPR22	2159.382483	0.714493261	0.401928	0.02396883574493690000
F2RL2	10.97730872	-1.725412051	0.729582	0.02399797140061760000
DYDC1	233.4983951	1.394929496	0.450367	0.02404312025013110000
CPAMD8	8.729334911	-0.971035609	0.885242	0.02416828501508550000
INPP1	29.34310159	-0.851547547	0.495767	0.02425686538373060000
GABRR3	8.795079548	-2.574084893	0.73501	0.02452274148248930000
LOC783600	51.64042999	0.836998325	0.297101	0.02458351526710190000
TSPEAR	8.805877152	0.724180077	0.564046	0.02481297237338090000
SLC25A35	16.30063934	0.907082348	0.378261	0.02483137457269450000
LOC112445492	16.44029789	1.099440592	0.546215	0.02490344142485950000
CCDC183	9.44098342	-1.277369285	0.555683	0.02502836742897280000
NXNL2	84.6119022	-0.730913117	0.294971	0.02532461456386430000
PRRG2	25.67062697	-1.137452154	0.465967	0.02543612239191960000
LMNA	16.47178506	1.112486022	0.601473	0.02624542442370490000
KCNE4	14.00161962	-1.048100149	0.489266	0.02650219988868120000
COL25A1	128.982381	1.387311566	0.46707	0.02668970252952560000
FOSL1	48.77753085	0.950715987	0.414863	0.02673186107715940000
GSDMD	11.98800286	0.95688523	0.769988	0.02674175185932840000
TACR1	49.72212001	-0.753101835	0.423927	0.02686700914061650000
ANKUB1	31.68489285	-0.907637958	0.317072	0.02719375748211960000
GLIS2	11.88231683	-0.743826447	0.535764	0.02777796232623010000
LIMCH1	8.575782716	-0.706016152	0.542365	0.02778398955061310000
STRIP2	54.52518304	0.8260516	0.329737	0.02781207564865940000
LOC112441849	5754.992603	0.727033522	0.371301	0.02787171347626910000

LOC101903858	8.511183475	1.344573423	0.495387	0.02824268925317230000
LOC521224	93.43240423	-0.708550829	0.236996	0.02849559921433800000
LOC104972499	9.257656582	-1.289080894	0.641175	0.02856849553140480000
SLC9A9	8.849242857	-2.344407073	0.769125	0.02912814253838380000
C1R	8.169153435	-0.952159914	0.585795	0.02960258957734460000
MAFG	14.45612659	1.565211354	0.536521	0.02963706178802260000
SLC25A27	29.13277599	-0.718044719	0.367542	0.02983369337899640000
ARHGEF4	553.559378	0.748658225	0.258279	0.02985848343067760000
SETMAR	4.558710963	-1.38548919	0.86289	0.02997245595123790000
COQ8A	60.05323142	1.031925643	0.464619	0.03056102520367940000
METTL13	20.12769322	-1.284660325	0.409998	0.03057445928626580000
FLT1	229.0302811	1.195705695	0.336546	0.03123392342818910000
KLF13	25.93834688	-0.741417865	0.402028	0.03167569783233470000
LOC112449278	6.355104044	1.118755134	0.657399	0.03188958762608800000
SMIM18	21.19173417	0.725894001	0.34758	0.03200995998914250000
GRM8	11.99743946	-1.041173739	0.550712	0.03210120483930000000
LOC112441473	5.020921431	-1.586111684	0.853053	0.03218501943311500000
FDX1	10.44561899	1.340858813	0.55711	0.03334958870714620000
SAMD13	4.668732005	-0.917228363	0.939248	0.03380489460795900000
ADAMTSL3	6.91996985	-1.509730324	0.757443	0.03394439347695020000
IL12A	19.28326301	2.220877816	1.377998	0.03424339251203910000
LOC112444532	10.34167055	-0.967297565	0.631817	0.03464835922059380000
SLC10A6	211.8477742	1.390761911	0.491461	0.03465773966437510000
LOC112446053	61.30017649	0.965925497	0.386289	0.03588323442519570000
DRC1	6.092733408	-2.258243453	0.831296	0.03589901302138170000
HTR6	11.6079222	0.770247328	0.391951	0.03601087793686360000
LOC112441834	4.685502544	-0.775693671	0.855918	0.03605530969696740000
ARSI	44.76072267	1.077263235	0.383841	0.03648398599302220000
PLK2	844.1623098	0.972421814	0.400135	0.03668822900436870000
NR0B1	6.68569013	0.906740394	0.559351	0.03676375704128260000
SMIM4	17.18348107	-1.053153661	0.445551	0.03685536043042830000
SGK1	4.789937902	1.632750837	0.861894	0.03719513029653580000
PPP2R2B	5.621075542	-0.754440291	0.713509	0.03758526434712850000
AHSG	33.4653312	0.776545667	0.372975	0.03854186580857290000

GPRC5A	6.383169241	-0.910887055	0.829701	0.03875675231559490000
KYAT1	10.44547745	-1.798443512	0.548991	0.03924461144348940000
URB2	136.1885337	1.38775657	0.466352	0.03931937492445840000
LOC528412	10.90043874	0.846617151	0.659323	0.03963339778905850000
SUSD5	6.900166053	-1.171039281	0.990562	0.03969074963709560000
LOC101904622	7.926150566	-1.481798923	0.621236	0.04051259093711980000
TUBA1C	4.103161426	1.040665774	0.696637	0.04067880767806050000
KRT8	100.6109577	0.77437957	0.300503	0.04076229898862450000
LOC786139	31.58252754	-0.822154922	0.334744	0.04079903631722520000
LOC104969038	3.454506486	-2.380788542	1.193699	0.04095878344982270000
PDZD7	32.77893572	-1.065437416	0.420658	0.04120709914680640000
LOC104973489	5.766071094	-1.814615878	0.806345	0.04130493277164390000
AQP3	302.8709583	1.112075045	0.822572	0.04163251431438120000
PRDM16	6.688628888	-1.024100056	0.617216	0.04165005466888180000
LOC101904398	10.22437492	-0.718457303	0.503956	0.04195129737874560000
DMRTA1	5.841706367	-1.166611927	0.868912	0.04200844999764280000
DTX4	139.2503331	0.799276115	0.31346	0.04224830242902610000
MAFF	5.536511789	-2.064057028	0.753168	0.04235235269556030000
ARHGAP26	40.5572464	0.702090583	0.344342	0.04406314360526880000
GPR137C	3.923788434	2.137359768	1.072173	0.04461581432117490000
APLP1	19.7553713	-0.724860773	0.397932	0.04501216195159380000
CHORDC1	5.546387379	0.947830123	0.731527	0.04525976757994260000
ANGPTL6	9.479346627	-0.71194274	0.645573	0.04531073469756760000
COLEC10	14.87529817	1.235309532	0.506148	0.04551253621835860000
RNF125	6.226988549	-1.474363919	0.727753	0.04600082299250440000
XKR8	20.73537272	2.170004862	0.844124	0.04633460196174150000
RPL3L	6.690036026	-2.296796614	0.924395	0.04673226771283190000
LOC101906457	8.811344379	-1.077863103	0.642628	0.04686998311606380000
PDE5A	7.194365894	-0.852763201	0.704322	0.04773696194085320000
NPR3	28.94571464	-1.135673333	0.354284	0.04776051570223750000
METTL4	7.256019512	-1.236142324	0.737535	0.04779319699191910000
SEPT4	5.592797402	-1.156434326	0.764565	0.04781259817137480000
TRIQK	43.13530918	0.815207962	0.83373	0.04793867302515620000
KCNH1	21.0800454	1.192588122	0.399836	0.04820467920132100000

YARS	7.368074498	1.229267953	0.787196	0.04831327874930060000
PDXP	14.01102755	-0.82329918	0.559085	0.04881482247750300000
LOC107133209	31.37830269	-0.824775823	0.353247	0.04896830981043340000
SELENOP	3.377407072	-2.106171874	1.27236	0.04910903986227870000
FCRL5	32.17084388	-0.795028424	0.377713	0.04936183106522450000
ID3	19.17037981	1.071369937	0.45983	0.04965319974774670000
