# Supplementary material for: Extracellular Vesicles Derived From Antral Follicles Significantly Change the Transcriptional Profile of Cumulus Cells and Oocytes During Pre‐In Vitro Maturation in Cattle
Source: Mol Reprod Dev. 2025 Nov 24;92(11):e70068. doi: 10.1002/mrd.70068 (PMC12645189; doi:10.1002/mrd.70068)
Supplement: Supplementary file 3 — Table S2: Differentially expressed genes in cumulus cells of Control vs. Late EVs. [file MRD-92-e70068-s006.pdf]

Table S2. Differentially expressed genes in cumulus cells of Control vs. Late EVs.

Gene	baseMean	log2FoldChange	lfcSE	padj
MYOCD	537.95787447928	0.73691322	0.227852728	0.0000000000000000000
KATNBL1	791.36164047876	0.71050828	0.124138551	0.0000000000000000000
S100A4	795.54228536559	0.88205339	0.465564183	0.0000000000000000000
CREB5	278.41610171210	0.65201512	0.214438482	0.0000000000000000000
PRRC1	102.05679510613	0.64219753	0.14364283	0.0000000000000000000
ZHX3	281.05951317899	0.64331930	0.174275554	0.0000000000000000000
FLVCR1	251.22911026801	0.66844827	0.234941428	0.0000000000000000000
IER3	332.20052618821	0.68030534	0.214854102	0.0000000000000000000
CDKN1A	108.79739150778	0.71034688	0.192715971	0.0000000000000000000
SMTN	577.18630863438	0.80892911	0.211834887	0.0000000000000000000
STK40	245.57060162921	0.63717001	0.171528644	0.0000000000000000000
GARS	308.88217699460	0.63893553	0.17400744	0.0000000000000000000
MAFF	907.94274754833	0.82367916	0.211207052	0.0000000000000000000
SLC1A4	330.38152628044	0.63693784	0.284362722	0.0000000000000000000
PIK3IP1	345.26275532572	-0.94651611	0.180537953	0.0000000000000000000
TEX264	499.16473573127	-0.64878494	0.136249942	0.0000000000000000000
CDRT4	121.77732677989	0.87680820	0.274753376	0.0000000000000000000
LOC112448164	423.14587744092	1.64940337	0.37166904	0.0000000000000000000
GDNF	29.29313198142	0.71050785	0.319106699	0.0000000000000000000
LOC104975039	162.76584689010	0.61345600	0.467589151	0.0000000000000000000
FAM83G	10.31799548376	0.62106271	0.190710391	0.0000000000000000000
LPP	222.73417890918	0.69005289	0.215207647	0.0000000000000000002
RIMKLA	187.85812865371	0.62201611	0.153037681	0.0000000000000000004
BCAR3	978.05105081358	0.62055974	0.15334309	0.0000000000000000005
LMO7	390.43207746525	0.73702025	0.227425107	0.0000000000000000016
EPHX1	263.67342401841	-0.99788593	0.186068623	0.0000000000000000023
ASNS	201.44198337012	0.71748165	0.197124235	0.0000000000000000294
CAPN2	400.02430855066	0.60665160	0.134373285	0.0000000000000000425

MOB3B	438.09413882071	-0.61701753	0.166120481	0.0000000000000000649
FZD10	457.01699526950	-1.72516290	0.331642816	0.0000000000000000929
NFKB1	555.48171451743	0.69691150	0.191881584	0.0000000000000001880
LRRFIP1	138.12507357967	1.03104231	0.225926942	0.0000000000000004550
ELAVL3	177.21835119673	-1.17482293	0.45725929	0.0000000000000007200
SLC4A7	153.71424186313	0.78823139	0.212426271	0.0000000000000009890
TMEM100	576.76333655024	1.66691769	0.35992558	0.0000000000000015000
FABP5	177.87598246735	0.71032250	0.213696131	0.0000000000000140000
PDIA2	871.17112095477	0.84279325	0.396461399	0.0000000000000668000
RASSF1	495.11863013421	0.64546180	0.144121477	0.0000000000001280000
ARHGEF2	369.29151190141	0.65632108	0.22199071	0.0000000000005800000
SV2B	160.27873935188	-1.69680092	0.32802369	0.0000000000012100000
ZNF696	335.10477147575	-0.67583330	0.200604753	0.0000000000013300000
SLC20A1	34.44196282605	0.70937535	0.161476481	0.0000000000029300000
BCAT1	109.03554212200	0.92472131	0.259562188	0.0000000000036700000
ARL4C	973.31664438648	0.69933841	0.244960046	0.0000000000044000000
PPP1R15B	118.49941692945	0.65335719	0.212907499	0.0000000000052700000
UBE3D	101.94070521898	-0.88497930	0.232466001	0.0000000000061700000
IPPK	252.52435352167	0.63907005	0.188764944	0.0000000000241000000
LOC505099	394.65283124644	-0.62370771	0.311432394	0.0000000000570000000
EXO1	26.25984155526	1.00099426	0.280722611	0.0000000000650000000
IL22	347.40192555924	1.57154809	0.396809847	0.0000000001580000000
VILL	245.08348936388	1.09584247	0.187155082	0.0000000003390000000
SSFA2	304.07837073094	0.76562831	0.235780078	0.0000000004020000000
CHD1	170.42933660170	0.68245873	0.211686263	0.0000000006960000000
DNAJB14	746.13186113210	0.65835023	0.177842461	0.0000000008420000000
B3GALT4	13.38750558288	-1.00107979	0.24613291	0.0000000018600000000
LOC104972403	165.60610640713	0.60738315	0.224043505	0.0000000024400000000
LOC112443229	285.42423887224	-0.63949184	0.216583788	0.0000000034200000000
MVB12B	400.86450467369	1.10060362	0.21556831	0.0000000043700000000

IFI6	976.06187669138	-0.64301976	0.283748256	0.0000000045100000000
MAP2	581.11839738347	0.88988387	0.217741811	0.0000000055200000000
MANBA	125.44098556192	0.64296211	0.135923939	0.0000000060500000000
TCF7	290.80074698248	0.73208737	0.194248845	0.0000000076500000000
FRMD3	41.46455959598	-0.75327257	0.286003409	0.0000000078800000000
SYN3	833.87906920578	0.63721700	0.179718804	0.0000000377000000000
SARS	250.49456458564	0.69991526	0.178397929	0.0000000378000000000
RASA2	351.99926983798	0.92097830	0.192947827	0.0000000500000000000
PSPH	971.95613948546	1.07203547	0.257638463	0.0000000588000000000
CNKSR3	57.44155275189	0.61453854	0.180403931	0.0000000896000000000
LOC101901998	865.45952780526	0.99133808	0.798025514	0.0000000985000000000
TRIL	234.37149575550	-0.64081401	0.278360837	0.0000001210000000000
LOC112448165	439.98255129965	1.19889731	0.420926728	0.0000001260000000000
HSF2	874.96332377856	0.69329917	0.148521213	0.0000001300000000000
LOC112446730	240.80789402763	0.61212396	0.195355769	0.0000001670000000000
NAA25	679.04048273641	0.76365261	0.186247644	0.0000003570000000000
GPR75	164.75829387214	0.67034004	0.18516852	0.0000003840000000000
SLC19A2	934.82980073492	1.40022823	0.267312822	0.0000005130000000000
LOC112443216	148.51947142875	0.74421666	0.265465804	0.0000007120000000000
ATP6V1C1	304.86164725914	0.63857587	0.168129234	0.0000013200000000000
HIGD1A	455.73183735011	0.61426335	0.218829705	0.0000023100000000000
RIPK2	440.26467751630	0.85562778	0.216309007	0.0000023600000000000
ARMC4	626.42470714401	-0.61923047	0.175841643	0.0000025600000000000
MDM1	103.93272944239	0.83278411	0.254112578	0.0000027500000000000
CROT	328.50794743134	0.66690469	0.192520945	0.0000033700000000000
LOC112443006	167.06346239706	0.61696426	0.140271446	0.0000047100000000000
XRN1	965.33627120109	0.61507138	0.177002452	0.0000048500000000000
ACBD3	129.44347020315	0.64023573	0.161382344	0.0000053800000000000
LOC112447762	985.14380943325	-1.44622039	0.326803855	0.0000064500000000000
DNAJB1	431.14508647406	0.61321851	0.232228789	0.0000113000000000000

TCAF2	996.83528977891	-0.73890000	0.262690085	0.0000131000000000000
SLC11A2	804.82903903687	0.64726639	0.153963642	0.0000203000000000000
FOSL1	393.37840267030	0.86969899	0.196983572	0.0000322000000000000
B3GNT3	317.09204892022	-0.85343965	0.172498906	0.0000532000000000000
LOC101909455	233.03140337965	-1.14143734	0.200642836	0.0000544000000000000
ID3	21.69258833383	-1.13715431	0.208865649	0.0000852000000000000
NFKB2	925.15134580732	0.78295542	0.150757965	0.0000985000000000000
CCDC183	46.00455311570	-0.65406467	0.297633743	0.0001003155703361510
ARHGEF4	406.44905202601	-0.61378912	0.356418153	0.0001052374656064220
TNK1	539.94475805383	-0.72125320	0.256511752	0.0001078503104353840
SPATA33	239.68874351353	-0.73327402	0.40771638	0.0001095879657999270
LOC101902854	173.59757464783	-1.35869382	0.449785557	0.0001103240868904920
P2RX7	89.30242649598	-2.96009274	0.795403912	0.0001103949207751010
CPAMD8	434.86892710753	0.74200193	0.379418169	0.0001139927020415590
PTER	770.35182183102	-0.72240225	0.242575176	0.0001142076723913150
LOC112442271	150.72417762727	-0.90591099	0.45755999	0.0001146268348489020
LOC782527	234.72996402412	0.62418297	0.335481593	0.0001146298326175650
NEDD4L	80.99666779564	0.99836641	0.299236155	0.0001151163041793400
LOC107132092	928.02259735296	-3.48269028	0.936160025	0.0001159208393891220
SCML2	133.25768864029	0.68527323	0.266518127	0.0001170903939609220
FAM133A	364.78584078038	0.63836752	0.277030693	0.0001175450290410250
LOC112442686	144.23713375747	1.41633311	0.588395593	0.0001191660901696110
SP100	446.61867213631	0.72187427	0.419261069	0.0001206216246703710
DUSP18	139.37212199431	-0.67860304	0.562605762	0.0001279489216597290
TJP1	768.47127675533	0.69700939	0.263883868	0.0001298063801574280
LOC112448103	907.21557178054	-0.92023337	0.646475885	0.0001301487609012850
DYDC1	457.97457111550	-0.91247160	0.356806905	0.0001383543267162880
LOC530653	27.87301406825	-1.99137270	0.385320739	0.0001390000000000000
TJP2	510.67924532427	0.68567820	0.219121679	0.0001400446209889030
RIMS2	565.38479598870	0.62313618	0.28773145	0.0001420897561697600

DNAJB11	210.27162951045	0.89162901	0.239091296	0.0001430000000000000
ALDH1A2	369.12954365454	0.98663883	0.464855672	0.0001438472362536640
REELD1	788.08489637494	-0.60786898	0.775535539	0.0001444358729202870
LOC789694	371.50501737515	0.78398015	0.336246031	0.0001446213083589670
HEXIM2	698.29288589690	-0.76373265	0.215483591	0.0001462227178112870
LOC112449175	623.10606571800	1.05437734	0.574378209	0.0001507210394792950
TRPC5	49.15123048842	0.93475498	0.37814172	0.0001527863888505620
PPP1R14C	130.96822305016	1.04363302	0.351299273	0.0001538629381343420
PRRG2	792.97661548654	-0.73114490	0.579334324	0.0001565982995779520
DSB	566.79516901100	1.38038932	0.810437056	0.0001651195480018680
MAK	529.72483843981	-0.65282356	0.363255465	0.0001675151733833770
IL2RB	785.47590027093	-0.64664418	0.675182589	0.0001684788591254590
LOC104973829	774.74958219804	2.23060789	0.638614918	0.0001724514980133380
LOC104971004	621.60320773111	-0.95352452	0.79612073	0.0001753856728871870
TMEM35A	997.92493216286	-0.60243959	0.311515062	0.0001772213343076080
ZNF516	532.19921747655	-1.93331491	1.0251444932	0.0001781134611655610
MGC137055	178.23886174702	-0.90145551	0.411706	0.0001781390281191750
SPAAR	661.20560317624	-1.20731246	0.782175714	0.0001785268362183340
RUNX3	884.75176364056	-0.63597013	0.274679716	0.0001789815166922900
PRIMA1	317.85466438037	-0.89694211	0.446208071	0.0001792999091013330
WDR87	151.26317178211	-0.87678621	0.472437607	0.0001805223593490590
PABPC5	203.62712904316	-0.72581794	0.430331477	0.0001806701239107780
RASSF4	255.93442732214	-0.82764651	0.223278909	0.0001811196883403370
LOC112442189	349.67378132149	-1.52209544	0.377750967	0.0001841853457561280
LOC112449613	495.92497596821	-0.65021327	0.403953902	0.0001862770843620300
LOC112449123	522.62837658621	-0.61196045	0.362496626	0.0001865750057442220
EHMT2	364.68261966644	-0.61259989	0.21740263	0.0001870385022340430
TEX11	487.10627081314	0.82899521	0.300087312	0.0001878515055436690
PPARGC1A	131.43980317594	-0.88730212	0.664784967	0.0001889000134586690
CPA3	58.24967942684	0.64382347	0.35639682	0.0001894281861266000

GJA5	576.60183796555	1.31980901	0.567144589	0.0001927579361365560
BIRC3	332.91668643266	0.93727158	0.186414503	0.0001930000000000000
FJX1	146.52571837060	0.85135823	0.341554968	0.0001961502258481810
LOC104970821	430.84160113868	-0.69229182	0.326966488	0.0002006960136609000
IL15RA	40.54217254528	0.77890507	0.203440849	0.0002046634699066070
HPCAL4	488.29974242336	2.19110711	0.927250439	0.0002182063765935800
ULBP21	476.53648044091	0.70826681	0.36066995	0.0002196110904605100
VIPR2	102.42827551887	-1.41561220	0.655739096	0.0002221278092713900
LOC107132465	217.88268114031	-1.19083619	0.468482884	0.0002256825617085780
LARP4	398.06943108509	0.77738056	0.193107035	0.0002269055834308330
LOC107131209	292.58309454633	-0.70157241	0.333097231	0.0002293724352359960
C3H1orf52	130.20055421074	0.68722386	0.180254707	0.0002320738152442870
ZNF213	30.83279468915	-0.73153408	0.317171769	0.0002367517287251510
OGDHL	602.48401530273	1.83382994	0.896356679	0.0002367517287251510
NRCAM	139.92338500783	0.66267885	0.218688253	0.0002371152520082630
YEATS4	221.88559294770	-0.89741909	0.216626655	0.0002371770637109360
UBASH3B	115.52161023630	0.60573574	0.341651705	0.0002375889287741770
ZFP36	325.22349325859	0.67716721	0.210122643	0.0002393803883713470
NR0B1	15.23987470499	-1.28512649	0.543699452	0.0002434382059465180
SLC38A5	122.15216086466	0.69165867	0.219075827	0.0002455515594251670
CALB2	956.43420092707	0.91100532	0.205301863	0.0002460000000000000
C15H11orf42	145.49610355359	1.29112648	0.511508511	0.0002492806859500190
ANKUB1	181.49029824535	-1.07176552	0.426434448	0.0002524068044728760
LOC100335340	84.39827343859	-0.91766917	0.289307893	0.0002548394073900440
NEDD4	28.16284277281	0.63100105	0.150889684	0.0002560000000000000
AEN	753.64434514079	0.61404449	0.177573646	0.0002600000000000000
LRRK1	790.37734411595	-0.69275076	0.293350188	0.0002601892317105540
UHMK1	694.05099836667	0.65336907	0.30796319	0.0002630482997893350
SSH2	148.55380407371	0.60599581	0.245986779	0.0002698992578145330
TIGD3	140.94001308098	-1.37255106	0.621047296	0.0002795836041062320

LOC101906344	153.55787389623	-1.63767536	0.50370747	0.0002809969819843850
PPFIA4	268.28244619926	-1.04467866	0.377088585	0.0002838492878918090
TDRKH	355.89277966683	-0.92809219	0.435357972	0.0002921640719808980
SH3TC1	254.27329982331	-0.73898907	0.537473879	0.0002974412728826220
ANKRD34B	526.63097436271	-3.46474421	1.0773775889	0.0002981944404775780
PRKAG3	599.68660585639	-0.99652718	0.340073658	0.0002992867869667390
DAB1	395.88274395521	-1.28495337	0.376194239	0.0002996456686680900
LOC104969384	125.91304626253	-1.19364612	0.534082987	0.0003077636048886910
HIST1H2BL	401.10647136494	0.77859278	0.350468038	0.0003107501544400430
LOC531090	333.95152032716	-0.69608101	0.370907512	0.0003154832758954940
RBM46	948.35096866984	-0.93177893	0.339965874	0.0003160684852481230
VGLL3	6.03350858762	0.60206939	0.302034712	0.0003165334607111540
DOT1L	235.06383895588	0.62495817	0.218610182	0.0003250000000000000
MAP3K9	401.86438274342	-0.79917655	0.301626202	0.0003302892089170100
LOC101903858	140.47731712010	1.42733465	0.371718513	0.0003348879508157320
LOC104975022	224.42558039700	-0.73373953	0.451956072	0.0003349169235517540
LOC112447073	292.73533719805	0.62571045	0.379012231	0.0003393781268961850
PXMP4	245.98886081620	-0.73353485	0.404306546	0.0003519377520175490
SEMA3C	180.79962686485	1.02231619	0.22789281	0.0003643634107231970
LOC101906008	941.23044990543	-0.67343359	0.616158341	0.0003672629428776350
NET1	415.20269082732	0.92497054	0.240305258	0.0003717991019893740
ELAC1	479.35303308954	-0.66109159	0.174528681	0.0003730000000000000
IYD	151.15994103070	0.94581577	0.320657681	0.0003756582827810170
ZNF169	794.02315107476	-0.91485940	0.309547714	0.0003793947832803010
YJEFN3	718.78990701271	-1.14866504	0.833004246	0.0003881678867003590
ABCB1	482.39125927543	1.22277445	0.290889493	0.0003887169635178460
MGP	769.65844537801	0.96141178	0.423730521	0.0003943880871302260
NTNG1	39.32573636432	-0.78828310	0.258256985	0.0003977860203953770
LOC104968522	247.26261831626	0.86965188	0.214373672	0.0003987674785372040
ALOX12E	123.65138847809	-1.13555124	0.603980937	0.0003993248955995930

DCLK3	436.71495952547	-0.82949431	0.336924812	0.0003993892060302620
FAM205C	420.30788251615	-1.05455492	1.1082324797	0.0004081350657432080
CBS	172.09970953330	0.88817641	0.280182823	0.0004207563299525040
HMOX1	10.57492086114	-0.73299145	0.395143591	0.0004250217694124040
FAM189A2	36.99120558548	-0.75621773	0.216365757	0.0004367987481036310
GPAM	319.90159216212	-0.63431380	0.200281554	0.0004384185567186030
GSTM3	109.77315636835	0.74327091	0.20882249	0.0004385100262483970
EREG	983.28432178293	0.65437334	0.392234069	0.0004627940333347020
GSTA3	523.63893114788	-0.61166154	0.411216979	0.0004726394604273390
PDE4B	316.64517238649	0.82188684	0.506481203	0.0004746124764741730
ABCA4	306.47206635911	0.80013330	0.450106479	0.0004809386151232980
METTL13	509.85809563632	0.81651951	0.215846208	0.0004874466359039200
CACNB4	779.39007589464	-0.78199845	0.619599452	0.0004896838356794180
CHI3L2	458.81103480846	-1.38567962	0.443531637	0.0004962658375491670
LOC112448495	1.64432810819	1.34734624	0.444234348	0.0004965500288596790
LOC101905357	542.63742399403	-0.80597932	0.326027844	0.0005207750124474000
LOC783497	299.15938574113	1.18455401	0.45060518	0.0005309692448065350
TRPM2	466.42106093618	-2.09108612	1.116909085	0.0005386489345061480
LOC101902260	777.96207744194	0.68908940	0.259508823	0.0005471428582720860
LOC112444963	907.70754230336	0.69256312	0.51510579	0.0005511108462083100
SYNM	508.88439405982	0.73278268	0.292699301	0.0005515937600204860
RIMBP2	840.82173407815	-0.92343720	0.630489866	0.0005548127131833030
AHR	886.81193972750	0.92006951	0.29059055	0.0005617181978287320
ARHGAP33	174.60741827331	-0.62139068	0.427038501	0.0005645981792882320
WNT5A	129.29635723196	-0.65938918	0.279026268	0.0005677724605310250
LOC112444276	346.87382912191	-0.61919354	0.343899064	0.0005704764421913290
HTR6	617.78110985898	-1.23621217	0.77253239	0.0005744272880699510
LOC112445031	801.69804857303	-0.62641968	0.618354468	0.0005879899189951070
LOC112446466	657.44605463451	-0.91817514	0.735135017	0.0005968196187311790
RBP4	765.17427872262	0.83085479	0.227086234	0.0006047157069712860

LOC112445761	205.64723671066	-0.62225741	0.494172489	0.0006232449408214670
ANGPTL4	119.44009785349	-1.14584563	0.633120466	0.0006299810905028420
LOC112449345	545.76989670119	1.01791333	0.731701796	0.0006326096121078400
SEMA6C	174.11335195045	-0.87056670	0.509372807	0.0006395721438800930
C5AR2	377.15950115762	0.61182832	0.223662089	0.0006507225438545350
IFFO1	608.89615694204	-0.70263322	0.285650991	0.0006626538473080930
DZANK1	373.91760993004	-0.63210949	0.173970967	0.0006674757649154160
NT5E	141.64366415193	0.65307448	0.400394759	0.0006869926585866380
WNT11	124.55987627021	0.96784936	0.353651025	0.0007078596541959380
SPOCK1	693.57358318698	-1.92487648	0.725118964	0.0007219595659687580
KCNC4	514.33139434775	-0.68978079	0.3513441	0.0007358732238368850
LCK	126.60524829022	-0.70480471	0.411826573	0.0007470967899976410
PITRM1	161.66569807578	0.73070170	0.214524966	0.0007554815115838180
LOC101903997	73.06845762444	-0.67547411	0.69750432	0.0007558306995512160
LOC521224	153.22979828146	-0.62986735	0.467982786	0.0007650776055001560
C1R	925.69042819986	0.66113766	0.588662056	0.0007845927606790790
CCDC150	583.76075868529	-0.79552259	0.364915781	0.0007859914362729270
LOC107132417	163.49685535460	-0.84716282	0.278274028	0.0007873516234604520
WDR75	612.71018554456	0.63752472	0.160408777	0.0007873516234604520
TNFSF18	114.25366738118	0.88843489	0.542880688	0.0007916057374373150
LOC112448538	635.58081882494	-0.62490613	0.671765517	0.0007957055486904120
RASD1	140.68957361234	-0.62388177	0.219038612	0.0008118215810617410
LOC104973118	222.48884078111	-1.38642644	0.515915076	0.0008517835980489550
PCBD1	719.65836394752	-0.94116734	0.42782095	0.0008764943885579450
SERPINA1	128.68646884753	-1.84613315	0.626093267	0.0008824033142713240
ISG20	344.49367513467	0.84720860	0.244559047	0.0008850313135113660
LOC107131643	861.50465442479	-0.85789683	0.687090466	0.0008920480646223640
SH3BP1	388.30163732173	-0.61468307	0.29838821	0.0008996516998114070
RN7SL1	882.27714373972	-2.45109286	0.532123748	0.0008996973827828190
STK10	348.81024299619	0.65682798	0.19261803	0.0009000000000000000

SOGA1	183.50570456110	-0.83084517	0.213523618	0.0009043614307715440
LOC112447824	207.44421475557	-0.61687454	0.313349551	0.0009510000000000000
KLF13	932.05261957494	-0.69651167	0.213631629	0.0009563151172529140
LOC112447372	914.06079685186	-0.60779411	0.324786149	0.0009588888503127090
RHBDL1	404.22391172528	0.71275763	0.375492499	0.0009681876912855190
LOC112449603	933.81113116601	-0.78839360	0.811713371	0.0009731192750397890
INHBE	557.78549992382	1.14467487	0.818744673	0.0009789796523776030
CARD10	199.12212070684	-0.81010449	0.427656812	0.0009810316851531310
WT1	155.39176775599	0.64145306	0.253338147	0.0009845159349556250
GPC2	14.66952505088	-0.98152289	0.557743665	0.0010174300065628300
LOC508153	98.38301162868	-0.71079935	0.654877207	0.0010214743325015700
LOC104973860	188.68491550487	-1.20596218	0.510862536	0.0010268155768444000
IMMP2L	893.78563747557	0.60322479	0.188638928	0.0010317628205458200
OPRM1	869.36797675846	0.77415847	0.192311136	0.0010317628205458200
RASGEF1A	113.71367291340	-0.77256123	0.573408959	0.0010408979322567100
ZBED3	219.45510486290	-0.80235886	0.249182813	0.0010649309413435300
LOC100848658	365.99401991344	-1.35833120	0.435134788	0.0010684905657285700
LOC112447804	54.28111949742	1.59127417	0.830667165	0.0010697737670074800
PTPRN2	556.35024294532	-0.77630287	0.347250719	0.0010710219194813300
LOC101904722	568.94973627534	-0.62853714	1.1147708648	0.0010897648136749100
LOC112447029	122.99610998931	1.08577876	0.3037714	0.0010959182958149200
LOC104975283	298.69193676138	-1.66381683	0.496293007	0.0011140476336299600
OPA1	913.63772242116	0.62659687	0.2083677	0.0011202825842444600
LOC112446380	287.00835642411	1.18811280	0.332909483	0.0011280586097094300
STRADB	300.68369769557	0.61144405	0.168992478	0.0011329173772944100
LOC112441843	671.91450130562	-1.90545217	0.739099649	0.0011528502768405400
HSF5	107.31968389398	1.23796105	0.528104618	0.0011871939943482300
HOXD9	697.61505414154	-0.94373887	0.793691361	0.0011975492434546300
ABCC9	100.99204740116	-0.66083243	0.379359673	0.0012334767870658100
IPCEF1	878.80728435043	0.75952065	0.191423126	0.0012396477347094200

WLS	671.76174735228	0.82456157	0.283882928	0.0012427949123470700
RASSF2	819.18260986741	-1.07687234	0.282130368	0.0012465472761965100
LOC104975941	130.17584042830	0.63719212	0.567950767	0.0012469447624848600
LOC515333	146.18443359376	0.70035244	0.256599531	0.0012544550237751100
LOC112445042	245.02131150181	-0.72826827	0.399394384	0.0012553199328042700
FRZB	950.11566709202	-1.06836798	0.307799596	0.0012600000000000000
LOC101904059	101.37156872947	-1.48357734	0.70436137	0.0012859101877788900
RASL11A	703.51344538963	1.14177077	0.292461449	0.0013000000000000000
SYTL5	103.95644918209	1.16588457	0.538271968	0.0013092030789970900
NRG2	350.94478608840	-0.97394803	0.427081771	0.0013180697503108500
LOC112444495	164.20327039066	-1.04322078	0.628563647	0.0013187693474285700
MAFG	327.53138610047	0.72648241	0.457823602	0.0013242186671226800
LZTS2	293.35735545264	-0.65546133	0.200673059	0.0013292532898005600
CTCFL	12.98382626526	0.85958600	0.581393543	0.0013325449877770900
FILIP1	454.06967702705	-0.81375940	0.311103184	0.0013443405422834800
OMP	999.89271989934	1.07527182	0.450726781	0.0013650804926245100
ZNF389	675.55316474535	-0.70639321	0.288945553	0.0013740215265592400
LOC112448421	567.57647964684	-1.21725890	0.712789871	0.0013829270950851600
LOC112449315	426.30689092699	-0.61950258	0.259156095	0.0014103423426962800
KCTD17	951.17860443020	-0.90175742	0.57198805	0.0014139124537795900
RGL3	817.44230447324	-0.66400350	0.725414847	0.0014450739870635000
SAMD11	116.26628478833	-0.61937130	0.201539036	0.0014600000000000000
RNF125	106.00591110731	0.86416534	0.256091609	0.0014673983010428400
AZGP1	106.95232474613	-1.93197849	0.662806792	0.0014971862253831900
SLC29A3	293.85726274470	-0.63423312	0.347227451	0.0015097265588724800
RPS29	491.54149416024	-0.77784618	0.201506321	0.0015214969769983500
IKBKE	6.82730808238	-0.77956440	0.247465112	0.0015300000000000000
PRODH	115.75342988929	1.13912970	0.688777987	0.0015584400999720100
LOC510613	580.18376687728	-0.69273609	0.281956539	0.0015684386165421800
RPL3L	343.60840305379	-1.21523840	1.200090242	0.0016194932352886800

FSHR	476.80287847582	-0.62889856	0.169537369	0.0016475316868738700
BACH2	30.85660378770	0.77380268	0.222930248	0.0016500000000000000
LOC112441849	737.79864660399	-1.23681437	0.681001062	0.0016675666847007200
FRK	588.65983524572	-0.79119319	0.344193963	0.0016734261335790700
YARS	147.99827000859	0.78733274	0.208882124	0.0017033121167754900
CCDC87	752.49603759633	-1.19949749	0.714215882	0.0017089440599874700
CDA	140.58317125539	-0.84914427	0.512102485	0.0017260281865889800
SLC35F1	230.90546504974	-0.74494508	0.460998757	0.0017500723790643500
RPL38	164.11087587681	-0.60556147	0.185706959	0.0017681363267940600
LOC112448059	398.73074770885	1.95257321	0.314481062	0.0017700000000000000
LOC104971292	130.97678782565	0.60104804	0.176127489	0.0017964068559473100
LOC104975091	103.65403283290	-0.67810136	0.578197845	0.0018647118722756100
GRM5	664.14170704774	-1.93402827	0.720045264	0.0019028735457683800
LOC100848721	121.02451703872	-0.74296116	0.545016005	0.0019257830290221400
GRIN2D	475.88256114513	-0.74831873	0.269010882	0.0019393205930574000
HOXD8	325.17450782560	-0.69151982	0.391406917	0.0019394290107856300
SMPD3	329.96516827457	-1.05887271	0.337897861	0.0019395089525084800
PMVK	861.08563751320	0.70735797	0.217707457	0.0019710676977722400
LOC101909003	20.92627958403	-0.91096208	0.489844712	0.0019748935132014800
ACSM1	154.52108464693	-0.81455490	0.624845337	0.0020359344278781300
LOC104969038	241.58275679572	-1.01764069	0.458944429	0.0020368443654284600
LOC104973384	40.05538492857	0.80849780	0.377583015	0.0020368443654284600
LOC112443761	164.87018735406	-0.68738175	0.456413302	0.0020997725983040200
LOC112449607	641.94749526027	-0.88424567	0.260717034	0.0021090360839436700
LOC112441463	389.25855213014	0.89710164	0.665076638	0.0021111608559677900
LOC112447811	323.69004176204	0.93425945	0.451140799	0.0021293918926232700
NMUR2	438.49872998950	1.50736860	0.711771315	0.0021302736260172000
ALOX15	139.85579244614	-1.12739200	0.599577745	0.0021601332866694300
GCKR	356.17274888955	0.69513961	0.243068415	0.0021690551640211100
CLDN11	259.09895840001	-1.06413428	0.416650344	0.0021734035905307500

HS3ST1	200.59387325418	1.20759910	0.306651331	0.0022677330458729500
TRGC4	104.24147150240	-2.26446987	0.711302604	0.0023066338706157800
LOC112442677	103.97821675285	-0.68653344	0.198796728	0.0023320614350662600
LOC112444626	441.87057230912	-2.10088246	0.970264628	0.0023597405633235100
DNAI2	552.16472919479	-0.95283662	0.326890891	0.0023826666730335200
ADCY10	100.72900236694	0.83791427	0.660824649	0.0024270877480038400
LOC112447482	728.01922027401	-1.23694085	0.670797789	0.0024275741155127200
DCC	420.84108131877	-1.09293006	0.349950538	0.0024367226458576000
RINL	819.67403108523	-1.23579783	0.666487217	0.0024708296854472000
SELP	293.05916331348	1.34437209	0.731135728	0.0024772379137502300
NKX3-1	98.73939746087	0.74325809	0.538746051	0.0024786108483711000
LOC101903383	381.31493276890	-2.21668467	1.033258533	0.0026534669216524200
LRRC15	239.87485441784	1.20155144	0.543380102	0.0026781891904099400
PXN	529.38519515832	0.68936868	0.24941742	0.0027101857607778100
IFNAR1	462.48669460696	0.64889840	0.231439147	0.0027191308438783300
LOC509911	188.23172913508	1.14331730	0.328378421	0.0027303907099336000
H2AFY2	162.09705327171	-0.74606971	0.304034799	0.0027455142500317100
TTC39C	214.15455880014	0.62784662	0.211779373	0.0027600000000000000
LOC104972026	120.12030029777	1.27001852	0.531683629	0.0027608694863174000
LOC101904622	110.82482855469	1.07705181	0.690595834	0.0028122398372633700
FRS3	202.23107700617	-1.37406147	0.464135533	0.0028402342589124900
LOC112443141	381.91312673575	-0.85372920	0.957902466	0.0028675161953292800
MRM1	103.70726674944	-0.73781168	0.364320753	0.0028764404473821400
SHF	245.59467764760	-0.92943667	0.233913678	0.0028868956290568800
SERPINC1	810.75192659708	0.60411237	0.313259884	0.0029117904799648300
KIF26A	912.56809913877	-1.03459248	0.598973188	0.0029821552697947700
YOD1	528.08709889019	0.71171468	0.400666083	0.0030242100521987300
CNTNAP4	214.62413777246	-1.03426207	0.370259339	0.0030338675220263600
APOBEC1	134.83168270745	0.88693756	1.8638738948	0.0030703860589857200
PCDHB11	342.26138538454	1.18658279	0.949710102	0.0031786707860992800

LOC104975426	681.75007427085	-1.75678788	0.819520953	0.0031998494778087600
RILPL2	514.32644018325	0.65955956	0.269681622	0.0032182382691633500
LCA5	232.00511188444	-0.71681828	0.40272161	0.0032840354228798300
TMEM232	598.93808229423	-0.61934665	0.238382338	0.0033155510518020000
LOC112444207	117.34493371822	1.07050117	0.49129047	0.0033278879373463100
TMEM44	19.91026425676	-1.03168253	0.485462814	0.0033827586321484800
YWHAZ	8.99793784466	0.68472860	0.210114169	0.0033919624932196900
CLVS2	611.91603172111	2.66373564	0.794120827	0.0034107117956964100
LOC107131798	507.88253751002	1.07894383	0.381296073	0.0034338344807762500
MIXL1	139.01201024803	-0.99287994	0.474128602	0.0034397635249864600
PKIB	209.33447605140	1.43057963	0.452930432	0.0034491252725871500
GPRC5A	197.69413785325	1.09932294	0.597055274	0.0035826205761192600
SH3GLB1	120.58736662449	0.63282505	0.155981282	0.0035949814454013800
VEPH1	249.11336779116	0.64156937	0.338514958	0.0036033882572156300
RASIP1	554.64705712536	-0.80851132	0.383147671	0.0036239456884683200
MUC13	307.33807031891	-1.51994942	0.805410995	0.0036339135419018900
LOC101908014	186.22471147628	-0.65846278	0.220473317	0.0036935695029237900
ASB15	425.43796164617	-1.27546882	0.37098227	0.0037097703811546400
INSIG1	145.62748277632	0.67724037	0.16854419	0.0037100000000000000
TJP3	349.36695362496	-1.05061958	0.425787592	0.0037440039026781800
LOC107132070	171.42098226477	-0.92773777	0.454604454	0.0038059654773855200
LOC112444532	617.59315548836	-2.50173488	0.842463409	0.0038376809569875700
LOC104973263	767.05358020503	-0.76566405	0.278889675	0.0038439340823018000
LOC787102	156.70683479649	0.70170847	0.487469325	0.0038475462923094000
CCDC36	733.30032024246	1.11450016	0.671326376	0.0038558744420964700
TIMP3	589.00765334700	0.61956365	0.195797539	0.0038700000000000000
URB2	109.35629398758	0.75973475	0.245414656	0.0039340175709483900
LOC104972734	880.25989449532	1.46036703	0.647028527	0.0039502425260841600
NTSR2	138.70562767359	-1.26767350	0.536430315	0.0039650107725831600
LOC107133073	574.07018809976	-0.95343342	0.685317266	0.0040265745239477700

LRRIQ1	886.48864576324	-1.47882937	0.616776754	0.0040341084023167300
CLEC14A	323.08576918293	0.77394984	0.508047099	0.0040547037998356900
MAB21L2	267.60797752443	-1.70183339	0.59284284	0.0041113838870345300
PVR	139.96387843019	1.50323971	0.599500632	0.0042183995121198300
SNAI1	403.29222142205	1.14302373	1.017108785	0.0042383263658582100
LOC112443742	887.34339426094	-1.41611767	0.672156129	0.0043335662686528800
TRPM5	360.08396534912	0.95101518	0.336207262	0.0043409442306876500
LOC101906392	412.44368467647	-0.70316105	0.288176384	0.0043726105584542500
GABRA3	444.10844765968	-0.75216029	0.37468082	0.0044037901536165900
LOC112446365	942.04202363496	1.45600962	0.457470508	0.0044072961436530300
DOCK10	107.44404477035	1.66119609	0.578027816	0.0044222265377545800
LOC506868	132.32225566006	0.82921352	0.670653451	0.0045711406063112900
THBS1	361.05539927603	0.69518694	0.201429393	0.0045791464544074300
FTSJ3	556.43677408674	0.61776177	0.202063411	0.0045926941233872700
LOC112448070	706.56849715805	-1.22906815	0.642305523	0.0046314979044802500
GRIA2	422.26537337705	-0.76992356	0.503116137	0.0046866567947915800
LOC112447313	189.72418620835	-1.07228069	0.423862145	0.0046909542274754000
KIAA0895L	129.03856940570	-0.70109753	0.591675369	0.0047146532613485200
PSMA8	57.52015286419	0.76263781	0.830393257	0.0047432369282448700
LOC112441682	103.75313368873	1.15265665	0.408541862	0.0047432369282448700
LOC112443236	800.67128535618	-0.85377453	0.704325207	0.0047939719532786400
LRRN4	620.47185548396	1.18078774	0.879932802	0.0048424153179899000
ZBED9	1.27165139452	-0.63891139	0.615998052	0.0048447989452207100
LOC112449267	105.11207708086	-1.90351468	0.586415239	0.0049409892445198200
LOC112444511	983.17139174205	-1.25219636	0.682057566	0.0049409892445198200
ALPL	194.77826795233	0.89381677	0.378188446	0.0049579564497523100
ARHGAP28	167.68786456809	0.69040708	0.300956381	0.0050222941436586500
SRPX	37.20367579453	-0.71902008	0.309388449	0.0050595937498024000
UBA7	323.84558916511	-0.60404360	0.527071235	0.0052196880539308600
PAQR9	178.98019107805	0.77644768	0.552607499	0.0052598937560832800

TARS	188.29795278763	0.60538978	0.171706684	0.0052600000000000000
LRRC66	570.61022957814	1.04657038	0.734305475	0.0053012044887976300
LOC112449561	354.19736766870	0.61034706	0.332797391	0.0053249301662583500
LOC107131648	111.63334070391	-0.75363550	0.548377356	0.0053439386844822700
TACR1	859.29745423664	-0.87631932	0.312026971	0.0053976957008729800
ARHGAP15	969.18327488766	-0.74188077	0.501936533	0.0055913095596383400
LOC112445105	223.85939868154	-0.90133355	0.344886065	0.0056219970398020500
LOC104970453	137.05866613661	0.70528434	0.537270532	0.0056967222013483100
S1PR5	911.93979789419	-0.74634758	0.545448251	0.0057698865073860100
FRMD4B	129.64232880440	0.75031527	0.268501999	0.0057934522092575500
PROCA1	215.33205094977	-0.80067460	0.339881667	0.0058025497763562900
C18H19orf33	740.37255458100	-0.77547446	0.50591496	0.0058171435277812000
LOC112444342	101.58415048013	-1.64585317	0.679981443	0.0058508063585241700
MBNL1	208.81147164002	0.73267445	0.186630426	0.0059553865806274900
CTXN1	134.65088877345	-0.97858049	0.472268016	0.0059650618222782100
RTKN2	560.04854422078	-0.66103556	0.259893262	0.0060596175335991500
CACNG4	303.70933776608	-2.70370670	1.234374198	0.0061287961026093200
LOC112445080	591.63135575839	0.76894197	0.713949219	0.0061452727637314000
MOGAT1	115.30061083445	-1.08144801	0.625249845	0.0061890419642423300
LOC112446026	891.58745260186	-1.24771043	0.601225281	0.0063336024049870000
IGDCC4	203.97049797536	-0.62405634	0.263242668	0.0063467587507309100
PTGDS	251.29682877327	0.80765718	0.246287299	0.0063601046512874100
LOC101907348	806.49897373235	0.63052610	0.77008445	0.0064376064366950600
PPP2R2B	904.75276705240	0.81314376	0.519316974	0.0064417209508503000
LOC112447030	151.98483814013	1.30607568	0.40799099	0.0064418278265781100
LOC112441810	221.02689942982	-0.65093935	0.393455293	0.0064544356879236900
BCL2	152.66452339732	-1.22195968	0.483146107	0.0064696841197421200
LOC112448828	160.73316472246	-0.81800892	0.645380445	0.0064761356728760800
UAP1	15.29676476239	0.67172741	0.213600311	0.0065025612767639900
LOC101902700	403.87375781207	-2.63732335	1.023670805	0.0066291635497988500

KCNJ16	19.20976769219	-1.18374432	0.60008886	0.0066487371404296800
AP1S3	111.82194416000	0.70787513	0.252772936	0.0066871941474638400
ARC	235.45778931723	-0.74378156	0.602340742	0.0067024097995699100
RPS28	611.61993614220	-0.87368136	0.222980281	0.0067130603782620000
TMEM200A	282.69134442320	0.72878164	0.264881356	0.0069400000000000000
LOC787088	145.73294242322	-0.77909703	0.466494154	0.0069652934800700600
LOC112443850	531.00660236746	2.09119662	0.833731504	0.0071806661787751200
TFPI2	188.06753158341	1.57259907	0.588964991	0.0072716073142659300
KLHL35	104.65127310421	0.77738926	0.463177307	0.0072767890412981900
TST	947.46490451714	-0.88978426	0.24871383	0.0073400000000000000
SLC30A2	568.65822863331	-1.28718157	0.673432064	0.0075035169068660300
LOC100848138	112.12651906041	0.95813999	0.589184279	0.0076101543195457700
LOC101906734	499.21766269876	1.07501477	0.853981236	0.0077502746261286700
VWA7	247.83786796227	0.92994836	0.368025547	0.0077692869598067800
LOC104975906	695.13732815792	-2.69082503	0.933052551	0.0078113885564165600
PET100	21.69035105419	-0.68229601	0.177955755	0.0078500000000000000
TM4SF20	463.44464907272	1.54729736	0.742099387	0.0078555697036527300
TGFB3	218.60349645606	-0.67083661	0.385771512	0.0078598902688821000
LRRN3	556.03163240486	1.07759978	0.285412267	0.0078630929757798300
MUC15	175.66561793181	0.83439239	0.282930028	0.0078656892193263700
LOC104973073	945.06664134631	-1.37697040	0.60054914	0.0079154935482206900
ADGRL3	352.28406448305	0.84438069	0.449197668	0.0079750759250685600
CNR2	60.41119123557	-2.01915645	0.945533815	0.0081083733614971900
ART4	840.05314550643	1.71468196	0.586520385	0.0081188878408585500
LOC101905571	716.61453011323	-0.90759768	0.270225101	0.0082725707654301100
LOC112447405	603.55051757869	-1.65761829	0.872655168	0.0085267550971180700
GUCY2C	293.00700206722	-0.98161219	0.409077247	0.0085807086442868700
LOC112448253	755.62927968186	0.64212791	0.284289471	0.0086228374500963600
LOC112449664	119.80389829617	1.26791886	0.513769865	0.0086261695831404100
PRR5L	110.89601953622	-0.88803892	0.645334362	0.0086952024709391400

ARHGAP36	552.65632209041	1.08384620	0.314480867	0.0087060033168161400
SLC22A18	552.15978957244	1.57825189	0.72138083	0.0087133774423450900
TLCD2	357.67313131898	-3.09020244	1.17571151	0.0087994264316748500
RPS27	825.81456032999	-0.76216576	0.200746559	0.0089479327934834500
LOC281376	572.91631662068	0.75138464	0.335634094	0.0089700000000000000
GRIN1	287.67820468198	-1.00259840	0.426862667	0.0090131965114677100
NLGN2	319.26987795957	-0.65010895	0.315432495	0.0090524138765263300
ZFP69B	384.29153358635	-0.68469986	0.347054676	0.0090786738449668600
CCDC8	12.38197230322	-0.66064631	0.190714551	0.0090981754257969700
LOC100337457	719.47884763460	0.79723181	0.493092446	0.0092180125888858200
LOC112441664	125.45326406175	-0.91540980	0.541193601	0.0092445487687820100
CARTPT	342.03529193366	-0.71749357	0.864196353	0.0094034892755825500
DUOX2	199.14418859266	-0.91556516	0.522537729	0.0094626889524743400
LOC112447461	214.02326117278	-0.85410460	0.389334074	0.0095084805205806200
LOC107133128	173.61096675349	-1.16641090	0.403478296	0.0095432222836747900
ANKRD55	510.04853760325	0.71918104	0.362864788	0.0095432222836747900
LRRC4C	292.20390947551	-0.60188560	0.296590236	0.0096463999941084000
LOC786352	316.28009064065	-3.40819936	1.230504658	0.0096987070277684300
RHOH	135.04747935124	-1.05457411	0.560162584	0.0097372701604116400
SHISA2	471.83679529166	0.71820504	0.773315711	0.0097565827370106400
RPS21	308.38153604645	-0.77360133	0.217689768	0.0097913072252546800
ZNF365	400.92528060418	-0.97282719	0.303365786	0.0098033484762214200
LOC112444509	112.49954132184	-1.26554368	0.548856393	0.0098168750824642600
LOC100848443	131.35138113846	-0.86520497	0.374840609	0.0098559252989378400
CTH	554.17729874324	1.13391943	0.419061756	0.0099151316250161900
DOK5	197.86143665633	0.65186810	0.494638275	0.0099221177181943000
LOC512541	596.92734647773	-0.85267510	0.70263397	0.0100044801222003000
CDKL2	152.28779538797	-0.92894874	0.473944334	0.0100432366351330000
SLC6A13	343.65590394528	-2.32106085	0.778285901	0.0100921598589843000
LOC112447791	726.42369279455	-1.63623697	0.723287895	0.0101792228401141000

LOC112443856	157.72451994895	0.82039811	0.485936387	0.0101955652487812000
LOC101908182	125.19765127182	-1.26061929	0.537166631	0.0102405070290825000
MYOT	640.49511392041	-1.10413140	0.700631422	0.0102745960862038000
PTPN6	961.39668450571	-2.19477295	0.783737854	0.0102878239021710000
SLC5A9	125.07200087911	0.86315795	0.605625236	0.0103201327489303000
LOC104975366	924.63996198174	-2.26507003	0.718091444	0.0103208541198543000
CBX7	370.79907282701	-1.22406273	0.334557843	0.0105017129890956000
NEURL1	580.08632659969	-1.28566248	1.0750164352	0.0106112033759429000
F2RL2	570.73017475453	1.33501492	0.650660597	0.0107393744288458000
LOC112441812	564.85697164462	-0.60193321	0.789286261	0.0107741339326508000
WDR36	446.01223703154	0.76560138	0.223174477	0.0108437058222081000
KCTD16	167.54890811904	0.96295268	0.547742466	0.0109307735808501000
LOC104974017	611.37163060345	-0.63516007	0.249143108	0.0111106816956939000
RAB8B	309.53591021827	0.65223098	0.214813469	0.0112208206955133000
LOC112449280	11.44674855444	-1.13733910	0.544705772	0.0113265089207793000
NMUR1	106.74809969394	-1.00350809	0.50704363	0.0113756133147399000
MYO18A	381.34769025104	-0.87023674	0.368527929	0.0114096200167123000
FBXO33	68.77876006892	0.76713609	0.224518158	0.0114307037883774000
CACNA1I	51.40607163878	0.69524725	0.799567333	0.0114699173376995000
LOC104976293	589.94475211203	1.20766504	0.808563085	0.0115196199651469000
FAP	585.14727625536	-0.74986913	0.912894475	0.0115571028130256000
SPTSSB	476.23825744912	1.97158277	0.921200901	0.0115632636106985000
CLK1	210.38799633164	0.66672312	0.230415336	0.0116450936682135000
LOC112448582	436.22452353665	0.75901810	0.224670391	0.0117213196600847000
CD274	375.43694964404	1.27400639	0.535984394	0.0118766374600464000
LOC786987	509.31139875584	-1.14792781	1.1117826401	0.0119611236602127000
DGKI	213.57828093855	0.66525081	0.394081138	0.0121173063630048000
GAL	772.71797270402	-0.83615974	0.62784263	0.0121767733369912000
JAG1	745.58224649802	0.61518904	0.328023271	0.0123019233300287000
LOC112446366	538.77600196294	0.70797998	0.588361197	0.0124098201168918000

ETV4	620.20276837393	-0.91526091	0.664211821	0.0124192917875909000
APLP1	357.93927320348	0.70337126	0.267103502	0.0125910278851000000
LOC112449665	140.75741131538	1.19054452	0.506097246	0.0128979925128473000
LOC112443864	445.46106362285	0.60879261	0.2839698	0.0129958423973234000
PREX1	362.41944097159	-1.54945804	1.10336710	0.0130428856353584000
GRIK1	442.68977824997	-0.70678975	0.367408363	0.0130919178517286000
NPR3	126.35627279441	-0.91752909	0.291371627	0.0131303547478873000
H19	545.52737584632	-1.05467601	0.762572616	0.0132496153704579000
LLGL2	125.29266353274	-0.72127301	0.501781102	0.0132666245691707000
CSF2RA	359.62271386456	-0.93373099	0.322204314	0.0133550042531644000
FAM216B	554.86220509383	1.82271717	0.92427386	0.0134528712495484000
CAPN6	326.42287297544	0.83669658	0.445355301	0.0139136850236691000
GLIS1	119.40084067968	0.83737498	0.29046584	0.0139562678124823000
LOC104969334	324.55351500780	1.21476869	0.541304626	0.0139562678124823000
STK17A	291.29508934379	0.63740217	0.230576626	0.0140742385554760000
ADGRF2	161.43302671926	1.14403953	0.484084531	0.0143412052854111000
SLC28A2	464.67465113885	-0.66571379	0.40462841	0.0143456243053854000
PRDM16	263.71804707079	-1.29806441	0.485773749	0.0144956772635627000
LOC101902469	807.92978389768	-0.94057260	0.271755497	0.0144956772635627000
LOC508879	181.78541509884	-1.41478805	0.472754013	0.0147176673565248000
LOC112446676	149.25785668005	-0.64803372	0.447571563	0.0147341600639324000
NRK	222.78705849159	0.66757094	0.433730133	0.0148087039024711000
LOC104974695	101.34946578160	0.73995632	0.539415834	0.0149211528981962000
ECHDC2	121.59761009172	-0.65345888	0.298270854	0.0149808228244418000
CCR10	563.35392116813	-1.33297351	0.891810709	0.0153927912531017000
TMEM35B	879.81898355713	0.68165804	0.609538342	0.0154165750677814000
LPAR1	728.77118194981	-0.92613498	0.741075837	0.0154952860635593000
KLHL17	692.45448546076	0.79994744	0.371476983	0.0155085092894799000
ETV5	15.45154479659	1.06815934	0.508805162	0.0155319781769757000
LOC104972031	605.79121132476	1.10806238	0.615182083	0.0157480388219132000

LOC112442996	281.45634334795	0.66475338	0.455816426	0.0157524760096339000
FAM107A	502.03708617352	-0.75009794	0.316705862	0.0157818837835973000
TIRAP	599.94919041284	-0.65707129	0.257551954	0.0158000000000000000
CSGALNACT1	314.64529409568	-1.58381661	0.411451167	0.0160511863045086000
LOC511498	417.80863921167	-1.38844052	1.074603493	0.0162181218433571000
LOC104975559	233.55960762331	-0.62482484	0.421283711	0.0164561450518203000
LOC101902647	188.70729435926	-1.58003615	0.658290348	0.0169094492284031000
ZNF853	307.02408035638	-0.67559327	0.306332419	0.0169859059107694000
C17H4orf33	113.82750463865	-0.61583315	0.513802839	0.0170067423972216000
LOC100298573	421.43311025063	-4.20251048	1.31708362	0.0171143256740036000
LOC104973105	125.52711446733	-0.96211011	0.50915461	0.0171857850139102000
LOC100847941	530.91394766687	-0.77887759	0.30893142	0.0172092357964266000
OVOL3	436.95510133729	-0.68913569	0.296545179	0.0172804051637933000
NTS	602.44670102221	-0.85837313	1.181457073	0.0173232986872222000
LOC112444864	950.43728362883	1.17262834	0.49372047	0.0174459545866966000
ITGAV	185.39265834970	0.66747893	0.200694776	0.0175189105810743000
LOC112449045	133.43588355769	-1.07349147	0.525337845	0.0177704036918526000
CHL1	6.15215676019	1.67324780	0.581238907	0.0178125454246168000
OMG	59.94737265904	0.92561880	0.699369141	0.0181398151654661000
LOC112441942	15.42098852657	0.81239575	0.380000139	0.0183905581327347000
ATP1A2	594.91333596232	-3.82046660	1.250816785	0.0184387513732152000
CRYBA1	114.09756369543	0.63278232	0.552326044	0.0186332911377378000
PRELP	295.00273662549	0.63600771	0.346330718	0.0186508061842527000
FAM84A	856.87607866765	1.32001513	0.917817647	0.0186835694672759000
LOC101906363	167.25040062057	-0.61888779	0.213198191	0.0188534569156249000
REEP6	110.34577821151	-0.76899785	0.491741836	0.0190909799342056000
LOC112448496	358.80336468029	-0.61864280	0.166353806	0.0191000000000000000
LOC112442378	37.05365751675	-1.18387346	1.178164577	0.0192527075894068000
LOC786139	363.96009161004	-0.93278754	0.398568479	0.0195870579682734000
FAT2	289.02734490930	-0.61877370	0.381269081	0.0196560987254148000

LOC101906531	152.71178083838	1.09687285	0.551523148	0.0197731433149470000
UBXN10	111.07228897913	-1.13550516	0.681304449	0.0199878277389670000
LOC107132787	179.74400305577	1.27217977	0.569819019	0.0200274179738576000
LOC112447027	628.21987899073	-2.00444859	0.977944475	0.0200558215387690000
CILP	153.26722170066	-1.06048307	0.580666423	0.0200991992194925000
LOC112448904	437.36909695900	0.74739685	0.75089542	0.0202272439587165000
SLC24A3	556.59465533636	-1.18317179	0.755223209	0.0202673182769708000
LOC107132832	39.28265471267	-0.96366092	1.150002010	0.0204573313735758000
BDNF	523.72646334080	0.83801318	0.31484107	0.0205483998642182000
LOC107131944	207.81400868076	-1.58675595	0.504872846	0.0206336405393349000
CA5A	128.96998189418	-0.82345270	1.18578958	0.0206563352352512000
MYH1	412.17868028811	-1.33283381	1.032585657	0.0207718869145224000
ACP4	646.71772299583	-0.65199993	0.238304145	0.0208251764394710000
LOC101907487	726.48958338308	1.13928502	0.54694676	0.0209498963241173000
ZNF2	116.50290759428	-0.75485023	0.300086985	0.0210000000000000000
ANKRD9	313.59714932204	0.60113003	0.274211951	0.0212470120798598000
ANO3	182.37256828135	0.71509228	0.232648773	0.0214481022564641000
SYT3	790.93592908787	-1.21121587	0.702387366	0.0215566945707605000
ARHGAP8	343.31746165857	-0.83708588	0.472015148	0.0215568407473581000
NR2F1	362.91544610237	-0.91113064	0.325096551	0.0217638332630419000
SHISA9	109.31084625974	-2.51081358	0.696131415	0.0218615325651679000
LOC617565	119.82630091703	1.06178742	0.556900883	0.0218615325651679000
LOC112442803	808.91088131478	0.85581831	0.513824224	0.0224761822252780000
UTP20	434.22346321530	0.75967804	0.235648276	0.0229001915401697000
SMAD6	119.75258157695	-0.78144263	0.696564391	0.0229334605305183000
FSD2	136.19022652525	-0.61390400	0.221066307	0.0230482887107350000
FAM19A3	851.17032617862	0.78828436	0.7035038	0.0230854076812756000
POLN	537.80125097049	-0.80068838	0.283494123	0.0231910654125197000
SCN10A	65.58681057912	0.72831107	0.231024999	0.0234047792884894000
MIR1842	166.94274487318	-0.74299880	0.367510115	0.0235380618463553000

LOC100848226	166.88940567401	-0.67026011	0.431811752	0.0235519850673382000
TGM5	531.21008545024	-1.72397247	0.845645934	0.0237370436090974000
MPP6	601.18110637472	0.64032449	0.189970459	0.0237979662068053000
LOC101905075	57.68154383196	-0.91639123	0.75719929	0.0238983835533015000
LOC101907142	539.64659442235	-0.89009309	0.786091403	0.0239378812524133000
GALNT15	109.77308719835	-1.76952760	0.730033202	0.0239979714006176000
LOC101904601	233.49839505479	0.75283324	0.449756225	0.0240431202501311000
LOC104972409	169.97935402851	-0.64347721	0.388187524	0.0240913755387839000
LOC100140130	872.93349113300	-1.54242033	0.879124733	0.0241682850150855000
LOC100848752	29.34310158972	-1.23503218	0.494031979	0.0242568653837306000
NOBOX	879.50795479660	-2.20777734	0.740242559	0.0245227414824893000
SS18L1	51.64042999138	0.67239484	0.296658781	0.0245835152671019000
ASAP3	108.78923171513	-0.69464145	0.626742019	0.0247770679574621000
RNF133	163.00639338395	1.12910096	0.383695219	0.0248313745726945000
HMGXB4	221.29169369799	-0.62501482	0.197203128	0.0249028992471618000
CFAP58	944.09834200420	-0.69056775	0.566117522	0.0250283674289728000
LOC112443214	499.39460509796	0.89039042	0.725954627	0.0252627771359885000
ALPK2	256.70626969496	-1.00045891	0.467602615	0.0254361223919196000
NT5DC3	886.40860464364	0.64694281	0.208373815	0.0254518361131494000
KIF26B	367.82778728166	-0.70815692	0.328881499	0.0254879394862848000
LOC101904822	578.90991824186	-0.73178633	0.688281851	0.0254999511150480000
LOC104969934	134.87115039987	0.67381325	0.227424811	0.0255282092603059000
E2F2	760.29936843149	-1.34381297	0.707027612	0.0256459685780776000
PRSS53	4.51251675191	-0.82755044	0.317534515	0.0256459685780776000
LOC104972569	341.30336338388	-0.63873976	0.310687938	0.0257145198558012000
LOC784052	295.35157104448	-1.38348184	0.418132714	0.0257530336560575000
LOC112444614	16.65958115929	-0.86315017	0.410178856	0.0259695753230447000
STIL	49.66493690342	0.80465453	0.343550421	0.0259973617532358000
MICALL2	268.45186829963	-0.69983751	0.523358518	0.0261675092915630000
ADAMTS4	231.13062295238	-1.35471450	0.842890882	0.0264277578004684000

DTX4	140.01619620394	-0.79474098	0.49325177	0.0265021998886812000
LOC101906171	156.00853847140	-1.35295933	0.470271097	0.0265024897909823000
ADAMTSL1	174.86162770627	-0.87939042	0.419340315	0.0266468700740172000
CYP1B1	128.98238096696	1.33009689	0.467137997	0.0266897025295256000
LOC112445938	316.84892848643	-1.04729452	0.316719262	0.0271937574821196000
LOC112445758	426.15997227251	-0.78375034	0.984818444	0.0271937574821196000
LOC100847981	757.75771549519	0.73851161	0.590197404	0.0273832959555526000
LOC101905905	857.57827161348	-0.87391117	0.541070772	0.0277839895506131000
IRGC	513.34158422395	0.81527796	0.834082922	0.0278061442751568000
GRO1	302.56807225617	0.92540187	0.502482652	0.0280393937768290000
EPHA1	474.56841276301	-1.70803594	1.141447679	0.0280632586747500000
EXD3	403.86645725790	-0.60858117	0.299833628	0.0280810802231082000
IL3RA	256.60152531582	-1.11087107	0.354494776	0.0281644404639896000
NUP205	132.69492613929	1.19203545	0.201392846	0.0282000000000000000
LOC107132344	851.11834750404	1.27512690	0.496165591	0.0282426892531723000
AFMID	235.54631106405	-0.82821174	0.393514887	0.0282544577323680000
ZNF385A	177.88773696817	-0.75622026	0.481612603	0.0284379944506793000
FUT6	925.76565824127	-0.80582856	0.649134071	0.0285684955314048000
ITGB6	252.67792939850	-0.65091523	0.224638445	0.0288444954177618000
LOC101902930	98.77872226328	0.61479736	0.486574989	0.0288698661323899000
LOC112449036	776.28294495675	-1.77721245	0.649211586	0.0290121921796784000
LOC101904698	884.92428572093	-2.03266805	0.773505185	0.0291281425383838000
KCND1	488.58617112370	-1.04916199	0.365967941	0.0291869936590092000
LOC507550	816.91534354958	-0.89702422	0.588171872	0.0296025895773446000
GPR18	144.56126590499	0.89615249	0.527774094	0.0296370617880226000
RBFOX3	291.32775990161	-0.68058622	0.368574855	0.0298336933789964000
OTOL1	126.95007835205	0.75767877	0.502418869	0.0298450016969151000
MAP6	634.84601923158	-1.22018145	1.068796413	0.0299058989927775000
LOC112445660	455.87109628990	-1.85601817	0.855087707	0.0299724559512379000
ANK2	186.79814657356	0.60868620	0.240700778	0.0300463039246760000

MYO7A	600.53231422086	0.89878004	0.464472514	0.0305610252036794000
SELENOP	201.27693223330	-1.02650128	0.413280001	0.0305744592862658000
LOC112448846	168.32673030913	-0.65231268	0.408840547	0.0308684173258004000
HAVCR2	430.35200192215	-0.78236678	0.332416276	0.0312339234281891000
FNDC4	229.03028108415	0.83523396	0.336183927	0.0312339234281891000
FAM199X	259.38346882311	-0.61921413	0.403726589	0.0316756978323347000
CYP4F2	809.97770360859	1.22460224	0.689669708	0.0316870581446562000
LOC101906077	502.09214309780	-1.87469661	0.849492002	0.0321850194331150000
SLC6A14	153.78875510189	-0.63188692	0.189311358	0.0323120357429620000
GJA4	122.62839718089	-1.46538330	0.56991329	0.0324998266716443000
FOXN2	853.52293981734	0.66807961	0.30059784	0.0326455923691663000
PTPRE	197.44691769194	0.70437185	0.474445889	0.0327687545248997000
LOC112444750	463.38477839772	1.42133365	0.719352932	0.0328089789880983000
ATP6V1C2	472.98415178423	-0.63512779	0.231139076	0.0332197482071933000
ATP9A	104.45618988751	1.32199558	0.558639572	0.0333495887071462000
CDH16	470.76767172144	-1.16881194	0.80126876	0.0334945366151962000
ADAM33	11.95983553997	0.88797765	0.608496652	0.0334945366151962000
LOC100849067	269.72177053470	-0.72206517	0.358249072	0.0335257548315267000
MSX1	773.74051867586	-0.88988770	0.564958412	0.0337254385944989000
ZNF157	466.87320048637	-1.53343795	0.92873764	0.0338048946079590000
CHAC1	265.68979772544	-0.64172170	0.238437705	0.0339410396189994000
LOC112447378	691.99698503967	-1.26935057	0.762691851	0.0339443934769502000
TRAF5	19.28326300834	0.72317765	1.359192593	0.0342433925120391000
LOC104971069	103.41670549278	-0.69654749	0.636550693	0.0346483592205938000
PTX3	211.84777422703	0.81789399	0.490906062	0.0346577396643751000
C19H17orf100	177.37684411492	-0.92875374	0.492512066	0.0350678102018796000
SPRY3	159.69161897062	-0.82281002	0.436089346	0.0352392293948489000
HOXA4	332.68717792793	-1.19154962	0.82466068	0.0356409273986588000
MATN3	613.00176488916	0.79500190	0.385924431	0.0358832344251957000
LOC104973489	609.27334079807	-2.42527080	0.830220323	0.0358990130213817000

LOC112446500	116.07922202478	0.62926383	0.390889946	0.0360108779368636000
CD200	447.60722668696	1.18852284	0.385153793	0.0364839859930222000
SSTR1	844.16230982281	0.84832526	0.400128797	0.0366882290043687000
BSN	171.83481065796	-1.29440903	0.444046328	0.0368553604304283000
PTPRR	134.43336352137	1.56089998	0.616831256	0.0369323802634700000
PSD4	20.04966730600	0.97417510	0.671253734	0.0372485165171002000
AP5B1	142.85736159775	-0.60424078	0.207451336	0.0373000000000000000
FOSB	20.46405330106	-0.82566022	0.474891703	0.0373786785288862000
CACNA1D	714.79227455492	-0.84607104	0.533318022	0.0375058275494476000
LOC104972906	562.10755418427	-0.76454760	0.715241753	0.0375852643471285000
LOC112446358	11.80369000942	-0.85578108	0.534223939	0.0379767705245621000
TBC1D25	340.47686995648	-0.66398347	0.242752353	0.0380300253722459000
PLVAP	659.81631631434	0.91683274	0.890259067	0.0382738424764627000
TMEM182	401.08194402484	-1.00602050	0.932490366	0.0384740269271368000
GPR156	638.31692414778	-0.98679176	0.829989337	0.0387567523155949000
CORO2A	612.73026678724	-0.97245645	0.731881396	0.0388704104365259000
NAIP	104.45477453117	-1.12819772	0.55909464	0.0392446114434894000
ST14	136.18853370621	1.34331390	0.466443713	0.0393193749244584000
SLC1A3	109.00438744289	0.94188860	0.662424907	0.0396333977890585000
OR11H6	690.01660530511	-1.35797934	0.9892081	0.0396907496370956000
TMEM130	129.96752165452	1.41664550	0.677790897	0.0399122151738227000
LOC112448057	792.61505662023	-0.77926989	0.634157856	0.0405125909371198000
RPL37	809.02496614972	-0.64167157	0.199487672	0.0405684565703933000
KRT10	315.82527535117	-0.91370569	0.334766485	0.0407990363172252000
ARSI	345.45064858928	-2.45338773	1.194235791	0.0409587834498227000
LOC112443766	483.00999736757	1.39708151	0.756015753	0.0410074238209430000
CDH4	104.24768841634	-0.74292469	0.556648036	0.0411286249491538000
SGIP1	32.77893572069	-0.87891367	0.422285378	0.0412070991468064000
SEMA5B	576.60710936719	-1.43202477	0.814446691	0.0413049327716439000
LPL	159.28934226916	0.71957810	0.263553942	0.0416176276058107000

LOC101905966	668.86288875374	-1.30566009	0.613662224	0.0416500546688818000
METTL21A	332.34835190517	0.61507990	0.199311683	0.0417194691994017000
SLC7A14	488.32981319464	-1.85847230	0.924109551	0.0418780574998691000
LOC112444643	675.27249508016	-1.05860087	0.611618366	0.0419367286675848000
NHEDC1	102.24374917675	-0.79085692	0.504284761	0.0419512973787456000
LOC112448792	121.19761076213	-1.00585477	0.49223775	0.0419943367902457000
LOC101906339	139.25033312659	0.93658359	0.314002269	0.0422483024290261000
LOC104969916	553.65117887601	-0.94296273	0.776092659	0.0423523526955603000
PIGZ	526.11817383101	-0.73654495	0.71342417	0.0425189317419449000
GRASP	28.13286376121	-0.83071979	0.370482786	0.0426749587184916000
LOC112446054	148.41092096396	-0.64603257	0.510680733	0.0436989995085428000
ZNF232	207.19357972817	-0.65631664	0.393990063	0.0437922895660409000
TPRA1	515.75592590567	-0.69435359	0.288128252	0.0443414318270287000
EXOSC6	3.05936579954	-0.60790901	0.376559561	0.0444994163277675000
NPY5R	316.17681244015	0.68763436	0.265530055	0.0445777720086930000
GGT5	819.96282556243	1.13450394	0.616952706	0.0445939879528860000
C2CD4B	392.37884340313	0.76016257	1.044005496	0.0446158143211749000
TRNAG-CCC	122.52952572648	-0.61829710	0.454335713	0.0451493867402307000
CD160	845.54697820734	-0.61983705	0.525134453	0.0451940495924318000
LOC107131779	148.75298167512	0.88533401	0.502369753	0.0455125362183586000
GGTA1	183.65924259109	1.27534069	0.503792945	0.0458691714692268000
LOC112449243	62.26988548535	-1.61566507	0.726945036	0.0460008229925044000
LOC100848689	429.28972119243	-1.65655911	1.293967468	0.0460286448087033000
LOC104973551	229.15148711052	0.68728204	0.226013965	0.0466632180910261000
LOC104970778	669.00360258900	-1.73010396	0.933093106	0.0467322677128319000
SPATA13	1.58481891508	-0.98354247	0.565951879	0.0467754704504581000
LOC101906006	881.13443793363	-1.37668132	0.639617678	0.0468699831160638000
LOC101906850	122.26025378204	-1.03831831	0.435376563	0.0468925437883035000
DAPP1	613.64068028026	0.73063046	0.311664242	0.0469026062479218000
LOC112449260	90.67914671757	-1.46558829	0.6206279	0.0472888910740858000

LOC101904714	890.82172342686	1.33675335	0.599327594	0.0474171595367502000
NR5A1	625.21583254672	-0.61297050	0.24800789	0.0477590394734935000
LOC104969611	289.45714644538	-0.71014563	0.358233577	0.0477605157022375000
GPR22	725.60195124644	-1.49380329	0.735008447	0.0477931969919191000
DHRS13	559.27974015675	-0.60941661	0.777352733	0.0478125981713748000
IFIT2	431.35309180539	1.24281561	0.836396936	0.0479386730251562000
LOC101909384	101.62942201008	-0.86180834	0.622705513	0.0481159079192665000
WDR72	30.46290457944	0.74452728	0.344981139	0.0481807665775424000
GJB3	210.80045397180	0.98036519	0.398357458	0.0482046792013210000
SLC25A35	736.80744984243	1.08574726	0.786137905	0.0483132787493006000
LPIN3	313.78302685043	-1.04178410	0.352383367	0.0489683098104334000
GAL3ST2	337.74070715461	-1.76113804	1.277888517	0.0491090398622787000
PDE11A	321.70843883598	-0.89163659	0.377684708	0.0493618310652245000
TMEM38A	236.23824966212	0.61292722	0.302733272	0.0495038750259042000
LOC104969002	191.70379813986	0.90233717	0.458885126	0.0496531997477467000
LOC112447547	757.58479728945	-0.79274636	0.591749773	0.0496880344029758000
