# Supplementary material for: Extracellular Vesicles Derived From Antral Follicles Significantly Change the Transcriptional Profile of Cumulus Cells and Oocytes During Pre‐In Vitro Maturation in Cattle
Source: Mol Reprod Dev. 2025 Nov 24;92(11):e70068. doi: 10.1002/mrd.70068 (PMC12645189; doi:10.1002/mrd.70068)
Supplement: Supplementary file 4 — Table S3: Differentially expressed genes in cumulus cells of Early EVs vs. Late EVs. [file MRD-92-e70068-s005.pdf]

Table S3. Differentially expressed genes in cumulus cells of Early EVs vs. Late EVs.

Gene	baseMean	log2FoldChange	lfcSE	padj
CPNE4	779.6770701	-0.674457189	0.221263565	0.0000000000000000000000000
CDR2	145.4589747	0.661896567	0.217289193	0.0000000000000000000000000
FZD10	45.70169953	-0.895045361	0.243653945	0.0000000000000000000000000
ELAVL3	17.72183512	-0.908037303	0.342554993	0.0000000000000000000000000
TMEM100	57.67633366	0.696196049	0.301794757	0.0000000000000000000000000
AK9	66.61660531	-0.663295866	0.195027697	0.0000000000000000000000098
ZNF215	175.4522311	0.800209212	0.189559469	0.0000000000000000000294592
TRIL	234.3714958	-0.667446307	0.225556855	0.0000000000000000012066074
MDM1	103.9327294	0.629630347	0.207360377	0.0000000000000000027491627
RIMS1	186.0634266	-0.634049514	0.191680734	0.0000000000000004380518242
NGF	278.3650176	-0.658449516	0.247311794	0.0000000000001647019413773
LOXL4	117.6393448	-1.182395623	0.413892393	0.0000000000020485940364797
ANKRD1	144.5003608	-0.84143412	0.305598883	0.0000000000110981724552243
MMP19	61.0197056	-0.849373735	0.284664991	0.0000000000655053863233221
DQX1	24.39030974	-0.77335463	0.350957701	0.0000000001056964083635230
GPM6A	61.24057807	-0.666653766	0.221289782	0.0000000001675408504838180
CNGA2	5.94715472	0.844576974	0.907183735	0.0000000001733485776495890
MT2A	2752.019985	-1.426152019	0.508478616	0.0000000001874345765288650
IGSF9	38.43893337	-0.601731657	0.351941867	0.0000000014790095469065200
AXL	336.2914946	-0.606662721	0.237503877	0.0000000018211541781055500
EFNA4	47.84343484	-1.181169663	0.247325999	0.0000000037818699701605100
GPRIN3	179.8204586	0.73273401	0.239322848	0.0000000046928749294100400
PLEKHH2	131.3837507	0.639020546	0.195409583	0.0000000063316180046350500
NR4A1	57.87182995	-0.861321899	0.327217642	0.0000000071428531242148300
GADD45G	159.6953678	-0.672105389	0.24203682	0.0000000077682133344921500
AVP	60.50319373	-0.656019052	0.204233163	0.0000000107676904628411000
ABHD1	117.9806413	-0.64177004	0.227888304	0.0000000539160288648420000
MXRA8	852.6081679	-0.69480396	0.243941622	0.0000000548181782831568000

ZBTB8A	26.73518662	-0.796666919	0.278678738	0.00000000071131786326890700
TFCP2L1	897.7903544	-0.650255804	0.19528219	0.00000000104669716126775000
PECAM1	58.53737581	-0.687318719	0.349411644	0.00000000159368119306335000
UNC5C	37.81985111	0.756590549	0.288369564	0.00000000208050551758971000
THNSL1	42.0504893	0.742588005	0.221512743	0.00000000300116233880285000
TPD52L1	97.22184	-0.790351902	0.22766498	0.00000000305486207223791000
MT1A	2197.038397	-1.507365452	0.664959277	0.00000000366043956291605000
DLL4	248.3088061	-0.61399298	0.258219883	0.00000000436960472733706000
CPEB2	59.1331936	0.601813116	0.236475217	0.00000000664525440049942000
SEZ6L	7.211433571	1.719792798	0.799175746	0.00000001718355391836800000
REL	30.29692686	0.663349744	0.304889416	0.00000001873386154669710000
BOLA-DQA2	6.915497181	0.814873797	0.659256139	0.00000002727722217655400000
XKR8	70.78460417	-0.825011245	0.208065649	0.00000002845619200552430000
PLEKHO2	138.4622545	-0.731740307	0.154586704	0.00000007566540611021530000
ZMAT4	18.39187096	-1.005906754	0.373774532	0.00000009821053863436510000
BAALC	10.2642341	-0.767863355	0.556510728	0.00000010714659077647800000
WDR49	6.024542695	0.682675714	0.625856191	0.00000011557806198827400000
MEP1B	286.4832872	0.721457388	0.237608496	0.00000014482871947196700000
ZNF19	14.58320578	-1.276786511	0.383055022	0.00000016601205023973800000
RORB	17.02698158	0.654028206	0.303903022	0.00000020059155386092900000
KCNT2	9.671040363	0.786800691	0.379621918	0.00000023711704602123100000
BCO2	11.77432229	-0.72694685	0.40641658	0.00000024545829978312200000
PTHLH	9.357292411	-0.864181775	0.419309217	0.00000026514134670684600000
CARD6	26.13623225	-0.767431246	0.316808977	0.00000028900327816607000000
ABCB9	17.52838163	-0.650667984	0.323155573	0.00000035819421565016300000
STC2	4.669135861	0.8147439	0.747345392	0.00000038166968708655800000
IL12A	18.25566156	0.77430743	0.367909565	0.00000051399360975824800000
FSHB	380.2531428	0.608084446	0.26770759	0.00000075550670755410200000
TNFAIP6	306.7021526	-1.326639049	0.605294917	0.00000118093966345868000000
FAM167A	23.31888024	-1.027301373	0.435680203	0.00000140051629794919000000

NTRK1	28.39189942	-0.912819896	0.61760477	0.000002082154315252870000000
ADAMTSL3	48.26186429	0.694219698	0.315344034	0.000002625385452112170000000
MYT1L	21.02044287	-0.787233055	0.344098169	0.000003365692217015470000000
TNFRSF6B	381.5144214	-0.637562491	0.200301462	0.000003501873987019830000000
MT1E	3442.47449	-1.38738859	0.696361321	0.000003821744965744620000000
AKAP3	13.57719115	0.852588187	0.396201093	0.000004119571665573040000000
FBXL21	168.8196848	0.626202292	0.185091485	0.000005333908933321700000000
PIK3CG	7.391006023	1.648908718	0.664343701	0.000005390942557464290000000
SDC1	100.5944072	-0.971677254	0.264392658	0.000005738739837620370000000
ZBTB16	30.52372408	-1.621800814	0.406453851	0.000006443201943299540000000
KCNH8	110.080985	0.7285264	0.428316313	0.000007680184055305520000000
JPH1	30.64726863	0.744204529	0.294436242	0.000008249283334087400000000
IL7R	26.28421166	0.851072636	0.354476934	0.000008299029143851110000000
CD40	33.385804	0.74685038	0.341940161	0.000009102678444840600000000
MMP23	9.813667132	-1.291426284	0.491568678	0.000012378096363125000000000
KRT18	1253.360731	-0.808243724	0.230220583	0.000013868495057146300000000
FATE1	49.01956605	0.923051323	0.33252535	0.000015522763118863100000000
KIT	212.0501299	-0.609226337	0.268105526	0.000017218898434920400000000
HPS6	102.7503051	-0.723461809	0.304284676	0.000020384484583877300000000
SGK1	61.91212529	-0.855856572	0.349956154	0.000026009128308775300000000
FAM162B	26.52546387	-0.860908998	0.47745037	0.000026635994528151000000000
TAAR1	38.90521699	0.677524942	0.320725412	0.000028888688880384500000000
WFDC2	43.79750378	0.618109462	0.386096467	0.000028968724961089800000000
TEKT3	11.09139221	-0.905579031	0.535965375	0.000030739717282354300000000
LURAP1L	15.24885782	-0.68000506	0.455645295	0.000032148572446694100000000
ADRA1A	16.3853371	1.189987204	0.409235834	0.000034389530433406600000000
PJVK	21.678302	0.7959889	0.289973761	0.000039166304042791000000000
ENTHD1	9.173470356	-1.010821449	0.547346936	0.000044278004931601000000000
REG4	12.1806795	0.630371481	0.452935205	0.000046290510347545600000000
SPATS1	10.31406968	-1.277772237	0.447350947	0.000049171565453586900000000

PRSS50	17.84867047	0.676057183	0.427882777	0.000051905799647786800000000
NBEAL2	43.76474887	-0.678903901	0.219205057	0.000056032130289297400000000
GBP6	11.55501364	0.715874766	0.421190898	0.000064619238775765600000000
ETV1	33.35397824	-0.854052676	0.312366411	0.000065793353067382900000000
CBX2	34.12228366	-0.81029372	0.339157662	0.000073959974983807500000000
TRIM50	17.49767371	-0.748835521	0.372802769	0.000079870920950122300000000
P2RX7	8.93024265	-1.294775261	0.502891948	0.000110394920775101000000000
OPCML	12.16202593	-0.668326115	0.374169156	0.000116965416899575000000000
FGFR4	13.36260714	-0.68286025	0.473919479	0.000146339388536898000000000
PRRG2	7.929766155	-1.378536767	0.474003086	0.000156598299577952000000000
GSN	1741.44557	-0.780484429	0.231387088	0.000159873152536363000000000
HPCAL4	4.882997424	1.127330693	0.795316685	0.000218206376593580000000000
VIPR2	10.24282755	-1.902773683	0.528615492	0.000222127809271390000000000
GRK6	95.49803256	-0.661459005	0.224307745	0.000236230097465372000000000
OGDHL	6.024840153	-0.611792936	0.812317543	0.000236751728725151000000000
C15H11orf42	14.54961036	0.92473191	0.42340777	0.000249280685950019000000000
PPFIA4	26.82824462	-0.707371021	0.288856275	0.000283849287891809000000000
CPT1B	55.23747944	-0.659377152	0.284562791	0.000295317873443148000000000
ANKRD34B	5.266309744	-0.895134287	0.62510809	0.000298194440477578000000000
MAP3K9	40.18643827	-0.605908335	0.235136749	0.000330289208917010000000000
MFSD4A	22.59336367	0.666728062	0.35347585	0.000341250558852415000000000
CACFD1	346.4968914	-0.609527992	0.221691254	0.000364827726244613000000000
PLAUR	841.536716	-0.617191165	0.2439394	0.000388167886700359000000000
FAM205C	4.203078825	-0.757031954	0.895067579	0.000408135065743208000000000
GDAP1L1	9.278992332	0.673205923	0.548859914	0.000471805230368623000000000
PDE4B	31.66451724	-0.874513193	0.42517441	0.000474612476474173000000000
C25H16orf45	7.67278718	-0.989829106	0.585756335	0.000480158712594116000000000
TRPM2	4.664210609	-1.023491579	0.819909221	0.000538648934506148000000000
FBXL6	145.6520288	-0.655892225	0.173297589	0.000582211713736308000000000
CDHR5	5.742027321	0.878613643	0.635964479	0.000611043709852599000000000

ANGPTL4	119.4400979	-1.068555237	0.514792154	0.000629981090502842000000000
SEMA6C	17.4113352	-0.925822718	0.402902199	0.000639572143880093000000000
IFFO1	60.88961569	-0.667553801	0.227437953	0.000662653847308093000000000
MGC157082	5.261937086	0.657149695	0.806390169	0.000673960742764229000000000
PDXP	48.60839172	-0.61081282	0.353860215	0.000685474881153738000000000
LCK	12.66052483	-0.897920161	0.32347624	0.000747096789997641000000000
TNFSF18	11.42536674	-1.057237709	0.47861608	0.000791605737437315000000000
SERPINA1	12.86864688	-1.590432689	0.478001754	0.000882403314271324000000000
RN7SL1	88.22771437	-0.758028384	0.417821635	0.000899697382782819000000000
CARD10	19.91221207	-1.394441147	0.347969497	0.000981031685153131000000000
LNX1	63.88615477	0.63697724	0.252503923	0.001005082134971230000000000
GPC2	14.66952505	-0.710443734	0.432263916	0.001017430006562830000000000
BOLA-DQA5	5.244871578	1.205242982	0.737681779	0.001026850174788240000000000
GRB7	10.84771052	1.14403413	0.470035308	0.001043907212619460000000000
PTPRN2	55.63502429	-0.630387649	0.275152626	0.001071021919481330000000000
PDZD7	3.348813705	-1.167974605	0.963904532	0.001112325731987180000000000
GABBR2	17.24499996	0.734345317	0.432688431	0.001173276141219630000000000
HSF5	10.73196839	0.695090189	0.440220326	0.001187193994348230000000000
RASSF2	81.91826099	-0.684504842	0.222259743	0.001246547276196510000000000
FAM110A	23.29425517	-0.684949031	0.266122241	0.001297894776285070000000000
SLC29A3	29.38572627	-0.705047855	0.276017121	0.001509726558872480000000000
RPL3L	3.436084031	1.727368582	0.792528321	0.001619493235288680000000000
CCDC87	7.524960376	-2.083438619	0.596976781	0.001708944059987470000000000
CDA	14.05831713	-0.818665755	0.404259103	0.001726028186588980000000000
GNAT3	13.0758738	-0.759231822	0.515574713	0.001772392823347850000000000
GPR146	31.41383703	-0.754671345	0.349092816	0.001872102209827560000000000
FRAT1	40.29120185	-0.64188563	0.309314869	0.001892311130691800000000000
CD24	51.36311882	-0.828849559	0.505176236	0.001910062864496130000000000
ACSM1	15.45210846	-1.168536597	0.501573554	0.002035934427878130000000000
ALOX15	13.98557924	-0.818407529	0.465079827	0.002160133286669430000000000

CLDN11	25.90989584	-1.306427976	0.332266879	0.002173403590530750000000000
TRGC4	10.42414715	-1.6531859	0.523158217	0.002306633870615780000000000
ADCY10	10.07290024	0.869820345	0.534409304	0.002427087748003840000000000
ISG12(B)	32.12208344	-1.106471855	0.267890311	0.002566578514249890000000000
SPATA46	15.22902345	-0.887967752	0.398369123	0.002635172104262470000000000
GABRR3	8.161065151	-0.647669331	0.443734586	0.002640975985460160000000000
GAB3	32.15464496	0.736337133	0.297665899	0.002679781231399940000000000
H2AFY2	162.0970533	-0.85135293	0.246372651	0.002745514250031710000000000
UNC5B	32.28485155	-0.909355391	0.440626187	0.002826470941373680000000000
FRS3	20.2231077	-0.738262745	0.349423458	0.002840234258912490000000000
RAB38	158.6560474	0.747448501	0.409909088	0.002870330859183080000000000
KRT14	5.474123178	-1.195681527	0.63656915	0.002874111384915910000000000
MRM1	103.7072667	-0.660379056	0.294529879	0.002876440447382140000000000
CXCR6	10.41495083	0.716957142	0.497372902	0.003036305467776300000000000
WWC1	7.597173528	-0.728090553	0.476282111	0.003188542027703190000000000
TMEM44	19.91026426	-0.603452087	0.374582203	0.003382758632148480000000000
MIXL1	13.90120102	-1.02885328	0.370718668	0.003439763524986460000000000
GPRC5A	19.76941379	-1.191117569	0.510538786	0.003582620576119260000000000
CCDC36	7.333003202	1.403200033	0.542492489	0.003855874442096470000000000
KRT17	7.664249407	-1.431725773	0.740587513	0.004091841059931090000000000
SNAI1	4.032922214	1.162977545	0.832449184	0.004238326365858210000000000
ADAMTS2	6.081613587	-1.412935461	0.628053237	0.004275284234774570000000000
TRPM5	36.00839653	0.804322129	0.274250966	0.004340944230687650000000000
KIAA0895L	12.90385694	-0.823242451	0.47354104	0.004714653261348520000000000
PSMA8	5.752015286	0.621318495	0.674370667	0.004743236928244870000000000
LY6E	3.290638047	-1.284393267	0.942775643	0.004816161388884370000000000
LRRN4	6.204718555	0.872037965	0.724919612	0.004842415317989900000000000
GPR135	9.213674053	0.815136545	0.467747923	0.004862998212081880000000000
SRPX	37.20367579	-0.770901267	0.244357031	0.005059593749802400000000000
PAQR9	17.89801911	-0.720178312	0.469208557	0.005259893756083280000000000

LRRC66	5.706102296	0.909132444	0.60121292	0.005301204488797630000000000
FAIM2	21.73432295	-0.659015736	0.415939301	0.005394201816325300000000000
EPHA7	33.04864121	-0.665681738	0.308007743	0.005404937208601250000000000
KCNE5	25.20382365	-0.739361938	0.477777839	0.005636990658223010000000000
CYP17A1	14.06432908	-1.140229342	0.522850836	0.005679920370564620000000000
SLITRK6	12.35262404	0.730839865	0.439262491	0.005816225076682160000000000
C18H19orf33	7.403725546	-1.555634139	0.423481204	0.005817143527781200000000000
CTXN1	13.46508888	-0.650819267	0.359266538	0.005965061822278210000000000
CACNG4	3.037093378	-1.18288437	0.84682676	0.006128796102609320000000000
BCL2	15.26645234	-0.917304984	0.367176787	0.006469684119742120000000000
MLYCD	111.5601556	-0.692781498	0.293933835	0.006469999343346280000000000
ARC	23.54577893	-1.765287864	0.496339467	0.006702409799569910000000000
ANK1	45.53910646	-0.656713744	0.356172406	0.007155593997778350000000000
AHSG	18.99707656	-0.700865335	0.418187336	0.007252706616791990000000000
KLHL35	10.46512731	0.773192434	0.37524241	0.007276789041298190000000000
LRRN3	556.0316324	0.634263778	0.233274845	0.007863092975779830000000000
MUC15	175.6656179	0.678962804	0.230759536	0.007865689219326370000000000
ART4	8.400531455	0.940250109	0.499651752	0.008118887840858550000000000
PRR5L	11.08960195	-0.84246787	0.505638814	0.008695202470939140000000000
SLC22A18	5.521597896	1.610740032	0.598106613	0.008713377442345090000000000
TLCD2	3.576731313	-1.797992479	0.810743207	0.008799426431674850000000000
NPTX2	63.6498589	-0.629113794	0.242121221	0.008854530008658120000000000
METRNL	22.90345178	-0.855099277	0.306624325	0.008888111799170290000000000
ZFP69B	38.42915336	-0.693271353	0.276554448	0.009078673844966860000000000
APOLD1	8.308417685	0.830308206	0.431354254	0.010086423026406900000000000
PTPN6	9.613966845	-0.749795573	0.550564124	0.010287823902171000000000000
SLC5A9	12.50720009	-0.985337836	0.5207923	0.010320132748930300000000000
NEURL1	5.800863266	-0.601284382	0.818050067	0.010611203375942900000000000
KCTD16	16.75489081	0.956057293	0.445383473	0.010930773580850100000000000
NMUR1	10.67480997	-0.768743024	0.384816903	0.011375613314739900000000000

NPC1L1	11.31956787	-0.705299439	0.441751321	0.0114047389784186000000000
SPTSSB	4.762382574	1.051245321	0.782919046	0.0115632636106985000000000
BOK	52.36395971	-0.668435918	0.307538094	0.0119647622130412000000000
GAL	77.27179727	-1.127677474	0.511812159	0.0121767733369912000000000
ETV4	6.202027684	-1.281192456	0.532175309	0.0124192917875909000000000
BEND6	18.65872254	0.665226502	0.322466846	0.0128451905319065000000000
TNFSF10	36.00587049	-0.605438782	0.550533867	0.0129494150481250000000000
PREX1	3.62419441	-1.528021129	0.875489966	0.0130428856353584000000000
H19	5.455273758	-1.838720821	0.636316904	0.0132496153704579000000000
TMEM95	7.075152694	1.044134078	0.430229508	0.0140364423650857000000000
TRO	4.085539716	-1.266113658	0.599268215	0.0151355679421154000000000
CCR10	5.633539212	-1.172322895	0.688483855	0.0153927912531017000000000
TRABD2B	9.0775482	-1.150286853	0.524467286	0.0168650207023417000000000
COL3A1	7.853623503	-0.909718086	1.200444738	0.0169489885274777000000000
SEPT4	33.18242092	-0.653843663	0.395451226	0.0178351121953250000000000
OMG	5.994737266	1.386327573	0.558419287	0.0181398151654661000000000
ATP1A2	5.94913336	-2.637356543	0.844681489	0.0184387513732152000000000
DRAM1	11.50528217	0.610042507	0.411339096	0.0188675269356067000000000
TACC2	43.19678746	-0.724989083	0.331509172	0.0193087616099669000000000
CA5A	12.89699819	0.623575799	0.930378741	0.0206563352352512000000000
MYH1	4.121786803	-0.767843219	0.784377813	0.0207718869145224000000000
SHISA9	10.93108463	-0.985436704	0.470297217	0.0218615325651679000000000
SLC10A6	5.034707986	-1.609984131	0.646246505	0.0223697689225512000000000
GPR137C	7.88615951	1.334154688	0.480651203	0.0231688731592513000000000
KLB	5.263166007	0.779999415	0.55114166	0.0235621262634181000000000
SNN	15.77322401	-0.72898986	0.43253244	0.0238410460992173000000000
AIF1	5.505448082	0.976228195	0.631402531	0.0259337989417400000000000
MICALL2	26.84518683	-1.16926238	0.425674096	0.0261675092915630000000000
MICAL2	16.47178506	-0.696969035	0.494572481	0.0262454244237049000000000
ADAMTS4	23.1130623	-1.41386222	0.678262384	0.0264277578004684000000000

PTPRB	11.98800286	-1.299259002	0.622653238	0.026741751859328400000000000
PHLDA1	110.7696818	-0.830181041	0.248407895	0.027205521353137500000000000
GRO1	30.25680723	0.775200884	0.410476598	0.028039393776829000000000000
EPHA1	4.745684128	-3.084415349	0.967439073	0.028063258674750000000000000
GPR18	14.45612659	-0.669058866	0.450851178	0.029637061788022600000000000
RTN2	49.34892567	-0.903077491	0.260306053	0.029665165065307000000000000
OTOL1	12.69500784	0.722305432	0.408488509	0.029845001696915100000000000
TUBB3	196.7423905	-0.769777367	0.259316689	0.030897961826433400000000000
HAVCR2	43.03520019	-0.615064687	0.262367805	0.031233923428189100000000000
CYP4F2	8.099777036	0.973228121	0.569106536	0.031687058144656200000000000
KRT77	31.58452064	0.606644025	0.311024933	0.031852219440476900000000000
CNTF	6.355104044	-1.1178149	0.538652975	0.031889587626088000000000000
GJA4	12.26283972	-1.236504328	0.435936138	0.032499826671644300000000000
CDH16	4.707676717	-1.2934951	0.632824839	0.033494536615196200000000000
MSX1	7.737405187	-1.202769714	0.449082085	0.033725438594498900000000000
ZNF157	4.668732005	-0.616209583	0.670971425	0.033804894607959000000000000
TRAF5	19.28326301	-1.497700162	1.134135529	0.034243392512039100000000000
HOXA4	33.26871779	-0.876554961	0.665790326	0.035640927398658800000000000
C8H9orf84	15.34914168	0.678179019	0.382982187	0.036492598842970800000000000
STAC2	6.68569013	-1.172241627	0.449406673	0.036763757041282600000000000
NECTIN4	4.789937902	-1.095622464	0.724203936	0.037195130296535800000000000
FOSB	20.4640533	-1.146045031	0.380838618	0.037378678528886200000000000
CACNA1D	7.147922746	-0.808478815	0.408262357	0.037505827549447600000000000
NAIP	10.44547745	0.670245788	0.373684557	0.039244611443489400000000000
TMEM130	12.99675217	0.79900798	0.563073852	0.039912215173822700000000000
GGT7	18.72775583	-0.65857785	0.386784071	0.040034528632740600000000000
CDH4	10.42476884	-0.761555156	0.435929593	0.041128624949153800000000000
MMP9	302.8709583	-0.602864901	0.672173945	0.041632514314381200000000000
SLC7A14	4.883298132	-1.643548713	0.695495119	0.041878057499869100000000000
ZBTB32	16.11526421	-0.697251407	0.330405937	0.041878057499869100000000000

CATSPER3	5.841706367	1.069974409	0.676000608	0.0420084499976428000000000
PIGZ	5.261181738	-1.048288972	0.570329635	0.0425189317419449000000000
GGT5	8.199628256	0.675759141	0.511830043	0.0445939879528860000000000
C2CD4B	3.923788434	-1.377197201	0.904173574	0.0446158143211749000000000
CYP2C19	9.479346627	1.21310583	0.528811338	0.0453107346975676000000000
GGTA1	18.36592426	0.774220853	0.41717866	0.0458691714692268000000000
CXCL3	74.9768611	0.876703123	0.299705487	0.0467322677128319000000000
SPATA13	15.84818915	-1.277589412	0.454091696	0.0467754704504581000000000
ARL13A	14.01102755	0.775387096	0.442010688	0.0488148224775030000000000
RAMP2	14.20678088	-0.803021234	0.423534296	0.0498279046207272000000000
