# Supplementary material for: Extracellular Vesicles Derived From Antral Follicles Significantly Change the Transcriptional Profile of Cumulus Cells and Oocytes During Pre‐In Vitro Maturation in Cattle
Source: Mol Reprod Dev. 2025 Nov 24;92(11):e70068. doi: 10.1002/mrd.70068 (PMC12645189; doi:10.1002/mrd.70068)
Supplement: Supplementary file 5 — Table S4: Differentially expressed genes in oocytes of Control vs. Early EVs. [file MRD-92-e70068-s007.pdf]

Table S4. Differentially expressed genes in oocytes of Control vs. Early EVs.

Gene	baseMean	log2FoldChange	lfcSE	padj
LOC619094	14.49591992	-3.108772011	0.691403753	0.000000000000
SERPINA6	380.5654513	0.647926078	0.213997504	0.000000012623
SMOC1	345.2636489	-1.102392989	0.174706821	0.000000111887
PLPP4	131.2641313	0.602901015	0.202009972	0.000000271130
PLEKHA4	47.4171043	-0.911789495	0.457926615	0.000000997242
LOC112444504	18.84359962	2.260900088	0.513719234	0.000009893075
LOC112446356	74.13922641	0.713883732	0.321117174	0.000015444543
CRABP2	37.48267887	-0.797099117	0.307253641	0.000104407144
TRIM56	30.85709097	-0.971177126	0.48162598	0.000127963853
BoLA	52.64079542	-0.889444479	0.68640437	0.000178132680
SOX2	59.30804717	-1.013198161	0.350592334	0.000214311481
LOC112443817	11.18234584	2.395756249	0.656670767	0.000224427352
CDK6	134.8926706	-0.634206975	0.477360453	0.000284934661
LOC104971363	33.07379828	-0.825261785	0.373499512	0.000292408267
LOC112447891	22.91275115	0.6430141	0.308497417	0.000312003816
ANXA1	16.64583688	0.639875766	0.563064952	0.000356109172
LOC112442303	26.90095477	-0.789021245	0.488856346	0.000360800171
LOC112449553	16.04247712	-1.111334544	0.486044267	0.000360800171
RMI2	58.10951519	-0.704655395	0.238894802	0.000635527818
RHOB	49.55174413	-0.877726895	0.244046682	0.000636964038
LOC104972417	41.3413841	0.951233726	0.389273961	0.000666280472
ITM2B	48.5768482	-0.738895742	0.338158183	0.000677500888
TNFSF18	65.53410154	0.730511594	0.268777245	0.000704769324
CCND2	22.28730227	-1.608105428	0.647772037	0.000771250676
MIC1	59.67424472	-0.968813464	0.715410384	0.000782810472
CAMK1G	24.9098946	0.838814516	0.388237887	0.000844867582
C2CD2	34.92467382	1.172283977	0.328364841	0.000899789909
HMGN5	44.21633278	-1.063989233	0.449262895	0.000954793639

GHR	268.7181462	0.603571758	0.137217461	0.001001065252
DYNC1H1	375.0255132	-0.71590138	0.297692099	0.001049563972
CCDC40	350.880093	0.603087161	0.180263903	0.001139550191
CBLN4	27.6640022	0.709470226	0.299270075	0.001606611238
LMO2	50.80606036	-0.77799149	0.272790181	0.001696798897
CSH2	140.9352179	0.621556188	0.277673193	0.001747980639
CCDC107	334.7203648	-1.165746199	0.238916742	0.001868294600
C16H1orf115	58.04616657	-1.358760452	0.64827286	0.001885212556
PCYOX1	14.2850325	0.929037659	0.442436685	0.002053384831
LOC112448271	38.07496473	0.776602626	0.293692165	0.002195164133
LOC112447840	66.85767281	-1.153159932	0.348670456	0.002923783454
TMEM176B	11.91486485	1.379122523	0.680198643	0.003024373128
UPK1A	23.54089281	-0.661310431	0.375625777	0.003451710192
CNN2	75.08541373	-0.773071543	0.240441053	0.003516691756
DDIT4	48.74097619	-0.946331493	0.296327566	0.003523408245
CACNB4	1150.024433	0.629404905	0.294675844	0.003775751531
SLC24A5	14.07978641	-0.963108969	0.485875619	0.004185310809
CUEDC1	53.7613319	-0.833843523	0.378681778	0.004193948630
AFG3L2	1577.820302	-0.637991908	0.225596185	0.004278142045
CDX2	14.48908495	-1.510882034	0.574533895	0.004351088332
INPP5F	1319.766085	-0.861656293	0.268661819	0.004388474987
PPM1J	24.59460517	-0.838397545	0.371861928	0.004388474987
FOLR1	13.51016153	-2.423917335	1.165669526	0.004437027811
EIF4G1	135.5458812	-0.668283061	0.385335558	0.004770538152
LOC786942	17.14643837	-1.291162323	0.642323053	0.004770538152
KIAA1210	41.36553693	0.765644712	0.294676371	0.005014538420
MGC137055	476.4864747	0.669666576	0.252926113	0.005014538420
C16H1orf53	156.5809982	-0.750455507	0.264502647	0.005124897843
DNLZ	113.0844115	-1.318924422	0.269598992	0.005212832435
LOC616860	45.06881734	-0.660481181	0.454588238	0.005213679137

CCDC137	105.8649735	-0.602637091	0.229262449	0.005314587312
ICE1	244.5614164	-1.033033194	0.342977481	0.005359444008
FAM216B	17.84154097	-0.62996452	0.444365588	0.005383153463
LOC112445749	153.9261873	0.825657356	0.272034097	0.005978172235
RAMP2	34.30377935	-1.253114176	0.309389792	0.005997410006
MYEF2	38.01204609	-0.809608457	0.319375297	0.006039670605
LOC785899	10.99780705	1.00742437	0.423281511	0.006763444758
LOC112443004	185.2790117	0.706064194	0.169337114	0.006793488940
C1H3orf52	12.27848666	-0.693785633	0.412094796	0.007047700448
RN18S1	16655.90122	-0.640554185	0.476380349	0.007311491181
IFI6	29.05929421	-0.891548404	0.685528217	0.007325766717
LOC505072	27.51551192	-0.603542474	0.533991352	0.007487693110
WDR1	163.0231729	-0.884402646	0.235428143	0.007682333549
UBN1	241.7677785	-0.625785957	0.333775888	0.007705766813
TMEM88B	40.19542248	-1.226490567	0.296430019	0.007993446606
CD81	397.6262571	-0.635811537	0.18828612	0.008010062549
CRACR2A	13.65226078	-1.612033539	0.528316276	0.008416111400
MTHFD2L	23.65923156	2.141808587	0.766200033	0.008985367396
PSMB10	19.49311053	-0.734922854	0.40543849	0.009874499518
DCT	17.0559558	0.789976665	0.636971033	0.010024806647
ACADVL	108.6107767	-0.669501029	0.283722221	0.010521417766
CARS2	124.34696	-0.671818473	0.269834086	0.010795558928
LOC104969408	50.78946456	0.683689242	0.278770663	0.010998062524
LOC107132680	25.68418483	1.297576498	0.386840665	0.011425062918
LOC616092	51.7883759	-0.693458396	0.379580549	0.012414303776
NKX6-2	30.34492182	-1.535607314	0.397383872	0.013311577725
SH2B2	40.01482306	-1.09150926	0.33050301	0.013440543567
LOC104973099	85.76778899	-0.664721375	0.337792604	0.013622901669
CDKN2A	37.4853337	0.8486714	0.427361142	0.013931054604
LOC112444876	26.88695451	-0.77803903	0.380817569	0.014182263163

NENF	222.8880251	-0.697871607	0.173780799	0.014568400891
MRPL36	330.4544307	-1.679174457	0.436033592	0.014828162199
GADD45B	22.49040253	-0.715622364	0.373894383	0.014909528094
APOH	18.73165013	0.642605838	0.378705229	0.014960717299
ANTXR2	93.76550832	-0.830893248	0.3016414	0.014963668234
LOC112443751	221.7515218	0.770055289	0.347847571	0.015678063487
USP36	673.6391264	-0.628864149	0.321921808	0.015743909082
LOC112442026	13.01158087	1.325307566	0.461696523	0.015870649718
ZNF350	33.71224754	-0.679299685	0.324954143	0.016012235108
ITPKA	22.52698589	-0.623084726	0.386067906	0.016427149169
GAB2	202.650808	-0.765694245	0.242681553	0.016851938070
LOC112445863	95.68649072	1.021952996	0.992095733	0.017028469310
CHST4	76.24340545	-0.734636003	0.278205471	0.018076586639
DDB1	146.6756721	-0.728090921	0.332043847	0.018406820273
LOC101907577	23.57761786	0.757768438	0.348436543	0.018743233394
ASPN	25.65004481	0.682084048	0.292940994	0.019412793263
LOC104973884	419.9469585	-0.687590872	0.187013988	0.019588849165
MZT2B	16.40945923	-0.962971396	0.391111428	0.020817477028
CIB3	22.44666582	0.99773703	0.363408171	0.021446515438
LOC101907322	73.73361233	0.731149704	0.317946486	0.021727003619
C6H4orf48	30.34809241	-1.854125891	0.52172439	0.021945999554
AKAP4	16.50778675	1.662087806	0.909074671	0.022325421441
RABL6	40.74223313	-0.91235771	0.345622136	0.022635509449
SUPT6H	149.475558	-0.657211317	0.30951848	0.022932321239
H4	144.1769854	-0.615714083	0.254201215	0.023239576221
CITED2	384.276221	-1.043548595	0.462184573	0.023310755790
LOC507550	23.69358331	-1.455039395	0.356726615	0.023659657231
LOC511498	22.16669191	0.640484315	0.360831595	0.024526140347
DNAJC5	94.73147077	-0.977609407	0.258772978	0.024734579709
INPPL1	18.37713357	-1.569594527	0.39811223	0.025164903360

DGKZ	145.9159563	-0.690820314	0.325822208	0.025262075391
LOC112449072	44.87485307	1.036184514	0.355511023	0.025683853444
ZC3H18	188.6246249	-0.710031021	0.292317875	0.025757588413
AP3D1	137.1893265	-0.682354344	0.356047077	0.025936703454
LINC00483	1674.926088	0.649444866	0.24856943	0.026062027799
BRD3	641.5598047	-0.627752982	0.298450159	0.026094482527
DYNLL2	879.160044	-0.89814559	0.228219689	0.026816189931
DUSP28	75.42271713	-0.843123716	0.30795058	0.026893812263
LOC518768	171.4936388	-0.662599089	0.305679876	0.027824305323
CENPB	24.8796203	-0.733386984	0.536757733	0.028454547092
ZNF628	15.4029829	-1.869020689	0.520128429	0.028835191326
TMEM119	32.31337826	-0.794485175	0.441487599	0.030077212797
SGIP1	30.46428375	-0.971857968	0.287082955	0.030846743326
LOC112443236	155.5403938	1.836612746	0.685114947	0.032798938969
PPFIA1	587.7473705	-0.890826382	0.441678616	0.032880875419
LOC508153	155.6295819	1.837904051	0.686278741	0.033104295905
CBR4	100.4310499	0.613754955	0.288668044	0.033538391817
MT2A	37.95747175	-0.732358786	0.32008463	0.033596893742
LOC107132308	94.98023005	0.613472583	0.217564447	0.033643578503
TDH	11.54504668	-0.723209528	0.469322148	0.033643578503
FBXO31	171.6245094	-0.810479951	0.304208668	0.034351027427
RANBP1	1098.873077	-0.715020432	0.256951118	0.035248262600
LOC101906779	187.0059049	0.704703888	0.306147671	0.035549267175
ANKRD29	13.11252268	-1.001144589	0.410812732	0.036564583023
LOC101904517	156.516679	1.486919587	0.656657505	0.036683937121
UBE2E1	1317.800659	-0.671872293	0.332358972	0.037939340282
C1D	213.8333813	0.633069421	0.243425475	0.038600007375
TMEFF2	38.31897511	-1.086787964	0.318041439	0.038792624875
RARRES1	623.6747621	-0.821096433	0.255361315	0.038966039683
CTCFL	34.93904593	0.87768381	0.362247327	0.039916407212

TMEM140	59.55031507	-0.809450293	0.323840539	0.040861246698
IMPG1	1142.821693	0.603845708	0.415259496	0.040978468642
CCDC198	38.98328392	0.687171249	0.323242085	0.041596106759
MON1A	54.05871205	0.637017596	0.299835211	0.041727492304
CTDP1	64.82650603	-0.604058987	0.262720336	0.042168984664
HSF1	75.37493052	-1.064343892	0.322933016	0.043838820826
MME	19.89130886	1.976298292	0.492545372	0.044272254346
GAK	105.4286968	-0.723280862	0.322506991	0.044295422073
LOC516108	29.04964694	-0.676156559	0.320698281	0.044679985966
RASSF4	49.69765714	0.89628636	0.294869901	0.045984153882
FGF10	24.32930548	-0.866881617	0.428531297	0.046011681071
GSKIP	108.1491565	1.181235597	0.361229882	0.046413015438
ESPN	69.81252615	-0.768597364	0.2503849	0.046491230814
MRM1	108.1036231	-0.695542836	0.302636025	0.046618763497
DEPP1	49.29023613	0.898922607	0.296042773	0.047008577700
ALS2CL	24.83561715	0.646670689	1.174284906	0.047153543600
BRD9	389.4104059	-0.652733937	0.280738551	0.047636441335
ARRDC4	424.8595887	-0.769948321	0.627797697	0.047991601232
CBS	120.1579531	-0.650259568	0.298454854	0.048344363687
KANSL3	236.5716655	-0.673816871	0.272689165	0.048344363687
ENPP6	31.67948847	0.850863344	0.407020108	0.049586734406
FST	16.69768085	-0.650023706	0.5092513	0.049586734406
HRAS	203.7151291	-0.933960381	0.313676651	0.049896989270
PTH2	17.74914465	-1.390546042	0.511001537	0.049896989270
