# Supplementary material for: Extracellular Vesicles Derived From Antral Follicles Significantly Change the Transcriptional Profile of Cumulus Cells and Oocytes During Pre‐In Vitro Maturation in Cattle
Source: Mol Reprod Dev. 2025 Nov 24;92(11):e70068. doi: 10.1002/mrd.70068 (PMC12645189; doi:10.1002/mrd.70068)
Supplement: Supplementary file 6 — Table S5: Differentially expressed genes in oocytes of Control vs. Late EVs. [file MRD-92-e70068-s004.pdf]

Table S5. Differentially expressed genes in oocytes of Control vs. Late EVs.

Gene	baseMean	log2FoldChange	lfcSE	padj
LOC619094	14.49591992	-2.956316102	0.69475402	0.0000000000000000
NUGGC	74.88689644	-0.66836018	0.23761717	0.0000000000034391
LOC786372	53.37082195	-0.866158989	0.43372074	0.0000000000525742
SMOC1	345.2636489	-0.756006737	0.17544654	0.0000001118869424
PLEKHA4	47.4171043	-0.991462613	0.45812923	0.0000009972422062
COX8B	39.72166522	-0.632699394	0.42579183	0.0000071727381960
LOC1124445	18.84359962	1.397431849	0.50015684	0.0000098930746104
LOC514978	14.39196028	1.160241721	1.16489464	0.0000154445432434
ELAVL3	38.08905908	-0.620388412	0.4420551	0.0000162626584262
CRABP2	37.48267887	-0.630269809	0.30974044	0.0001044071442807
LHFPL1	17.93906085	0.972188454	0.4464438	0.0001389975747540
LOC509034	129.1056277	-0.906690756	0.36796871	0.0001742649251397
BoLA	52.64079542	-0.870033757	0.68729234	0.0001781326796156
LOC1124438	11.18234584	2.505039144	0.66619823	0.0002244273518329
CDK6	134.8926706	-0.746005835	0.47748834	0.0002849346609295
LOC1124478	22.91275115	1.104745129	0.31835162	0.0003120038155445
LOC1124495	16.04247712	-0.812511323	0.49168573	0.0003608001705305
RMI2	58.10951519	-0.817097936	0.23947985	0.0006355278178949
RHOB	49.55174413	-0.612024855	0.24727906	0.0006369640381649
ITM2B	48.5768482	-0.788302757	0.33866685	0.0006775008876828
TNFSF18	65.53410154	0.823276328	0.27034135	0.0007047693241080
CCND2	22.28730227	-1.802174707	0.64775558	0.0007712506760752
MIC1	59.67424472	-0.920051702	0.7163103	0.0007828104718542
CAMK1G	24.9098946	0.822645094	0.39024318	0.0008448675821298
C2CD2	34.92467382	1.424447193	0.33296616	0.0008997899093364
HMGN5	44.21633278	-0.86393441	0.45099575	0.0009547936386222
FCHO2	83.16562971	0.616998719	0.27580319	0.0015576530343222
CBLN4	27.6640022	0.677782334	0.30095842	0.0016066112380999

ZNF365	28.13170522	0.605659481	0.42917546	0.0016196462091007
OAS1Y	13.81806726	-0.628049761	0.67009927	0.0016467650568551
PIH1D3	20.71871575	0.606589428	0.45338857	0.0016467650568551
LMO2	50.80606036	-0.612980631	0.27480607	0.0016967988966749
CSH2	140.9352179	0.941599468	0.27893945	0.0017479806393863
CCDC107	334.7203648	-1.144992593	0.239131	0.0018682945999217
C16H1orf115	58.04616657	-1.032900397	0.64993586	0.0018852125558489
PCYOX1	14.2850325	0.996229938	0.44737678	0.0020533848305328
CD3G	897.6202453	-0.668243279	0.47062291	0.0023901304353836
PLEKHS1	40.79651436	-0.975450855	0.40819976	0.0026195137330878
UPK1A	23.54089281	-0.673015043	0.37744828	0.0034517101918698
CNN2	75.08541373	-0.78978079	0.24134486	0.0035166917555051
LRP1	42.65995753	0.85615795	0.34522495	0.0036840764718397
CACNB4	1150.024433	0.837391185	0.29480955	0.0037757515306047
C22H3orf14	304.3390116	-0.64314028	0.16939992	0.0042365470680251
CDX2	14.48908495	-1.022413678	0.58212225	0.0043510883323668
INPP5F	1319.766085	-0.731248758	0.26875908	0.0043884749872057
PPM1J	24.59460517	-0.75423241	0.37444268	0.0043884749872057
SAMD5	66.17060149	1.198026713	0.27086376	0.0043884749872057
FOLR1	13.51016153	-1.885953391	1.17315608	0.0044370278109462
CFI	57.84534367	0.791221609	0.53845824	0.0044650374229984
LOC786942	17.14643837	-1.065823271	0.6455099	0.0047705381524521
KIAA1210	41.36553693	0.967687566	0.29853716	0.0050145384201901
C16H1orf53	156.5809982	-0.858091792	0.26461018	0.0051248978434909
DNLZ	113.0844115	-1.171150161	0.27048102	0.0052128324349242
LOC616860	45.06881734	-0.774164251	0.45462806	0.0052136791370992
ICE1	244.5614164	-0.881349167	0.34343971	0.0053594440075169
MROH7	14.76967221	0.686348125	0.50646699	0.0055835890563223
LOC1124457	153.9261873	0.890242861	0.27260096	0.0059781722349872
RAMP2	34.30377935	-1.287927686	0.31051463	0.0059974100062085

IFI6	29.05929421	-1.751806881	0.68127009	0.0073257667173556
LOC505072	27.51551192	0.816033338	0.54692219	0.0074876931099823
WDR1	163.0231729	-0.709527539	0.23625327	0.0076823335492657
TMEM88B	40.19542248	-0.758552036	0.3010567	0.0079934466056327
LOC1124418	20.32839459	0.606014934	0.48501236	0.0081893055895467
MTHFD2L	23.65923156	3.199463854	0.78120458	0.0089853673962216
PSMB10	19.49311053	-0.792166486	0.40661793	0.0098744995181123
LOC1124434	46.62754856	-0.767605045	0.35390898	0.0099663123309686
DCT	17.0559558	-0.868859022	0.61904076	0.0100248066466674
SLC7A11	63.08124257	1.010991922	0.25500236	0.0101807451711974
LOC1071326	25.68418483	0.854220337	0.38385651	0.0114250629176190
LOC616092	51.7883759	-0.924580359	0.37921126	0.0124143037764560
NKX6-2	30.34492182	-1.112312366	0.4017179	0.0133115777253088
SH2B2	40.01482306	-1.12928025	0.33145789	0.0134405435666751
PDAP1	284.6673807	0.612588992	0.30354489	0.0136726012523244
IFITM3	41.13133567	-1.190223918	0.61889689	0.0142381194597810
LOC1019044	20375.04456	0.602661641	0.23392994	0.0143827621138283
NENF	222.8880251	-0.621128279	0.1743741	0.0145684008908936
MRPL36	330.4544307	-1.445198144	0.43631462	0.0148281621988076
ANTXR2	93.76550832	-1.192961512	0.30124036	0.0149636682336126
LOC509854	130.9491647	0.6998941	0.21475357	0.0150715522641806
ARMCX3	29.08781828	-0.644761521	0.468026	0.0154872941752421
ITPKA	22.52698589	-1.075183906	0.38339438	0.0164271491690879
LOC1124473	21.46121351	1.008169861	0.31382267	0.0169931253098319
LOC1124458	95.68649072	-1.935588713	0.98634681	0.0170284693104007
LOC1008513	50.76206724	-1.03803231	0.44372212	0.0172342944613455
CCP110	880.9094244	0.606668937	0.23436461	0.0185249081528350
HRCT1	52.49626685	-0.891655981	0.30236599	0.0196232825761359
CIB3	22.44666582	0.904042474	0.36450927	0.0214465154375351
LOC615451	56.86791329	-0.695199276	0.30293831	0.0215587106741654

C6H4orf48	30.34809241	-1.822535426	0.52290634	0.0219459995538545
KIAA1024L	35.06074878	-0.66946093	0.37950942	0.0219459995538545
AKAP4	16.50778675	-0.788524247	0.88133856	0.0223254214413919
SIVA1	952.6318738	-0.615164657	0.28267005	0.0229236678974254
CITED2	384.276221	-0.895188712	0.46232753	0.0233107557897085
LOC1019040	22.54954518	0.842664742	0.31370492	0.0236596572313827
LOC507550	23.69358331	-1.337646554	0.35950372	0.0236596572313827
DNAJC5	94.73147077	-0.895803693	0.25975625	0.0247345797087072
LOC1124431	12.83702135	0.602492874	0.55867407	0.0250921203811773
INPPL1	18.37713357	-0.828698309	0.40830518	0.0251649033598257
LOC1124490	44.87485307	1.224140369	0.35805072	0.0256838534443007
HIST1H4D	34.23287944	-0.741484271	0.43561588	0.0258989909283144
NMRK2	59.07538431	-1.115522247	0.36305606	0.0266412425093210
C5H12orf66	229.4700834	0.641181135	0.18445987	0.0279329484848552
H2AFX	235.7912764	-0.66520418	0.19613612	0.0279329484848552
ZNF628	15.4029829	-1.169064993	0.52919138	0.0288351913263070
LOC1124460	60.47031424	0.910020278	0.32001225	0.0300088060646764
LOC1003371	21.62626955	0.816835894	0.36822304	0.0322742409586891
LOC1124432	155.5403938	1.155312991	0.68438563	0.0327989389689400
LOC508153	155.6295819	1.156789099	0.68555088	0.0331042959045889
LOC1124442	24.86958255	0.709877423	0.367424	0.0333260295478044
CBR4	100.4310499	0.79835862	0.29025908	0.0335383918173285
MT2A	37.95747175	-0.610029362	0.32216933	0.0335968937420917
TDH	11.54504668	-0.996172608	0.4679993	0.0336435785032360
LOC1124446	12.37927776	0.699790306	0.40464418	0.0348110964125327
FXN	145.8899185	-0.639759287	0.21498012	0.0356470242307672
LOC1019045	156.516679	2.161127492	0.65788951	0.0366839371212958
CTCFL	34.93904593	1.01357367	0.36547147	0.0399164072121025
IMPG1	1142.821693	0.618633941	0.41531365	0.0409784686423095
LOC1019038	42.81305301	-0.844066497	0.35161584	0.0413167593764568

CCDC198	38.98328392	0.709889661	0.3248141	0.0415961067587490
PTER	23.45128174	0.698814094	0.34206237	0.0429669034574748
HSF1	75.37493052	-0.661655677	0.32513468	0.0438388208263688
MME	19.89130886	1.550391591	0.48966322	0.0442722543459059
LOC516108	29.04964694	-1.125843083	0.31840786	0.0446799859655210
LOC1019029	243.3394338	0.989602053	0.51371819	0.0448371508168434
FGF10	24.32930548	-0.903771397	0.42979313	0.0460116810707113
GSKIP	108.1491565	1.304298662	0.36220294	0.0464130154379488
LOC618696	25.99744704	-1.209582672	0.55781746	0.0467589787855508
ALS2CL	24.83561715	1.256535189	1.18826367	0.0471535435998493
DEAF1	20.53337209	0.733539222	0.41587759	0.0473250099334322
LOC1124427	197.7122993	0.759267433	0.27895386	0.0495283926714684
FST	16.69768085	-0.745502367	0.50998946	0.0495867344063989
ELF2	151.7710233	0.601134603	0.20502563	0.0498969892695866
HRAS	203.7151291	-0.610645925	0.31446464	0.0498969892695866
PTH2	17.74914465	-1.083157315	0.51536711	0.0498969892695866
