# Supplementary material for: Extracellular Vesicles Derived From Antral Follicles Significantly Change the Transcriptional Profile of Cumulus Cells and Oocytes During Pre‐In Vitro Maturation in Cattle
Source: Mol Reprod Dev. 2025 Nov 24;92(11):e70068. doi: 10.1002/mrd.70068 (PMC12645189; doi:10.1002/mrd.70068)
Supplement: Supplementary file 7 — Table S6: Differentially expressed genes in oocytes of Early EVs vs. Late EVs. [file MRD-92-e70068-s003.pdf]

Table S6. Differentially expressed genes in oocytes of Early EVs vs. Late EVs.

Gene	baseMean	log2FoldChange	lfcSE	padj
NUGGC	74.88689644	-0.680079185	0.190921874	0.000000000003
MORC4	244.3844134	-0.764308428	0.173357096	0.000000000163
LOC1124460	72.59303879	-0.772800454	0.227970172	0.000000061580
LOC1124445	18.84359962	-0.863468239	0.443537449	0.000009893075
LOC784305	181.8139227	0.694190532	0.231782606	0.000012311541
LOC514978	14.39196028	0.663092306	0.965713694	0.000015444543
TRIM56	30.85709097	0.683850272	0.382642784	0.000127963853
ZCCHC2	362.0420873	0.663495409	0.254260471	0.000135058528
NLGN4X	352.3277379	-0.766352953	0.272689083	0.000151463061
SLC40A1	724.8791312	-0.858829506	0.276380654	0.000160128542
PARD6B	140.0625998	0.663184369	0.286848733	0.000206208399
LOC1124423	26.90095477	0.89265615	0.397054693	0.000360800171
LOC1049724	41.3413841	-0.708550702	0.32118645	0.000666280472
CHD8	257.0102662	0.707304716	0.262088444	0.001028251342
RND3	12.24131912	-0.832629768	0.496355897	0.001437372686
FAM183A	55.06117656	-0.648839251	0.242197374	0.001646765057
OAS1Y	13.81806726	-1.033857538	0.54740537	0.001646765057
LOC1124482	38.07496473	-0.627439595	0.242635217	0.002195164133
LOC1049690	20.28531371	0.629739179	0.295063392	0.002759258550
LOC1124478	66.85767281	0.935914257	0.280625371	0.002923783454
TMEM176B	11.91486485	-1.180861394	0.565607596	0.003024373128
TBX18	42.10703434	0.680253115	0.190617621	0.003419039261
LRP1	42.65995753	0.829453367	0.283009616	0.003684076472
SLC24A5	14.07978641	0.679416891	0.380284725	0.004185310809
CUEDC1	53.7613319	0.616825165	0.305159347	0.004193948630
SAMD5	66.17060149	0.789653709	0.224644533	0.004388474987
CFI	57.84534367	0.892708316	0.439984768	0.004465037423
EIF4G1	135.5458812	0.686507842	0.313965344	0.004770538152

PPP3R1	26.1650143	0.619350095	0.277349259	0.004778472540
MGC137055	476.4864747	-0.783843928	0.206562963	0.005014538420
MROH7	14.76967221	1.057821494	0.409236073	0.005583589056
PROM1	70.74556682	0.620885756	0.186708575	0.007002818993
C1H3orf52	12.27848666	0.946868889	0.337153934	0.007047700448
CCL26	25.33837393	-0.645580069	0.264549262	0.007181951876
RN18S1	16655.90122	0.801961386	0.388964003	0.007311491181
IFI6	29.05929421	-0.860258477	0.533487699	0.007325766717
LOC505072	27.51551192	1.419575812	0.444327381	0.007487693110
CRACR2A	13.65226078	1.578785001	0.418350696	0.008416111400
MTHFD2L	23.65923156	1.057655267	0.657293582	0.008985367396
NES	238.5151063	0.70414108	0.370246099	0.009509848030
PGR	20.50280591	-0.638981313	0.36287007	0.009511717054
LOC1124434	46.62754856	-0.727274482	0.285072259	0.009966312331
DCT	17.0559558	-1.658835687	0.51379491	0.010024806647
SLC7A11	63.08124257	0.608628252	0.211715746	0.010180745171
LOC1049730	85.76778899	0.872135992	0.275835611	0.013622901669
LOC1019045	28.4978814	0.738884873	0.268676521	0.013898572339
CDKN2A	37.4853337	-0.873664192	0.350664889	0.013931054604
IFITM3	41.13133567	-0.704507683	0.495751285	0.014238119460
SETD5	86.54806788	0.629435695	0.267001376	0.014541954629
GADD45B	22.49040253	0.719718886	0.300139983	0.014909528094
APOH	18.73165013	-0.774684203	0.309082587	0.014960717299
LOC1124437	221.7515218	-0.687009291	0.284452694	0.015678063487
LOC1124420	13.01158087	-0.862472644	0.391360501	0.015870649718
LOC1124458	95.68649072	-2.957541709	0.807315647	0.017028469310
LOC1049696	56.34944603	-0.963708353	0.636046768	0.018270230639
RGSL1	30.42328324	-0.738425832	0.398309389	0.018329397571
LOC1124493	184.6681595	0.658415138	0.242209402	0.018587068240
LOC1071314	13.51402736	1.471875228	0.418806693	0.018805340492

HRCT1	52.49626685	-0.648440609	0.240315835	0.019623282576
TNFSF4	19.07825861	-0.668598722	0.343581419	0.020728075260
MZT2B	16.40945923	0.749789626	0.306188966	0.020817477028
AKAP4	16.50778675	-2.450612053	0.744785451	0.022325421441
RABL6	40.74223313	0.958417486	0.279582135	0.022635509449
LOC511498	22.16669191	-0.759984091	0.295266034	0.024526140347
LOC1124431	12.83702135	1.026910507	0.44979258	0.025092120381
INPPL1	18.37713357	0.740896218	0.290360445	0.025164903360
DSEL	15.17438256	0.66726172	0.298993068	0.025599434220
HIST1H4D	34.23287944	-0.656455917	0.349649623	0.025898990928
AP3D1	137.1893265	0.731322179	0.290141625	0.025936703454
TAF3	381.0254875	0.644368448	0.242799274	0.027015634950
ZNF628	15.4029829	0.699955696	0.377772528	0.028835191326
ATPAF2	224.1716858	-0.668390265	0.203171159	0.029282360090
TMEM119	32.31337826	0.798840664	0.357542856	0.030077212797
SGIP1	30.46428375	0.647334714	0.222425495	0.030846743326
LOC1124432	155.5403938	-0.681299754	0.561312743	0.032798938969
PPFIA1	587.7473705	0.800507273	0.36039308	0.032880875419
LOC508153	155.6295819	-0.681114952	0.562261597	0.033104295905
LOC1124442	24.86958255	0.86705828	0.299436144	0.033326029548
LOC1001399	435.7327828	0.645099489	0.207715689	0.034428305173
THRA	17.02571969	0.775734731	0.356606969	0.035524662758
MET	39.86052043	-0.681234298	0.251201153	0.035952484933
LOC1019045	156.516679	0.674207905	0.53916337	0.036683937121
TMEFF2	38.31897511	0.611011482	0.24861253	0.038792624875
TMEM140	59.55031507	0.738780448	0.261897301	0.040861246698
RASSF4	49.69765714	-0.837449775	0.242974573	0.045984153882
LOC618696	25.99744704	-0.90336848	0.445330212	0.046758978786
DEPP1	49.29023613	-0.828242244	0.244023599	0.047008577700
ALS2CL	24.83561715	0.609864501	0.979537004	0.047153543600

DEAF1	20.53337209	0.833533419	0.340067943	0.047325009933
ENPP6	31.67948847	-0.771540059	0.334564497	0.049586734406
